# Genetic Knockouts Indicate That the ABCC2 Protein in the Bollworm *Helicoverpa zea* Is Not a Major Receptor for the Cry1Ac Insecticidal Protein

**DOI:** 10.3390/genes12101522

**Published:** 2021-09-28

**Authors:** Omaththage P. Perera, Nathan S. Little, Heba Abdelgaffar, Juan Luis Jurat-Fuentes, Gadi V. P. Reddy

**Affiliations:** 1 Southern Insect Management Research Unit, USDA, Agricultural Research Service, Stoneville, MS 38776, USA; nathan.little@usda.gov (N.S.L.); gadi.reddy@usda.gov (G.V.P.R.); 2 Department of Entomology and Plant Pathology, University of Tennessee, Knoxville, TN 37996, USA; habdelga@utk.edu (H.A.); jurat@utk.edu (J.L.J.-F.)

**Keywords:** *Helicoverpa zea*, bollworm, CRISPR, Cry1A, Bt toxin, genome editing, knockout, functional genomics, resistance, ATP binding cassette transporter, ABCC2

## Abstract

Members of the insect ATP binding cassette transporter subfamily C2 (ABCC2) in several moth species are known as receptors for the Cry1Ac insecticidal protein from *Bacillus thuringiensis* (Bt). Mutations that abolish the functional domains of ABCC2 are known to cause resistance to Cry1Ac, although the reported levels of resistance vary widely depending on insect species. In this study, the function of the *ABCC2* gene as a putative Cry1Ac receptor in *Helicoverpa zea*, a major pest of over 300 crops, was evaluated using CRISPR/Cas9 to progressively eliminate different functional ABCC2 domains. Results from bioassays with edited insect lines support that mutations in *ABCC2* were associated with Cry1Ac resistance ratios (RR) ranging from 7.3- to 39.8-fold. No significant differences in susceptibility to Cry1Ac were detected between H. zea with partial or complete ABCC2 knockout, although the highest levels of tolerance were observed when knocking out half of ABCC2. Based on >500–1000-fold RRs reported in similar studies for closely related moth species, the low RRs observed in *H. zea* knockouts support that ABCC2 is not a major Cry1Ac receptor in this insect.

## 1. Introduction

The entomopathogenic bacterium *Bacillus thuringiensis* (Bt) produces Cry insecticidal proteins which are the main active components of sprays and are expressed by transgenic crop plants for environmentally sensitive pest control. The *cry1Ac* gene was first commercialized in the USA in 1996 as a plant incorporated protectant (PIP) in cotton [1], and since then has also been commercialized or authorized as PIP in corn (1997), eggplant in Bangladesh (2013) and the Philippines (2021), rice in China (2009) and the USA (2018), and soybean in several countries [2]. The main threat to the sustainable use of Cry1Ac as a PIP is the development of resistance in target pests. Practical resistance to Cry1Ac as a PIP in cotton has been reported in populations of bollworm (*Helicoverpa zea*) in the USA [3], and the pink bollworm (*Pectinophora gossypiella*) in India [4]. In *P. gossypiella*, this resistance was associated with alternative splicing which generated aberrant transcripts of cadherin, a receptor for Cry1Ac in that insect [5]. This cadherin that lacks a functional receptor is associated with reduced Cry1Ac binding and resistance [6]. Although the mechanistic description of resistance in *H. zea* remains elusive, mutations in a novel cadherin gene [7] and reduced Cry1Ac processing [8] have been proposed as putative causes.

Despite the existence of cadherin-based resistance to Cry1Ac, the most commonly reported mechanism for practical resistance to Cry protein PIPs in Lepidoptera involves alterations in ATP-binding cassette (ABC) transporter genes serving as receptors for these proteins on the midgut epithelium [9]. Among the ABC transporter superfamily, members of subfamily C2 (*ABCC2*) have been identified as functional receptors for Cry1Ac in *Bombyx mori* [10], *Chloridea* (*Heliothis*) *virescens* [11], *Helicoverpa armigera* [12], *Plutella xylostella* [13,14], *Spodoptera frugiperda* [15] and *S. exigua* [16,17]. Importantly, since these ABCC2 proteins are thought to facilitate oligomerization and insertion of the Cry1Ac protein into the plasma membrane [18], alterations in *ABCC2* genes are linked with resistance to Cry1Ac [9]. The goal of this study was to use the CRISPR/Cas9 gene editing system in testing ABCC2 as a candidate Cry1Ac receptor in *H. zea*. In attaining this goal, we targeted different exons in the *H. zea ABCC2* gene to progressively truncate the ABCC2 transporter protein and then tested the effects on susceptibility to Cry1Ac using diet bioassays.

## 2. Materials and Methods

### 2.1. ABCC2 Transporter Gene Sequence

The publicly available assembled *H. zea* genome [19] and a custom genome assembly from an *H. zea* female from the SIMRU laboratory colony were used to create a searchable database (BlastStation-Local 64 v1.3, TM Software, Inc., Arcadia, CA, USA). The custom genome was sequenced and assembled by a service provider (Hudson Alpha, Huntsville, AL, USA) using the Chromium Genome Solution and Supernova v1.1.5 genome assembly platforms (10× Genomics, Pleasanton, CA, USA). The database was interrogated with the *H. armigera ABCC2* transporter cDNA sequence (GenBank accession number KF479231) using BlastStation-Local 64, and a genomic scaffold containing the *ABCC2* gene was annotated and submitted to GenBank (accession number KY701524). The predicted *ABCC2* gene transcript constructed from the exons identified in the genomic DNA sequence was aligned with ABCC2 mRNA sequences from GenBank to validate the annotation. The translated amino acid sequence was used to identify functional domains of the ABCC2 protein using the Simple Modular Architecture Research Tool (SMART: http://smart.embl-heidelberg.de, accessed on 16 August 2021 [20]). Transmembrane (TMD) and ATPase or nucleotide-binding (NBD) domains were identified and genomic exons corresponding to the TMD and NBD domains were selected as potential CRISPR guide RNA targets. 

### 2.2. Guide RNA Design

All CRISPR RNAs (crRNA) used in the experiments were designed as described in Perera et al., [21] using the tools available at http://crispr.mit.edu/m (accessed on 15 October 2016) [22]. Briefly, target *ABCC2* exon sequences were submitted to the design pipeline and the best crRNA design for each target location was purchased from IDT DNA Inc (Coralville, IA, USA) in the Alt-R™ crRNA format (Table 1). Companion reagents for the Alt-R™ crRNA, universal transactivating CRISPR RNA (Alt-R™ tracrRNA) and a modified Cas9 nuclease containing one N-terminal nuclear localization signal (NLS) and two C-terminal NLS (Cas9-3NLS), were also purchased from IDT DNA Inc.

### 2.3. Insect Colony Maintenance and Egg Collection

All methodology pertaining to *H. zea* colony maintenance, egg collection, crRNA design, and microinjection have been described in detail in Perera et al. [21]. The *H. zea* colony used is routinely maintained at the USDA-ARS Southern Insect Management Research Unit (SIMRU, Stoneville, MS, USA) following the methods described by Gore et al. [23]. Emerging adults were placed in 3.8 L paper containers with 3% sugar solution for 3–4 days to facilitate mating. Females were identified by examining the genitalia and were placed in a 5 × 5 mm wire mesh cage with a piece of wax paper wrapped around it. The wire cage was then placed inside a dark laboratory cabinet for 30 min until egg collection. The wax paper was removed, eggs were dislodged from the wax paper using a fine paint brush, and then mounted on 1 mm wide strips of double-sided tape (3M, St. Paul, MN, USA) attached to 25 × 25 mm plastic coverslips. The eggs were then dehydrated for 15 min by placing in a desiccator containing silica gel and approximately −100 KPa (−30.5 inches of mercury) vacuum before being used for microinjections.

### 2.4. Microinjections

In vitro assembled nucleoprotein complexes containing single guide RNA (sgRNA) and Cas9 were optimized previously in *H. zea* to obtain high rates of genome editing [21]. Using the same protocol, crRNA and tracrRNA were resuspended separately in the nuclease-free duplex buffer (30 mM Hepes pH 7.5, 100 mM potassium acetate) to yield a 100 µM solution. To form functional sgRNA, 1 µL (100 µM) each of reconstituted crRNA and tracrRNA and 8 µL of duplex buffer was mixed to yield a final concentration of 10 µM. When two or more crRNAs were used in a single injection mixture, the tracrRNA was increased to match the sum of all crRNA concentrations to yield a final concentration of 10 µM in a 10 µL solution. This mixture was heated to 95 °C for 5 min in a thermal cycler block and allowed to cool to 25 °C on the bench top. The reconstituted sgRNA mixture (4 µL) was transferred to a new tube where Cas9-3NLS nuclease was added to a concentration equivalent to the sum of the sgRNA concentrations, and the volume of the injection mixture was adjusted to 10 µL with 1 µL of 10× injection buffer (5 mM KCl; 0.1 M sodium phosphate, pH 6.8) and nuclease free water. The final concentration of the sgRNA-Cas9-3NLS nucleoprotein complex in the injection mixture was calculated to be 4 µM. If more than two sgRNAs were used together, the injection mixture was diluted to a final concentration of 2 to 3 µM using 1× injection buffer to prevent clogging of needles due to high concentration of sgRNA-Cas9-3NLS nucleoprotein complexes. Borosilicate microinjection needles prepared with Narishige model PC-10 needle puller (Narishige International USA, Inc., Amityville, NY, USA) and beveled with Sutter Instrument Company (Novato, CA, USA) Model BV-10 beveller were used in initial embryo injections. Microinjection needles were backfilled with approximately 3 µL of the injection mix and the eggs were injected using a Narishige micromanipulator model MMN-333 and an Olympus SZ stereo microscope (Olympus Corporation, Waltham, MA, USA) equipped with a mechanical stage. Each egg was injected with approximately 5 nL of injection mix. Control egg hatch rates were calculated using the eggs processed up to the desiccation stage, but without subjecting them to injection. 

Initial microinjection experiments (Group 1) were performed with guide RNA designed to exons 21, 22 and 24 that targeted the second NBD domain (NBD2) in the ABCC2 protein. After evaluation of the success rate of the first *ABCC2* gene editing effort, two additional rounds of injections were performed by multiplexing different combinations of sgRNA to target the first and second TM domains of the ABCC2 protein. The group 2A sgRNAs targeted exons 3, 8, 13, and 21, while the group 2B sgRNAs targeted exons 13, 19, and 21.

Coverslips containing injected eggs were placed in a 100 mm plastic petri dish layered with a moist filter paper, covered with a lid, sealed with plastic tape, and placed in a plastic box designated as secondary containment. Eggs were incubated at room temperature for approximately 4 hours prior to transferring to a designated incubator set to 26 °C and relative humidity over 75%. Eggs were observed for larval hatch and the neonates were transferred to diet cups containing *Helicoverpa* diet [23]. The total number of eggs injected, and the number of eggs hatched were recorded for each experiment. 

### 2.5. Genotyping and DNA Analysis

Larvae collected from injected embryos (P_0_) were raised to fourth instar and chilled on ice for 30 min prior to obtaining 5–15 µL of hemolymph by cutting the tip of one of the medial (abdominal) prolegs with a pair of micro scissors. Droplets of hemolymph were collected using a pipette and transferred to a tube containing Tissue and Cell Lysis buffer from the MasterPure DNA purification kit (Epicentre Technologies, Madison, WI, USA) supplemented with 20 mg/mL RNAse. Genomic DNA was extracted from hemolymph following the manufacturer’s protocol and re-suspended in 20 µL of 10 mM Tris-HCl. Forward and reverse primers designed for exon 2 (3800Hz_ABCC2_Ex2F) and exon 24 (3809Hz_ABCC2_Ex24R) of the *ABCC2* gene were used to amplify by PCR the *ABCC2* gene using the LongAmp Taq polymerase mix (New England Biolabs, Ipswich, MA, USA) on a PTC-200 DNA Engine (Supplementary data Table S2: Primers). Amplifications were performed with a thermal cycling profile containing 30 second initial denaturation at 95 °C, followed by 10 cycles of 10 sec denaturation at 95 °C, 15 sec annealing at 52 °C, and 8 min 30 sec extension at 65 °C. At the end of the 11th cycle, 25 additional amplification cycles were performed by increasing the 65 °C extension by 10 sec in each successive cycle. The final extension time at the 36th cycle was 12 min for 40 sec. The PCR buffer contained 60 mM Tris-SO_4_, 20 mM ammonium sulfate, 3% glycerol, 0.06% IGEPAL^®^ CA-630 (octylphenoxypolyethoxyethanol), 0.05% Tween^®^ 20, and 2.5 mM MgSO_4_ (pH 9.0). Amplicons from each larva were cleaned by binding to AmPure XP paramagnetic beads (Beckman Coulter, Indianapolis, IN, USA) at DNA:Beads ratio of 1:1.8. Nucleotide sequences of the purified amplicons were obtained by direct sequencing with Sanger dideoxy sequencing using primers designed to flank target sites. Primers used for PCR amplification and sequencing of the *ABCC2* gene are listed in Supplementary data Table S1.

### 2.6. Insect Husbandry

Insects were reared at 28 °C and 14:10 light: dark cycles in environmental chambers (Percival Scientific, Perry, IA, USA). Insects from P_0_ with mutations at the target sites were selected for mating as single pairs with wild type insects of the opposite sex from the SIMRU laboratory colony. Resulting heterozygotes (F_1_) from each single-pair mating were inbred to obtain F_2_ progeny. Fourth instar F_2_ larvae were genotyped by sequencing the DNA extracted from a droplet of hemolymph as described previously and the number of the homozygous mutant, heterozygotes, and homozygous wild type insects were determined. Group matings were set up among homozygous mutants, and as a recovery option in case the homozygous mutant insects did not reproduce, matings were also set between heterozygotes and homozygous mutants, and among heterozygotes. Homozygous insects from each knockout line were inbred for at least four generations, genotyped to verify mutations and used in bioassays to evaluate tolerance to Cry1Ac protein.

### 2.7. Validation of Gene Knockouts by mRNA and Genomic DNA Sequencing

Total RNA extracted from homozygous larvae from each knockout line was treated with DNAse I (NEB) at a concentration of 10 µg/mL for 30 min at 37 °C to remove any carryover genomic DNA. The DNAse was inactivated by heating to 90 °C for 10 min and the cDNA was synthesized using the SuperScript cDNA synthesis kit (Invitrogen, Carlsbad, CA, USA) with an anchored oligo dT primer (5’-GGTAATACGACTCACTATAGGGAGAAGAGGCGAGCACAGAATTAATACGACTTTTTTTTTTTTTTTTTTTTV-3’). Amplification by PCR of the *ABCC2* cDNA was performed using primers 3800 Hz_ABCC2_Ex2F and 3809 Hz_ABCC2_Ex24R. Genomic DNA was amplified using primer pairs as outlined in the Table S1 and purified using the QiaX II gel extraction kit (Qiagen, Germantown, MD, USA). Amplicons were submitted to the USDA-ARS Genomics and Bioinformatics Research Unit (Stoneville, MS, USA) for Sanger dideoxy sequencing. Nucleotide sequence analyses were carried out using the Vector NTI Advance v11.5 suite (Invitrogen). Additional sequencing of cDNA and genomic DNA amplicons from wild type and knockout lines was performed using the Oxford Nanopore Minion system (Oxford Nanopore, Oxford, UK) to obtain long reads, and resulting sequences were assembled using the DNAStar NGen module of DNAStar Lasergene software (DNAStar, Madison, WI, USA). The final curated sequences were submitted to GenBank.

### 2.8. Off-Target Sequence Analysis

The 12-nucleotide seed sequence of each sgRNA (i.e. the 12 nucleotides immediately upstream of PAM) [24] was used to search a local database created from the published *H. zea* genome [19: GCA_002150865.1] using BlastStation-Local64 v1.3 to identify genomic scaffolds with potential off-target sites. Off-target sequences with 0 to 3 mismatches in the 12-nucleotide seed sequence and containing a 5’-NGG-3’ PAM sequence [22,24,25] were selected for further review. Primers were developed to amplify and sequence off-targets with 0 to 2 mismatches in the seed sequence. Amplicons were sequenced on an ABI 3730Xl instrument at the USDA ARS Genomics and Bioinformatics Research Unit (Stoneville, MS, USA). Nucleotide sequences with potential off-target sites were analyzed with the Vector NTI 11.5 Advance suite (Invitrogen).

### 2.9. Bioassays

The *Bacillus thuringiensis* subsp. *kurstaki* HD-73 strain obtained from the Bacillus Genetic Stock Center (Columbus, OH, USA) was used to produce full length Cry1Ac protoxin as described in Little et al. [26]. Bioassays were performed in 128-well plastic trays (B-D International, Franklin Lakes, NJ, USA) with Cry1Ac protein incorporated in meridic diet. Each replicate consisted of 16 bioassay wells containing 0.5 mL of diet supplemented with 1, 10, 100, 500 or 1000 ppm of purified full length Cry1Ac protoxin (approx. 120 kDa). In untreated controls Cry1Ac was replaced with water in diet. A single neonate was placed per well before sealing with a ventilated plastic cover (B-D international, Franklin Lakes, NJ, USA). Trays were incubated at 28 °C for seven days, when stunting and mortality were recorded. Three bioassay replicates were performed for each knockout line, and the SIMRU laboratory colony of *H. zea* was used as a control. Mortality data were evaluated using regression analyses with the PROC PROBIT procedure in SAS v9.4 [27,28] to estimate the concentration that killed 50% of the tested population (LC_50_) and the fiducial limits. Resistance ratios were calculated by dividing the LC_50_ of the knockout and control strains. 

### 2.10. Brush Border Membrane Vesicle (BBMV) Preparation

Midguts of fourth-instar *H. zea* from the SIMRU wild type and the edited SPM-8 and SPM-B1 strains were dissected and used to prepare BBMV using a differential centrifugation method [29], with minor modifications [30]. Isolated BBMV proteins were quantified in a Qubit fluorometer (Invitrogen), and then kept at −80 °C until used (less than two months). Brush border enzyme enrichment in the BBMV preparations was tested measuring specific activity of aminopeptidase-N (APN) using leucine-ρ-nitroanilide as substrate, as described elsewhere [31]. APN activities in the final BBMV preparations were enriched 3 to 5-fold when compared to initial midgut homogenates.

### 2.11. Cry1Ac Radioiodination and Binding Assays

The Cry1Ac protoxin was activated by incubation with bovine trypsin and then purified using anion exchange chromatography, as described previously [32]. Activated Cry1Ac (25 μg) was radiolabeled with 0.5 mCi of NaI^125^ (Perkin Elmer, Boston, MA, USA) using chloramine T, as previously described [33]. The labeled Cry1Ac was purified from free iodine using a PD-10 desalting column (GE Healthcare Life Sciences, Piscataway, NJ, USA) equilibrated in column buffer (20 mM Tris-HCl, 150 mM NaCl, 0.1% BSA, pH 8.6). Fractions containing the radiolabeled Cry1Ac protein based on autoradiography of fractions resolved by SDS-8% PAGE, were pooled and used for binding assays. The specific activity of the labeled Cry1Ac protein was 5.12 µCi/µg, based on input protein.

Binding saturation assays were performed with a constant BBMV protein concentration (0.15 μg/µL) and increasing concentrations of ^125^I-Cry1Ac (from 0.1 to 5 nM) as ligand in a 0.1 mL final reaction volume of binding buffer (PBS, pH 7.4, 0.1% BSA). Reactions were incubated at room temperature for 1 h and then stopped by centrifugation (14,500× *g* for 10 min), and pellets washed with 0.5 mL of ice-cold binding buffer. After the second centrifugation, the radioactivity in the final pellets was measured in a Wizard2 gamma counter (Perkin Elmer). Non-specific binding was determined by including an excess (300-fold the amount of ligand) of unlabeled Cry1Ac in the binding reactions. Specific binding was calculated by subtracting non-specific from the total (in the absence of competitor) binding. Data from two replicated experiments performed in duplicate were fitted to a one binding site model as the best-fitting model in the SigmaPlot v.11.0 software (Systat Software, San Jose, CA, USA) to obtain the apparent dissociation constant (*K_d_*) and concentration of binding sites (*B_max_*).

## 3. Results

### 3.1. Genomic Organization of ABCC2 Transporter Gene in Helicoverpa zea

Searches with the *H. armigera* ABCC2 mRNA sequence (GenBank accession KF479231) identified genomic scaffold KZ118297.1 (188,800 bp in length) in the public *H. zea* genome [19] and scaffold 4812 (190,673 bp) in a custom *H. zea* genome assembly. Gaps in scaffold KZ118297.1 spanned some exons, while the gaps in genomic scaffold 4812 were limited to introns. Therefore, genomic scaffold 4812 was annotated and submitted to GenBank (accession number KY701524). Genomic scaffold 4812 contained genes similar to ribosomal protein L8 of *Bombyx mori* (accession number NP_001037141.1), cryptochrome CRY2-like protein of *H. armigera* (AGA11662.1), golgin subfamily A member 4-like protein of *Amyelois transitella* (XP_013199561.1), decapping and exoribonuclease-like protein of *Papilio machaon* (XP_014360359.1), multidrug resistance-associated protein 4-like of *H. armigera* (XP_021196505.1), SMC domain protein of *Heliothis subflexa* (ADH16742.1), and ABCC3 protein of *H. armigera* (AHL68987.1) The organization of the ABCC2, ABCC3, and SMC domain protein genes was highly conserved between *H. zea* and *H. subflexa* (GenBank accession number GQ332573.1). The *ABCC2* gene of *H. zea* consisted of 25 exons from the putative promoter to the polyadenylation site, spanning nucleotides 97,034 to 110,370 of scaffold 4812. However, only the first 24 exons coded for the ABCC2 transporter polypeptide. Functional domains of the *H. zea* ABCC2 protein predicted using online SMART are shown in Table S2.

### 3.2. Embryo Injections Egg Hatch and Mutation Rates

The initial round of embryo microinjections targeting exons 21, 22, and 24 of the *ABCC2* gene produced a 42.5% hatch rate (74 larvae from 174 injected eggs). Considering that the highly inbred laboratory colony has an average hatch rate of 58.3%, the adjusted hatch rate [34] of injected embryos approximated 72.9%. Of the 74 larvae, 69 pupated and 61 adults emerged. Genetic analysis revealed that 56 adults had mutations in the *ABCC2* gene. Most mutations in the *ABCC2* gene identified in this round of injections were frame-shift deletions ranging from 1 to 19 bp at the target site in exon 21. Of the single pair matings with injected P_0_ adults, only 18 produced progeny. Screening of inbred F_2_ progeny from resulting families identified insects from several families homozygous for *ABCC2* gene mutations in exons 21 and 22. One family (SPM8) with a 7 bp frame shift deletion (Supplementary, Figure S1) that truncated the NBD2 was selected to establish a colony for further evaluation. SPM-8 also had a 6 bp insertion at the sgRNA target site in exon 22. Another mutant family, SPM16, with a 426 bp deletion in the genomic DNA starting at the sgRNA target site in exon 21 that eliminated the exon 21 downstream of the target site along with a part of intron 21 and a 4 bp insertion at the exon 22 target site was also selected for further evaluation (Supplementary, Figure S2).

**Figure 1:**
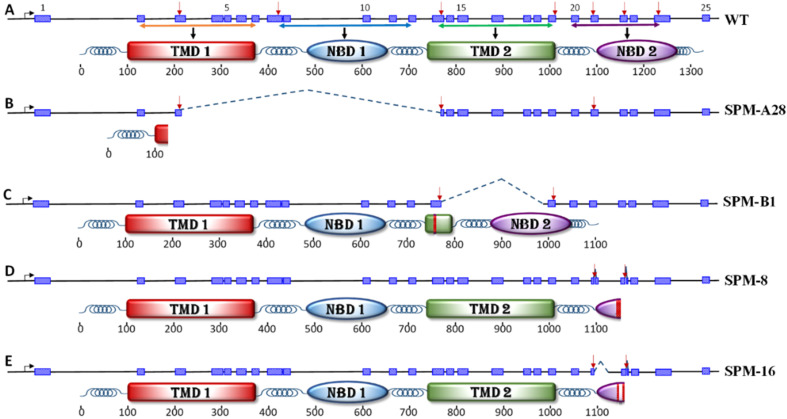
Organization of *Helicoverpa zea* ATP binding cassette transporter subfamily C2 (*ABCC2*) transporter gene and CRISPR/Cas9 mediated deletions generated and tested in this study. Exons and introns, spaced to scale, are shown by blue hatched rectangles and solid black lines, respectively. Numbers below the predicted polypeptide represent amino acids. (**A**). Wild type *ABCC2* gene containing 25 exons and predicted polypeptide with transmembrane (TMD) and nucleotide-binding (NBD) domains. Exons 1, 5, 10, 15, 20, and 25 are numbered above the gene diagram. Approximate location of the promoter and exons coding for TMD 1, TMD 2, NBD 1, and NBD 2 are shown by horizontal double arrows. Locations for target sites in exons 3, 8, 13, 19, 21, 22, and 24 for which single guide RNA (sgRNA) were designed are shown with red down arrows. (**B**)**.** Knockout line SPM-A28C generated by sgRNA targeting exons 3 and 13 which truncated the ABCC2 protein at amino acid 127. (**C**). Knockout line SPM-B1 with a deletion from exon 13 to 19 recovered from the experiment targeting exons 13, 19, and 21 in a polypeptide that had a deletion of 255 amino acids at the beginning of the TMD 2 (starting at amino acid 760). Alternative splicing using a splice donor (GT) site in exon 13 to exon 19 restored the NBD2 domain of the ABCC2 protein. (**D**,**E**). Knockouts generated by sgRNA targeting exons 21, 22, and 24. In SPM-8 (**D**), a 7 bp deletion in exon 21 (from 93,539 to 93,545 bp in genomic scaffold) truncated the polypeptide at amino acid 1122 and added eight random amino acids, inactivating NBD2. In knockout line SPM-16 (**E**), a deletion of 426 bp from 93,540 to 93,965 (exon 21 to intron 21) created an open reading frame and extended the truncated NBD2 polypeptide sequence with 34 random amino acids. In this knockout line there was an additional 4 bp insertion at the exon 22 sgRNA target site (position 94,196 bp of the genomic scaffold) that did not affect the outcome due to truncation of the protein by the deletion in exon 21. Red arrows in (**B**–**E**) indicate the position of sgRNA that caused the mutation in each line.

**Figure 2:**
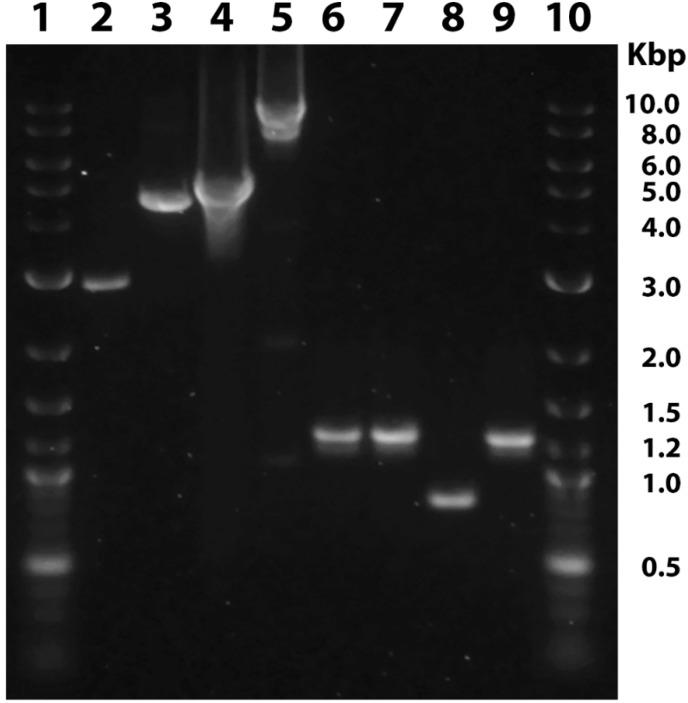
Agarose gel electrophoresis of amplicons of ATP binding cassette transporter subfamily C2 (ABCC2) knockout lines and wild type insects of *H. zea*. Lanes 2, 4, 6, and 8: SPM-8, SPM-16, SPM-B1, and SPM-A28C, respectively. Lanes 3, 5, 7, and 9: amplicons from wild type insects. Lane 1 and 10: DNA ladder with molecular weights of the main bands (in Kbp) marked.

Subsequent embryo injections targeting TMD1 and TMD2 with multiplexed sgRNAs of groups 2A and 2B yielded 22.8% and 18.5% hatch rates, respectively, with a combined average hatch rate of 20.7%. The hatch rate of the uninjected control eggs collected from the same batch of moths was 40.6% (69 out of 170). The corrected average [34] hatch rate for this set of injected eggs was 50.9%. A total of 10 larvae from injected embryos (P_0_) died during pupation due to inability to complete transformation. In addition, 20 pupae also failed to develop into adults. Of the 82 adults that emerged, seven from each sex had severe wing and body deformities that precluded successful mating. Single pair matings of 37 females and 31 males of the P_0_ with wild type insects from the SIMRU laboratory colony gave rise to 30 and 20 F_1_ families, respectively. The results of embryo injections are summarized in Table S3. After evaluating all nucleotide sequence data, families SPM-A28C and SPM-B1 were selected to represent deletions of TMD1 and TMD2 domains, respectively. A large deletion of 4686 bp spanning from sgRNA target sites in exon 3 to exon 13 was identified in the knockout line SPM-A28C. This knockout line also had a 20 bp insertion at the exon 21 sgRNA target site (Supplementary, Figure S3). In knockout line SPM-B1, a deletion of 1896 bp from the sgRNA target site in exons 13 to intron 17 and a 10 bp deletion at the exon 21 target site were identified (Supplementary, Figure S4). Deletions and insertions caused by CRISPR/Cas9 at target sites of the knockout lines used in bioassays are shown in Figure 1. Sizes of genomic DNA amplicon from knockout lines and wild type insects are shown in Figure 2. True breeding lines for conducting bioassays were established by inbreeding the selected knockout lines.

Evaluation of cDNA sequences generated from mRNA isolated from *ABCC2* gene knockout lines identified proteins that lack functional NBD2 (SPM8 and SPM-16), TMD2 (SPM-B1), or complete knockout of the ABCC2 protein (SPM-A28C). Alignment of cDNA sequences obtained from knockout lines with the reference full-length ABCC2 mRNA (KM360184) and putative amino acid sequences translated from the cDNA sequences are shown in Figures S5 and S6, respectively. Deletion of 7 bp in exon 21 in SPM-8 truncated the ABCC2 protein at amino acid 1128, eliminating most of the NBD2 and adding 17 random amino acids to the end of the truncated protein (Supplementary, Figure S6). Deletion of the latter half of exon 21 and a part of intron 21 in SPM-16 eliminated the splice donor site (GT) at the end of exon 21, leading to splicing of 49 nucleotides of intron 21 to exon 22. This deletion and the mis-splicing combination deleted 27 amino acids from the NBD2 of the ABCC2 protein (1127 to 1134 aa) while inserting 15 random amino acids into the protein. After the insertion of random amino acids, the reading frame of the exon 22 was restored but the 4 bp insertion at the exon 22 sgRNA target site caused a frameshift that inserted 19 random amino acids at the carboxy terminal end of the truncated ABCC2 protein, which rendered the NBD2 non-functional. The deletion in SMP-B1 between the sgRNA target site in exon 13 and intron 18 apparently led to use of an alternative splice donor site upstream of the exon 13 deletion to join 236 nucleotides of exon 13 to exon 19. This deletion of a part of exon 13 and all of exons 14 to 18 eliminated 255 amino acids forming the TMD2 (from 760 to 1013 aa) of the ABCC2 protein. The deletion of 10 bp at the exon 19 sgRNA target site also led to an alternative splicing event that eliminated 19 amino acids while inserting 10 random amino acids for a net deletion of nine amino acids. However, the amino acid sequence was restored to wild type sequence after amino acid position 1077 (Supplementary, Figure S6). In knockout line SPM-A28C, the ABCC2 protein was truncated after amino acid 127, leaving only the first transmembrane helix of the TMD1 intact (Supplementary, Figure S6 and Table S2). Nucleotide sequences from the genomic DNA and mRNA of the knockout lines were submitted to GenBank under accession numbers MZ913261 to MZ913265 and MZ954929 to MZ954932, respectively.

### 3.3. Off-Target Analysis

Searching the genome of *H. zea* with sgRNA sequences identified one potential off-target site for exon 13 sgRNA in scaffold KZ118710 and two and one potential off-target site for exon 19 sgRNA in genomic scaffolds KZ117297 and KZ117617, respectively (Table 2). No off-targets matching the search criteria were identified for the remaining sgRNA sequences. The exon 13 sgRNA was used for embryo injections generating lines SPM-A28C and SPM-B1, and the exon 19 sgRNA was used in generating knockout line SPM-B1. Therefore, genomic DNA from both SPM-A28C and SPM-B1 was amplified and sequenced using primers developed for the off-target sites in scaffold KZ118710 and for SPM-B1 also for off-target sites in scaffolds KZ117297 and KZ117617 (Supplementary, Table S1). Amplicons from wild type insects were used as the reference. Alignments of nucleotide sequences obtained from direct sequencing of amplicons from knockout lines SPM-A28C, SPM-B1, and wild type with reference genomic scaffold sequences indicated lack of mutations in the off-target sites evaluated (Supplementary, Figure S7). 

### 3.4. Bioassays

Bioassays conducted with purified Cry1Ac protoxin indicated different levels of tolerance associated to distinct mutated ABCC2 domains (Table 3). SPM-8, which had the second ATPase domain (NBD2) inactivated, showed the lowest tolerance (RR = 7.3). The knockout line SPM-16 with NBD2 deletion and in-frame insertion was slightly more tolerant to Cry1Ac, yet the LC_50_ fiducial limits overlapped with SPM-8, supporting no significant differences. The SPM-B1 line, with both TMD 2 and NBD2 knocked out, had the highest resistance ratio (RR = 39.8). The LC_50_ fiducial limits for SPM-B1 did not overlap with those of SPM-8 but overlapped with the fiducial limits of SPM-16. Knockout line SPM-A28C, in which the ABCC2 transporter was almost completely knocked out, had an LC_50_ that was not significantly different from SPM-8 or SPM-16.

### 3.5. Saturation Cry1Ac Binding Assays

**Figure 3:**
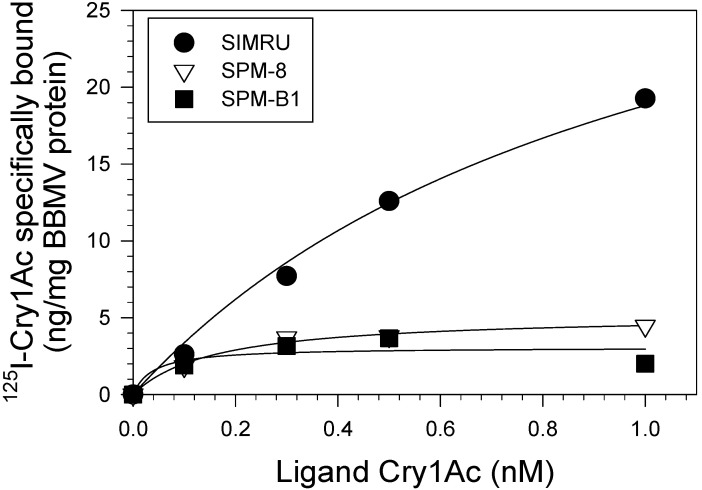
Saturation *Cry1Ac*-binding assays with midgut brush border membrane vesicles (BBMV) from larvae of wild type (SIMRU) and gene-edited (SPM-8 and SPM-B1) strains of *Helicoverpa zea*. Data points shown are the specific binding estimated from total and non-specific binding (as detailed in Materials and Methods) from two independent experiments each performed in duplicate. Curves are the result from non-linear regression fitting of the data to a one-site binding model as the best fit.

Based on results from bioassays, we selected the SPM-8 and SPM-B1 lines, as having the lowest and highest susceptibility among edited strains, for *Cry1Ac*-binding assays. In saturation assays with radiolabeled *Cry1Ac* and BBMV from SIMRU we detected high-affinity saturable binding (Figure 3 and Table 4). In contrast, specific Cry1Ac binding was dramatically reduced in BBMV from SPM-8 and SPM-B1 strains when compared to SIMRU (Figure 3). Estimation of binding parameters for SPM-8 and SPM-B1 detected a ≥10-fold reduction in the concentration of binding sites (*B_max_*) relative to SIMRU BBMV (Table 4). We were unable to obtain accurate estimates of affinity (*K_d_*) for SPM-8 and SPM-B1 from the dataset (*p* in both cases >0.05).

## 4. Discussion

Members of the ABC transporter family C2 (ABCC2) are functional receptors for Cry1 proteins in lepidopteran larvae [35]. Mutations in *ABCC2* genes have been genetically linked with high levels of resistance to Cry1Ac in the Heliothinae subfamily, including *Heliothis virescens* [11] and *H. armigera* [36]. However, not much is known about the role of ABCC2 as Cry1Ac receptor in the closely related *H. zea*. In addressing this knowledge gap, we obtained progressive deletions and a complete *ABCC2* gene knockout using the CRISPR/Cas9 editing system, and then tested the putative Cry1Ac functional receptor role of ABCC2 protein in *H. zea.* Bioassays testing the edited *H. zea* lines detected moderately reduced susceptibility compared to the parental (unedited) line. Similarly, knockout of *ABCC2* gene in the closely related species *H. armigera* resulted in 3.8-fold reduced susceptibility to Cry1Ac toxin, and high levels of resistance (>1500-fold) were only observed with additional knockout of ABCC3 [37]. Akin results were observed in *P. xylostella* strains with natural mutations or with CRISPR-edited single and double *ABCC2* and *ABCC3* knockouts [38]. More recently, higher levels of resistance (112-fold) to Cry1Ac protoxin were reported after *ABCC2* gene knockout in *H. armigera*, and even higher levels of resistance (816-fold) were detected with an additional knockout of cadherin [39]. In contrast, knockout of *ABCC2* gene in *P. xylostella* resulted in high resistance (724-fold) to Cry1Ac protoxin [14], suggesting that ABCC2 has distinct relevance to Cry1Ac intoxication depending on the species. In addition, we found that there was no association between the size of the *ABCC2* deletion and susceptibility to Cry1Ac. For example, knockout of the NBD2 in lines SPM-8 and SPM-16 resulted in only marginal tolerance to Cry1Ac, which was not different from tolerance in the complete *ABCC2* knockout line SPM-A28C. The SPM-B1 line, which had TM2 and NBD2 deleted, showed the highest tolerance to Cry1Ac (39.8-fold) compared to the wild type, but still was not significantly different from tolerance in SPM-16.

Previous reports identified residues in loop 1 of TMD1 as critical for Cry1Ac binding to ABCC2 in *Spodoptera* spp., and these residues are conserved in ABCC2 of *H. zea*. [40,41]. The only knockout line tested in the present study in which these residues would not be present in ABCC2 was the almost full-length knockout SPM-A28C. However, susceptibility to Cry1Ac in SPM-A28C was not different from susceptibility in the SPM-8 strain, which was edited to eliminate the NBD2. This observation suggests that ABCC2 in *H. zea* must be functionally active and that simple interactions with the extracellular loops of TMDs are not sufficient to support Cry1Ac toxicity. This hypothesis is not supported by results from mutant ABCC2 proteins lacking NBDs still functioning as Cry receptors [16,42]. Alternatively, it is also possible that truncated ABCC2 is not produced and present in the BBMV, as was reported for *S. frugiperda* with a mutational disruption of the *sf*ABCC2 gene [43]. In support of this hypothesis, binding of Cry1Ac was dramatically reduced in the tested *ABCC2* gene knockout strains, independently of the domain targeted or their level of susceptibility to Cry1Ac. This observation also suggests that the remaining low levels of Cry1Ac binding to alternative receptors other than ABCC2 in *H. zea* BBMV are still conducive to toxicity. An alternative previously reported Cry1Ac receptor in *H. zea* BBMV is the alkaline phosphatase ALP2 [44]. Since mutations in a novel cadherin gene are associated with field-evolved resistance of *H. zea* to Cry1Ac, this could also be an alternative receptor [7]. In fact, cadherin knockout in *H. armigera* resulted in higher resistance to Cry1Ac compared to *ABCC2* knockout [37,39], which may be indicative of their relative importance for Cry1Ac toxicity in *Helicoverpa* spp.

In summary, we used synthetic sgRNAs and recombinant Cas9 nuclease to assemble nucleoproteins that were multiplexed to target multiple protospacer sites in the *H. zea ABCC2* gene to efficiently recover desired knockout lines. Mutations that truncated the ABCC2 at TMD1, TMD2, or NBD2 resulted in low levels of tolerance to Cry1Ac, suggesting that by itself the ABCC2 protein is not a critical Cry1Ac receptor. Future work will focus on examining alternative Cry1Ac receptors and their putative interactions with ABCC2 during Cry1Ac intoxication, as described for cadherin in *H. armigera* [45].

## Figures and Tables

**Table 1:** CRISPR RNA (crRNA) sequence (red) and protospacer adjacent motif (blue) used for targeting different exons in the *Helicoverpa zea* ATP binding cassette transporter subfamily C2 (*ABCC2*) gene (GenBank KY701524). Target sites in the coding DNA strand are denoted by a plus (+) sign, and those on the reverse strand are denoted by a minus (−) sign.

crRNA Name	crRNA Sequence	Strand	Target
Hz_Ex3	GCTACTGTCGTACTGGTCGG TGG	+	Exon 3
Hz_Ex8	TTAACAAAGTAAGCGCATCG TGG	+	Exon 8
Hz_Ex13	CTGCCGACTATTGGTTGAGT TGG	−	Exon 13
Hz_Ex19	TTGCTCCATATTGGTCTCCG TGG	−	Exon 19
Hz_Ex21	CGGCAAGTCATCGCTCATCG CGG	+	Exon 21
Hz_Ex22	ACAGCGACGACGATATTTGG AGG	+	Exon 22
Hz_Ex24	GGTCATGGACCAGGGCGAAG TGG	+	Exon 24

**Table 2:** Potential off-target sequences for single guide RNA (sgRNA) targeting exon 13 and 19 in the genome of *Helicoverpa zea*. Scaffold number, nucleotide position, off-target sequence, and the strand of DNA are given. Nucleotides of the protospacer adjacent motif are shown in red bold text and 12-nucleotide seed sequences are shown in bold Italics. Nucleotides identical to each sgRNA are shown by a period.

sgRNA/Scaffold	Position	Sequence	Strand
sgRNA Exon 13		ACTCAACC***AATAGTCGGCAG*** **TGG**	
KZ118710.1	289298-289320	CAAT...T............ **TGG**	+
sgRNA Ex19		TTGCTCCA***TATTGGTCTCCG*** TGG	
KZ117297.1	190397-190417	G..TGATT***C***........... TGG	−
	204522-204544	GCTAGTA..***T***...***T***...... TGG	-
KZ117617.1	70340-70362	C.TACT.T.***A***.......... TGG	-

**Table 3:** Bioassay data for control (SIMRU) and ATP binding cassette transporter subfamily C2 (ABCC2) knockout lines of *Helicoverpa zea* to estimate the concentrations Cry1Ac that killed 50 and 90% larvae (LC_50_ and LC_90_, respectively) and their fiducial limits (Lower-Upper). Slope of the regression line, standard error of slope, Chi-square value of the slope and the *p*-value are also presented. Significantly different LC_50_ values (based on non-overlapping fiducial limits) are denoted by different symbols (^†^, ^‡^, and ^§^). Cry1Ac concentrations (µg/mL) used in bioassays: ^a^ 0, 1, 10, 50,100; ^b^ 0, 1, 10, 100, 500; ^c^ 0, 1, 10, 100, 1000; ^d^ 0, 1, 10, 100; ^e^ 0, 0.1, 1, 10, 100. RR = LC_50_ ratio between edited and control strain.

Insect Line	ABCC2 Domain Knockout	LC_50_ µg/mL (Lower-Upper)	RR	LC_90_ µg/mL (Lower-Upper)	Slope ± SE	Χ^2^ Slope
SPM-A28C ^a^	Complete	21.87 ^†^ (13.92–33.77)	9.2	251.65 (141.04–576.35)	1.22 ± 0.14	74.00 *p* < 0.0001
SPM-B1 ^b^	TM2 to NBD2	94.32 ^‡^ (65.75–137.50)	39.8	455.69 (282.81–946.91)	1.87 ± 0.25	54.58 *p* < 0.0001
SPM-16 ^c^	NBD2 (insertion)	42.58 ^†^ (24.71–74.62)	18.0	1118.00 (491.32–3876.00)	0.90 ± 0.11	65.79 *p* < 0.0001
SPM-8 ^d^	NBD2	17.35 ^†,‡^ (10.82–29.13)	7.3	355.42 (156.54–1357.00)	0.98 ± 0.13	52.79 *p* < 0.0001
Control ^e^	None	2.37 ^§^ (1.75–3.20)	1	22.31 (14.85–37.31)	1.32 ± 0.10	162.63 *p* < 0.0001

**Table 4:** Binding parameters from saturation *Cry1Ac* binding assays and goodness of fit criteria for wild type (SIMRU) and gene edited (SPM-8 and SPM-B1) strains of *Helicoverpa zea*. *K_d_* = dissociation constant (in nM units); *B_max_* = concentration of receptors (in ng/mg BBMV protein units); SE = standard error; *P* = statistic for the parameter estimate (significant estimates (*p* < 0.05) are underlined); *R^2^* = coefficient of determination for the regression model used for fitting (one binding site model).

Strain	*K_d_* ± SE	*P*	*B_max_* ± SE	*P*	*R^2^*
SIMRU	1.55 ± 0.37	0.0002	49.61 ± 8.08	0.0024	0.9978
SPM-8	0.16 ± 0.09	0.1094	5.20 ± 0.84	0.0002	0.9883
SPM-B1	0.04 ± 0.05	0.4244	3.07 ± 0.50	0.0002	0.7669

## Data Availability

All pertinent nucleotide sequence data have been deposited in public databases and/or have been reported in the text, figures, or tables.

## Supplementary Materials

The following are available online at https://www.mdpi.com/article/10.3390/genes12101522/s1, Figure S1: Alignment of *Helicoverpa zea* ATP binding cassette transporter subfamily C2 (*ABCC2*) genome sequence from 107,087 to 108,334 bp and amplicon sequence from knockout line SPM-8 with 7 bp deletion at the exon 21 sgRNA target site and 6 bp net insertion at the exon 22 sgRNA target site. Figure S2: Alignment of *Helicoverpa zea* ATP binding cassette transporter subfamily C2 (*ABCC2*) genome sequence from 107,089 to 108,333 bp and amplicon sequence from knockout line SPM-16 with a deletion of 426 bp and an insertion of four bp at the sgRNA target sites in exon 21 and 22, respectively. Figure S3: Alignment of *Helicoverpa zea* ATP binding cassette transporter subfamily C2 (*ABCC2*) reference genome sequence from 99,062 to 108,844 bp and amplicon sequences from wild type SIMRU and knockout line SPM-A28C. Figure S4: Alignment of *Helicoverpa zea* ATP binding cassette transporter subfamily C2 (*ABCC2*) reference genome sequence from 104,174 to 108,822 bp and amplicon sequence from the knockout line SPM-B1 with 1876 bp and 10 bp deletions at the sgRNA target sites of exon 13 and exon 19, respectively. Figure S5: Alignment of partial mRNA sequences of *Helicoverpa zea* ATP binding cassette transporter subfamily C2 (*ABCC2*) from knockout lines SPM-8, SPM-16, SPM-B1, and SPM-A28C with H. zea ABCC2 mRNA sequence KM360184 from GenBank. Figure S6: Alignment of putative amino acid sequences predicted from partial mRNA sequences of *Helicoverpa zea* ATP binding cassette transporter subfamily C2 (*ABCC2*) gene knockout lines SPM-8, SPM-16, SPM-B1, and SPM-A28C with the ABCC2 amino acid sequence AKH49600 from GenBank. Figure S7: Alignment of nucleotide sequences from potential off-target sites for exon 13 and exon 19 sgRNA in *Helicoverpa zea* ATP binding cassette transporter subfamily C2 (*ABCC2*) knockout lines SPM-A28C and SPM-B1. Table S1: Primer names and sequences used for PCR amplification and nucleotide sequencing of *Helicoverpa zea* ATP binding cassette transporter subfamily C2 (*ABCC2*) gene and off-target sites. Table S2: Functional domains predicted in the ATP binding cassette transporter subfamily C2 (ABCC2) protein of *Helicoverpa zea* predicted using online Simple Modular Architecture Research Tool (SMART: http://smart.embl-heidelberg.de accessed on 16 August 2021). Table S3: The number of eggs injected, percent larvae hatched, the number of males and females, and the total adults recovered from three rounds of embryo injections with sgRNA/Cas9 nucleoprotein complexes targeting ATP binding cassette transporter subfamily C2 (*ABCC2*) gene in *Helicoverpa zea*.
