# Supplementary material for: Genetic Knockouts Indicate That the ABCC2 Protein in the Bollworm Helicoverpa zea Is Not a Major Receptor for the Cry1Ac Insecticidal Protein
Source: Genes (Basel). 2021 Sep 28;12(10):1522. doi: 10.3390/genes12101522 (PMC8535714; doi:10.3390/genes12101522)
Supplement: Supplementary file 1 [file genes-12-01522-s001.zip › genes-1385674-supplementary.pdf]

```
            107087
Hz_ABCC2    GGAAGACCCGCCGGTGCTGAAGGACTTAAACTTTGAAATTCAAAGCGGCTGGAAGGTAAGAGACCTAATAATGTATTTGA
SPM-8    (1)GGAAGACCCGCCGGTGCTGAAGGACTTAAACTTTGAAATTCAAAGCGGCTGGAAGGTAAGAGACCTAATAATGTATTTGA
            107167
Hz_ABCC2    TTAAAATAAAAGCAATAAAGATATACTTGCCGTGCTACCCTAAACTACCCGTGTTCCATGTATTATTCAGATAAATTCCA
SPM-8 (81)  TTAAAATAAAAGCAATAAAGATATACTTGCCGTGCTACCCTAAACTACCCGTGTTCCATGTATTATTCAGATAAATTCCA
            107247
Hz_ABCC2    TCCAAAGGTTGTCTAGAAGAAATCGCTGTTTGGCGATAAGACGGCCAATTATACTGTTCTTTGTGTCATATGATTATCAA
SPM-8 (161) TCCAAAGGTTGTCTAGAAGAAATCGCTGTTTGGCGATAAGACGGCCAATTATACTGTTCTTTGTGTCATATGATTATCAA
            107327
Hz_ABCC2    GATGATATGATGAGAAGATTTATAAAGAATAATCTATCTATGTATTTGCTTACTTAATGTAATGAATGTTGTAGGTTGGA
SPM-8 (241) GATGATATGATGAGAAGATTTATAAAGAATAATCTATCTATGTATTTGCTTACTTAATGTAATGAATGTTGTAGGTTGGA
            107407
Hz_ABCC2    GTTGTAGGCAGAACAGGAGCCGGCAAGTCATCGCTCATCGCGGCTTTGTTCCGGCTTAGTGACATAAGCGGCAGCATCAA
SPM-8 (321) GTTGTAGGCAGAACAGGAGCCGGCAAGTCATCGC-------GGCTTTGTTCCGGCTTAGTGACATAAGCGGCAGCATCAA
            107487
Hz_ABCC2    AATTGACGGTGTGGACACCGAAGGATTAGCCAAAAAGGTATCGATGTTAAAAAATACATTCTTTCTAAAAGACTTAGTGA
SPM-8 (394) AATTGACGGTGTGGACACCGAAGGATTAGCCAAAAAGGTATCGATGTTAAAAAATACATTCTTTCTAAAAGACTTAGTGA
            107567
Hz_ABCC2    AGTATTATTTATTGTTTAGTTAAATTACTAATAGAATTTGTGGATTTGTTGAAAATATATTAACGGCACGAGCTCGTGGT
SPM-8 (473) AGTATTATTTATTGTTTAGTTAAATTACTAATAGAATTTGTGGATTTGTTGAAAATATATTAACGGCACGAGCTCGTGGT
            107647
Hz_ABCC2    TTCCAGCACCCGGATAAAAATATCCTTTTTATTGCAATTTTATTTGCACTCTCTTTTATGAACTCTCTTATGTACTCACC
SPM-8 (553) TTCCAGCACCCGGATAAAAATATCCTTTTTATTGCAATTTTATTTGCACTCTCTTTTATGAACTCTCTTATGTACTCACC
            107727
Hz_ABCC2    TTAAGTACTCTCTTATGTAAGATCTCATGTACTTCCCTTATGTACTCTCTTACGTACCCTCTTATGTGCTCTCTTATGTA
SPM-8 (633) TTAAGTACTCTCTTATGTAAGATCTCATGTACTTCCCTTATGTACTCTCTTATGTACCCTCTTATGTGCTCTCTTATGTA
            107807
Hz_ABCC2    CTCCCCTTATGTACTCCTCTTCTGTACTCCTCTTATACAAAGTGTGTCGTACCTAATCACATGAAATTAAATACGTTGAT
SPM-8 (713) CTCCCCTTATGTACTCCTCTTCTGTACTCCTCTTATACAAAGTGTGTCGTACCTAATCACATGAAATTAAATACGTTGAT
            107887
Hz_ABCC2    ATACTGGTTAATACATTTGTAGGAAAAGTAAGAGAATCTTATGTCGTCAATTTTTTTTTTATTTAGAACATTGATTCAGCG
SPM-8 (793) ATACTGGTTAATACATTTGTAGGAAAAGTAAGAGAATCTTATGTCGTCAATTTTTTTTTTATT-AGAAAATTGATTCAGCG
            107967
Hz_ABCC2    ATAACTTTTTACATTCGCAGACTTTGAGATCGAAAATATCAATTATTCCACAAGAGCCGGTGCTGTTCTCGGCTACTCTG
SPM-8 (872) ATAACTTTTTACATTCGCAGACTTTGAGATCGAAAATATCAATTATTCCACAAGAGCCGGTGCTGTTCTCGGCTACTCTG
            108047
Hz_ABCC2    CGATACAATTTGGACCCGTTCGACGATTACAGCGACGACGATAT------TTGGAGGGCGTTGGAACAGGTACGCTTAAT
SPM-8 (952) CGATACAATTTGGACCCGTTCGACGATTACAGCGACGACGATAGAAGGCGTTGGAGGGCGTTGGAACAGGTACGCTTAAT
            108121
Hz_ABCC2    TAAATTACTTCTCAACAGACCCTCGGCACTCGAACTAATCTAACTAATGAATGAATCTATGATTTTAGGTGGAATTAAAA
SPM-8(1032) TAAATTACTTCTTAACAGACCCTCGGCACTCGAACTAATCTAACTAATGAATGAATCTATGATTTTAGGTGGAATTAAAA
            108201
Hz_ABCC2    GAAGGAATACCGGCTTTAGACTTTAAGGTCGCTGAAGGTGGTACTAACTTCTCTATGGGACAACGTCAGTTGGTTTGCTT
SPM-8(1112) GAAGGAATACCGGCTTTAGACTTTAAGGTCGCTGAAGGTGGTACTAACTTCTCTATGGGACAACGTCAGTTGGTTTGCTT
            108281
Hz_ABCC2    GGCTCGTGCCATTCTACGCTCTAACAAAATACTCATCATGGACGAAGCTACCG
SPM-8(1192) GGCTCGTGCCATTCTACGCTCTAACAAAATACTCATCATGGACGAAGCTACCG
```

Figure S1. Alignment of *Helicoverpa zea* ATP binding cassette transporter subfamily C2 (*ABCC2*) genome sequence from 107,087 to 108334 bp and amplicon sequence from knockout line SPM-8 with 7 bp deletion at the exon 21 sgRNA target site and 6 bp net insertion at the exon 22 sgRNA target site. Identical nucleotides are shown in white text with black background and alignment gaps are indicated by hyphens. Deletions and insertions caused by CRISPR/Cas9 editing at sgRNA target sites are marked with red bold text and nucleotide polymorphisms between the reference genomic scaffold (Accession: KY701524) sequence and amplicon sequences are shown in dark gray text.

```
            107089
Hz_ABCC2    AAGACCCGCCGGTGCTGAAGGACTTAAACTTTGAAATTCAAAGCGGCTGGAAGGTAAGAGACCTAATAATGTATTTGATT
```

```
SPM-16   (1) AAGACCCGCCGGTGCTGAAGGACTTAAACTTTGAAATTCAAAGCGGCTGGAAGGTAAGAGACCTAATAATGTATTTGATT
             107169
Hz_ABCC2     AAAATAAAAGCAATAAAGATATACTTGCCGTGCTACCCTAAACTACCCGTGTTCCATGTATTATTCAGATAAATTCCATC
SPM-16  (81) AAAATAAAAGCAATAAAGATATACTTGCCGTGCTACCCTAAACTACCCGTGTTCCATGTATTATTCAGATAAATTCCATC
             107249
Hz_ABCC2     CAAAGGTTGTCTAGAAGAAATCGCTGTTTGGCGATAAGACGGCCAATTATACTGTTCTTTGTGTCATATGATTATCAAGA
SPM-16 (161) CAAAGGTTGTCTAGAAGAAATCGCTGTTTGGCGATAAGACGGCCAATTATACTGTTCTTTGTGTCATATGATTATCAAGA
             107329                                                                          11612
Hz_ABCC2     TGATATGATGAGAAGATTTATAAAGAATAATCTATCTATGTATTTGCTTACTTAATGTAATGAATGTTGTAGGTTGGAGT
SPM-16 (241) TGATATGATGAGAAGATTTATAAAGAATAATCTATCTATGTATTTGCTTACTTAATGTAATGAATGTTGTAGGTTGGAGT
             107409
Hz_ABCC2     TGTAGGCAGAACAGGAGCCGGCAAGTCATCGCTCATCGCGGCTTTGTTCCGGCTTAGTGACATAAGCGGCAGCATCAAAA
SPM-16 (321) TGTAGGCAGAACAGGAGCCGGCAAGTCATC--------------------------------------------------
             107489
Hz_ABCC2     TTGACGGTGTGGACACCGAAGGATTAGCCAAAAAGGTATCGATGTTAAAAAATACATTCTTTCTAAAAGACTTAGTGAAG
SPM-16 (351) --------------------------------------------------------------------------------
             107569
Hz_ABCC2     TATTATTTATTGTTTAGTTAAATTACTAATAGAATTTGTGGATTTGTTGAAAATATATTAACGGCACGAGCTCGTGGTTT
SPM-16 (351) --------------------------------------------------------------------------------
             107649
Hz_ABCC2     CCAGCACCCGGATAAAAATATCCTTTTTATTGCAATTTTATTTGCACTCTCTTTTATGAACTCTCTTATGTACTCACCTT
SPM-16 (351) --------------------------------------------------------------------------------
             107729
Hz_ABCC2     AAGTACTCTCTTATGTAAGATCTCATGTACTTCCCTTATGTACTCTCTTACGTACCCTCTTATGTGCTCTCTTATGTACT
SPM-16 (351) --------------------------------------------------------------------------------
             109809
Hz_ABCC2     CCCCTTATGTACTCCTCTTCTGTACTCCTCTTATACAAAGTGTGTCGTACCTAATCACATGAAATTAAATACGTTGATAT
SPM-16 (351) --------------------------------------------------------ACATGAAATTAAATACGTTGATAT
             107889
Hz_ABCC2     ACTGGTTAATACATTTGTAGGAAAAGTAAGAGAATCTTATGTCGTCAATTTTTTTTTTATTTAGAACATTGATTCAGCGAT
SPM-16 (375) ACTGGTTAATACATTTGTAGGAAAAGTAAGAGAATCTTATGTCGTCAATTTTTTTTTTATTTAGAAAATTGATTCAGCGAT
             107969
Hz_ABCC2     AACTTTTTACATTCGCAGACTTTGAGATCGAAAATATCAATTATTCCACAAGAGCCGGTGCTGTTCTCGGCTACTCTGCG
SPM-16 (455) AACTTTTTACATTCGCAGACTTTGAGATCGAAAATATCAATTATTCCACAAGAGCCGGTGCTGTTCTCGGCTACTCTGCG
             108045
Hz_ABCC2     ATACAATTTGGACCCGTTCGACGATTACAGCGACGACGATATTT----GGAGGGCGTTGGAACAGGTACGCTTAATTAAA
SPM-16 (535) ATACAATTTGGACCCGTTCGACGATTACAGCGACGACGATATTTATATGGAGGGCGTTGGAACAGGTACGCTTAATTAAA
             108125
Hz_ABCC2     TTACTTCTCAACAGACCCTCGGCACTCGAACTAATCTAACTAATGAATGAATCTATGATTTTAGGTGGAATTAAAAGAAG
SPM-16 (615) TTACTTCTTAACAGACCCTCGGCACTCGAACTAATCTAACTAATGAATGAATCTATGATTT-AGGTGGAATTAAAAGAAG
             108204
Hz_ABCC2     GAATACCGGCTTTAGACTTTAAGGTCGCTGAAGGTGGTACTAACTTCTCTATGGGACAACGTCAGTTGGTTTGCTTGGCT
SPM-16 (694) GAATACCGGCTTTAGACTTTAAGGTCGCTGAAGGTGGTACTAACTTCTCTATGGGACAACGTCAGTTGGTTTGCTTGGCT
             108285
Hz_ABCC2     CGTGCCATTCTACGCTCTAACAAAATACTCATCATGGACGAAGCTACCG
SPM-16 (774) CGTGCCATTCTACGCTCTAACAAAATACTCATCATGGACAAGGCTACCG
```

Figure S2. Alignment of *Helicoverpa zea* ATP binding cassette transporter subfamily C2 (*ABCC2*) genome sequence from 107,089 to 108,333 bp and amplicon sequence from knockout line SPM-16 with a deletion of 426 bp and an insertion of four bp at the sgRNA target sites in exon 21 and 22, respectively. Identical nucleotides are shown in white text with black background and alignment gaps are indicated by hyphens. Deletions and insertions caused by CRISPR/Cas9 editing at sgRNA target sites are marked with red text and nucleotide polymorphisms between the reference genomic scaffold (Accession: KY701524) sequence and amplicon sequences, including those may have been introduced by repair enzymes are shown in dark gray text with white background.

```
Hz_ABCC2 (99062) TGGAAGGCATTACGAAAGGCCTACTGGGTCTCCTACATGCCAGGAGCTATTTTTATCATCATTCAATCTGCAGCCAGGTA
```

```
WT           (57) TGGAAGGCATTACGAAAGGCCTACTGGGTCTCCTACATGCCAGGAGCTATTTTTATCATCATTCAATCTGCAGCCAGGTA
SPM-A28C      (1) TGGAAGGCATTACGAAAGGCCTACTGGGTCTCCTACATGCCAGGAGCTATTTTTATCATCATTCAATCTGCAGCCAGGTA

Hz_ABCC2  (99142) CACACTTTGTAAAGTATTTTTATTTAGTTATTTACGGCCAGTTTCTTCATCAAACATAAAAGTCAAAGTAATGTCTAAAG
WT          (137) CACACTTTGTAAAGTATTTTTATTTAGTTATTTACGGCCAGTTTCTTCATCAAACATAAAAGTCAAAGTAATGTCTAAAG
SPM-A28C     (81) CACACTTTGTAAAGTATTTTTATTTAGTTATTTACGGCCAGTTTCTTCATCAAACATAAAAGTCAAAGTAATGTCTAAAG

Hz_ABCC2  (99222) TAAAAGTAATGGTCAAATTCGTTTTTTCAAGGCTAGTGACAGCAAAACTAATAGAAAAAATGAATTTGACCGTTACTTTT
WT          (217) TAAAAGTAATGGTCAAATTCGTTTTT-CAAGGCTAGTGACAGCAAAACTAATAGAAAAAATGAATTTGACCGTTACTTTT
SPM-A28C    (161) TAAAAGTAATGGTCAAATTCGTTTTTTCAAGGCTAGTGACAGCAAAACTAATAGAAAAAATGAATTTGACCGTTACTTTT

Hz_ABCC2  (99302) ACTTTAAACATTACTTTGATTTTTACTTTGGATTTTACTTTTGATGAAGAAATTAGCTGTTAATGTTTAAGTTTCCCTAA
WT          (296) ATTTTAAACATTACTTTGATTTTTACTTTG-ATTTTACTTTTGATGAAGAAATTAGCTGTTAATGTTTAAGTTTCCCTAA
SPM-A28C    (241) ACTTTAAACATTACTTTGATTTTTACTTTGGATTTTACTTTTGATGAAGAAATTAGCTGTTAATGTTTAAGTTTCCCTAA

Hz_ABCC2  (99382) AAGTTAGATATAGTTGATCTATCTTTTAAATTTGGTTTCTTGATGGATCTTTAATTCTCTTCTTCAAAAGGTGTACTCAA
WT          (375) AAGTTAGATATAGTTGATCTATCTTTTAAATTTGGTTTCTTGATGGATCTTTAATTCTCTTCTTCAAAAGGTGTACTCAA
SPM-A28C    (321) AAGTTAGATATAGTTGATCTATCTTTTAAATTTGGTTTCTTGATGGATCTTTAATTCTCTTCTTCAAAAGGTGTACTCAA

Hz_ABCC2  (99462) AACTCAATTTTTTTATTAGCATTCCACGTGTTATACAGGTGGTCAAGGTATACTTGTGTGCTTCACAATGCAACACACTTG
WT          (455) AACTCAATTTTTTTATTAGCATTCCACGTGTTATACAGGTGGTCAAGGTATACTTGTGTGCTTCACAATGCAACACACTTG
SPM-A28C    (401) AACTCAATTTTTTTATTAGCATTCCACGTGTTATACAGGTGGTCAAGGTATACTTGTGTGCTTCACAATGCAACACACTTG

Hz_ABCC2  (99542) TGAAATCCCGTAAAGACGTATCGTGTTATTACTTATGAGTGATGATATATCAAATCAATACCTAGGGCTATTCCGTAACG
WT          (535) TGAAATCCCGTAAAGACGTATCGTGTTATTACTTATGAGTGATGATATATCAAATCAATACCTAGGGCTATTCCGTAACG
SPM-A28C    (481) TGAAATCCCGTAAAGACGTATCGTGTTATTACTTATGAGTGATGATATATCAAATCAATACCTAGGGCTATTCCGTAACG

Hz_ABCC2  (99622) GTTTCCGTAGAAATTAGATTTTTTTTTCGTCTCTAGAGCTTGATTAATTATAATCTTTTTATGTTTACAGGACGTATCAG
WT          (615) GTTTCCGTAGAAATTAGATTTTTTT--CGTCTCTAGAGCTTGATTAATTATAATCTTTTTATGTTTACAGGACGTATCAG
SPM-A28C    (561) GTTTCCGTAGAAATTAGATTTTTTT--CGTCTCTAGAGCTTGATTAATTATAATCTTTTTATGTTTACAGGACGTATCAG

Hz_ABCC2  (99702) CCGCTGTTGTTTTCTCAGTTACTGTCGTACTGGTCGGTGGACAGTGAAATGACTCAGCAAGACGCTGGCCTCTATGCTCT
WT          (693) CCGCTGTTGTTTTCTCAGTTACTGTCGTACTGGTCGGTGGACAGTGAAATGACTCAGCAAGACGCTGGCCTCTATGCTCT
SPM-A28C    (639) CCGCTGTTGTTTTCTCAGTTACTGTCGTACTGATACGACA----------------------------------------

Hz_ABCC2  (99782) CGCCATGCTGGGACTGAACTTCGTCTCCATGATGTGTCAGCACCACAACACACTGTTTGTGATGCGGTTCAGTTTAAAAG
WT          (773) CGCCATGCTGGGACTGAACTTCGTCTCCATGATGTGTCAGCACCACAACACACTGTTTGTGATGCGGTTCAGTTTAAAAG
SPM-A28C    (679) --------------------------------------------------------------------------------

Hz_ABCC2  (99862) TCAAGGTTGCCTGTTCTTCGCTTTTGTATAGGAAGGTGAGCATACTTTTAAGTAGAATAATCTTCCATCGATGTAATTAA
WT          (853) TCAAGGTTGCCTGTTCTTCGCTTTTGTATAGGAAGGTGAGCATACTTTTAAGTAGAATAATCTTCCATCGATGTAATTAA
SPM-A28C    (679) --------------------------------------------------------------------------------

Hz_ABCC2  (99942) ACAGGGACAATTAGACCGAAGTTAGGGTGCTTACATAAAGAAACAGGTTTCCGGTACAGATAAACTTGTACGGGTAGGTA
WT          (933) ACAGGGACAATTAGACCGAAGTTAGGGTGCTTACATAAAGAAACAGGTTTCCGGTACAGATAAACTTGTACGGGTAGGTA
SPM-A28C    (679) --------------------------------------------------------------------------------

Hz_ABCC2 (100022) CCGATCAGTGCAGGTATAATATTTCAATACAATCCTATTCACTCACTTATAAAGAAACAGGTTGTTTACCTGTTTCAACT
WT         (1013) CCGATCAGTGCAGGTATAATATTTCAATACAATCCTATTCACTCACTTATAAAGAAACAGGTTGTTTACCTGTTTCAACT
SPM-A28C    (679) --------------------------------------------------------------------------------

Hz_ABCC2 (100102) TACCTGTTTCTTTATAAGTGAGTGAATAGGATTGTATTGAAATATTATACCTGCACTGATCGGTACCTACCCGTACAGGT
WT         (1093) TACCTGTTTCTTTATAAGTGAGTGAATAGGATTGTATTGAAATATTATACCTGCACTGATCGGTACCTACCCGTACAGGT
SPM-A28C    (679) --------------------------------------------------------------------------------

Hz_ABCC2 (100182) TTGTCTGTACCGGTAACCTGTTTCTTTATGTAAACACCCTTAAGATCGTTAAATTAGTTTAATCAACCTATCAGCATTTT
WT         (1173) TTGTCTGTACCGGTAACCTGTTTCTTTATGTAAACACCCTTAAGATCGTTAAATTAGTTTAATCAACCTATCAGCATTTT
SPM-A28C    (679) --------------------------------------------------------------------------------

Hz_ABCC2 (100262) TATAGACAATATTTTTTTTTCAATAAAAAATTGGCCAGATCTGCTTTAAAAGAAGACGTTGACGTTGGCTTTAAAAGAGAC
WT         (1253) TATAGACAATATTTTTTTT-CAATAAAAAATTGGCCAGATCTGCTTTAAAAGAAGACGTTGACGTTGGCTTTAAAAGAGAC
SPM-A28C    (679) --------------------------------------------------------------------------------

Hz_ABCC2 (100342) TGATACTATTTTATTTATTTTTGATATCTTTATTTTAGATATACCTACTTACTTCTTTTTTGAAACTCGATAAAAAATTTC
WT         (1332) TGATACTATTTTATTTATTTTTGATATCTTTATTTTAGATATACCTACTTACTTCTTTTT-GAAACTCGATAAAAAATTTC
SPM-A28C    (679) --------------------------------------------------------------------------------

Hz_ABCC2 (100422) TTTGTTCTAGTTGCTCCGCATGACCCAAGTGTCGGTGGGTGAGGTGGCAGGAGGAAAGCTGGTGAACTTGCTGTCCAACG
WT         (1411) TTTGTTCTAGTTGCTCCGCATGACCCAAGTGTCGGTGGGTGAGGTGGCAGGAGGAAAGCTGGTGAACTTGCTGTCCAACG
SPM-A28C    (679) --------------------------------------------------------------------------------
```

```
Hz_ABCC2(100502)ATATCACGAGGTTCGACTACGCGTTCATGTTCCTACATTACTTGTGGATCGTGCCTATCCAAGTGGCTGTTGTTTTGTAC
WT        (1491)ATATCACGAGGTTCGACTACGCGTTCATGTTCCTACATTACTTGTGGATCGTGCCTATCCAAGTGGCTGTTGTTTTGTAT
SPM-A28C   (679)--------------------------------------------------------------------------------

Hz_ABCC2(100582)TTCTTGTGGGAGGCTGCTGGCTTCGCACCCTTCGTCGGTCTGTTTGGAGTCGTTATACTGATTTTACCACTGCAAGGTAT
WT        (1571)TTCTTGTGGGAGGCTGCTGGCTTCGCACCCTTCGTCGGTCTGTTTGGAGTCGTTATACTGATTTTACCACTGCAAGGTAT
SPM-A28C   (679)--------------------------------------------------------------------------------

Hz_ABCC2(100662)GTCATATTAAAACCTTTTTTCAATTTAATATTGTTTCCTTATGTATCCATGTTATTAATCGATGAACTTTTGGCTTTTAG
WT        (1651)GTCATATTAAAACCTTTTTTCAATTTAATATTGTTTCCTTATGTATCCATGTTATTAATCGATGAACTTTTGGCTTTTAG
SPM-A28C   (679)--------------------------------------------------------------------------------

Hz_ABCC2(100742)CTGGCTTGACGAAACTCACAACTGTTGTAAGACGTGAGACGGCTAAGAGAACGGACAGGCGAATTAAACTAATGAGTGAA
WT        (1731)CTGGCTTGACGAAACTCACAACTGTTGTAAGACGTGAGACGGCTAAGAGAACGGACAGGCGAATTAAACTAATGAGTGAA
SPM-A28C   (679)--------------------------------------------------------------------------------

Hz_ABCC2(100822)ATTATTGGTGGTATTCAGGTAAAAAAAACTTTTCTTAATTTTTATATATTGACCTATTTAAAATGATGACAAAAATTAAC
WT        (1811)ATTATTGGTGGTATTCAGTAAAAAAAAACTTTTCTTAATTTTTATATATTGACCTATTTAAAATGATGACAAAAATTAAC
SPM-A28C   (679)--------------------------------------------------------------------------------

Hz_ABCC2(100902)TGTTTGGTCAATTTCAGGTCATTAAAATGTACGCTTGGGAGAAACCCTTCCAGCTAGTTGTGAAGGCAGCTCGTGCCTTT
WT        (1891)TGTTTGGTCAATTTCAGGTCATTAAAATGTACGCTTGGGAGAAACCCTTCCAGCTAGTTGTGAAGGCAGCTCGTGCCTTT
SPM-A28C   (679)--------------------------------------------------------------------------------

Hz_ABCC2(100982)GAAATGAGTGCCCTCAGGAAGTCCATCTTCATCAGGAGTACTTTCCTAGGGTTCATGTTGTTCACTGAGCGAAGCATCAT
WT        (1971)GAAATGAGTGCCCTCAGGAAGTCCATCTTCATCAGGAGTACTTTCCTAGGGTTCATGTTGTTCACTGAGCGAAGCATCAT
SPM-A28C   (679)--------------------------------------------------------------------------------

Hz_ABCC2(101062)GTTTGTCACAGTGTTGACACTCGCTCTCACAGGCACTATGATTACTGCCACTACGGTAAGATTTACTTATTATAATAATT
WT        (2051)GTTTGTCACAGTGTTGACACTCGCTCTCACAGGCACTATGATTACTGCCACTACGGTAAGATTTACTTATTATAATAATT
SPM-A28C   (679)--------------------------------------------------------------------------------

Hz_ABCC2(101142)AAATATAAGATATTTTGCCGACCGTTTCATTCCATATATATTAATGGTTTTCGAAATATCGCATCAATCGATTCATCATC
WT        (2131)AAATATAAGATATTTTGCCGACCGTTTCATTCCATATATATTAATGGTTTTCGAAATATCGCATCAATCGATTCATCATC
SPM-A28C   (679)--------------------------------------------------------------------------------

Hz_ABCC2(101222)GATTTCCATTCAATTTCGGCAAATTACTACTTAACTAAGTTTGAACAATCGTTCCGATATATAATCTCGATATTATTTCA
WT        (2211)GATTTCCATTCAATTTCGGCAAATTACTACTTAACTAAGTTTGAACAATCGTTCCGATATATAATCTCGATATTATTTCA
SPM-A28C   (679)--------------------------------------------------------------------------------

Hz_ABCC2(101302)CAGATATACCCTATTCAACAGTACTTCAGTATTATTCAGTTTAACGTAACACTGATCATTCCTATGGCAATCGCAAGTTA
WT        (2291)CAGATATACCCTATTCAACAGTACTTCAGTATTATTCAGTTTAACGTAACACTGATCATTCCTATGGCAATCGCAAGTTA
SPM-A28C   (679)--------------------------------------------------------------------------------

Hz_ABCC2(101382)TTCCGAGATGATGGTGTCCATAGAACGTATCCAGGGATTCCTTAGTTTGGGTAAATATTGTTTCTTTTAAGTTCAATAAA
WT        (2371)TTCCGAGATGATGGTGTCCATAGAACGTATCCAGGGATTCCTTAGTTTGGGTAAATATTGTTTCTTTTAAGTTCAATAAA
SPM-A28C   (679)--------------------------------------------------------------------------------

Hz_ABCC2(101462)ATTTACACACATTAGGTAGATTGGTATAAAGCGTACAGTGATAATAATACTGAGATTTGATATTTATGTTTTGTAAACAG
WT        (2451)ATTTACACACATTAGGTAGATTGGTATAAAGCGTACAGTGATAATAATACTGAGATTTGATATTTATGTTTTGTAAACAG
SPM-A28C   (679)--------------------------------------------------------------------------------

Hz_ABCC2(101542)ACGAGCGGTCCGACATGCAAGTGACTCCAAAAATTAATGGATCTAACAATAACACTTTGTTTAAATCCAAGAAGTCACCA
WT        (2531)ACGAGCGGTCCGACATGCAAGTGACTCCAAAAATTAATGGATCTAACAATAACACTTTGTTTAAATCCAAGAAGTCACCA
SPM-A28C   (679)--------------------------------------------------------------------------------

Hz_ABCC2(101622)CTTGAAGTAGGCATCGTGCCTAAGAAATACTCACCTAGTGAAGTTATGGCTGCAAAGGAGATGCAGGATGATCCTACCCA
WT        (2611)CTTGAAGTAGGCATCGTGCCTAAGAAATACTCACCTAGTGAAGTTATGGCTGCAAAGGAGATGCAGGATGATCCTACCCA
SPM-A28C   (679)--------------------------------------------------------------------------------

Hz_ABCC2(101702)GATGGACTATCCTATCAGGCTTAACAAAGTAAGCGCATCGTGGACCGGCAGCAATAGTTCTTCAGAAATGACACTTAAGA
WT        (2691)GATGGACTATCCTATCAGGCTTAACAAAGTAAGCGCATCGTGGACCGGCAGCAATAGTTCTTCAGAAATGACACTTAAGA
SPM-A28C   (679)--------------------------------------------------------------------------------

Hz_ABCC2(101782)ATATATCGTTACGTATTCGTAAAGGAAAATTGTGTGCTATCATTGGTCCTGTGGGGTCCGGAAAGGTAATTTATTTCATT
WT        (2771)ATATATCGTTACGTATTCGTAAAGGAAAATTGTGTGCTATCATTGGTCCTGTGGGGTCCGGAAAGGTAATTTATTTCATT
SPM-A28C   (679)--------------------------------------------------------------------------------

Hz_ABCC2(101862)ATAAATATTTGTTCATATACAGATTTGTTTTTTTTATTTTTTACTTGTAATATTAATATTTTAACTTTTACAGACATCTC
WT        (2851)ATAAATATTTGTTCATATACAGATTTGTTTTTTTTATTTTTTACTTGTAATATTAATATTTTAACTTTTACAGACATCTC
SPM-A28C   (679)--------------------------------------------------------------------------------
```

```
Hz_ABCC2(101942) TACTGCAACTGCTCTTAAAAGAATTACCATTGAATAGTGGTACACTTGACGTGAGCGGGAAAATGTCGTACGCTTGTCAA
WT        (2931) TACTGCAACTGCTCTTAAAAGAATTACCATTGAATAGTGGTACACTTGACGTGAGCGGGAAAATGTCGTACGCTTGTCAA
SPM-A28C   (679) --------------------------------------------------------------------------------

Hz_ABCC2(102022) GAGTCCTGGCTGTTCCCAGGCACAGTACGAGAAAACATTTTGTTCGGCCTAACTTACGAACCCACAAAATACAAAGAGGT
WT        (3011) GAGTCCTGGCTGTTCCCAGGCACAGTACGAGAAAACATTTTGTTCGGCCTAACTTACGAACCCACAAAATACAAAGAGGT
SPM-A28C   (679) --------------------------------------------------------------------------------

Hz_ABCC2(102102) AAATTATTGCAGGTTATGAATGACTTTATTAAATTTTTATTTAGGTGCATAATTATAATAATAATGCGTTTATTTCATGA
WT        (3091) AAATTATTGCAGGTTATGAATGACTTTATTAAATTTTTATTTAGGTGCATAATTATAATAATAATGCGTTTATTTCATGA
SPM-A28C   (679) --------------------------------------------------------------------------------

Hz_ABCC2(102182) CTACAATGGTCCAATTGTTAGTAACTTTTCAACTATAACTTATCGACTAAGGGTTAGTAATACCTAAAAACTAAACGTTT
WT        (3171) CTACAATGGTCCAATTGTTAGTAACTTTTCAACTATAACTTATCGACTAAGGGTTAGTAATACCTAAAAACTAAACGTTT
SPM-A28C   (679) --------------------------------------------------------------------------------

Hz_ABCC2(102262) GCCTAACAAGCAAGTCCATCCAATGCCTAAGCACCGGAGCATCATGCTTGTCGGCCAAGACTTGTAGGATGCTGTTGTAG
WT        (3251) GCCTAACAAGCAAGTCCATCCAATGCCTAAGCACCGGAGCATCATGCTTGTCGGCCAAGACTTGTAGGATGCTGTTGTAG
SPM-A28C   (679) --------------------------------------------------------------------------------

Hz_ABCC2(102342) GATGATGTAATTTATCTTCTATATAATTATATTTAATAATTAATTTATTATAAATAATTAAATTAATAATAATTAATAAT
WT        (3331) GATGATGTAATTTATCTTCTATATAATTATATTTAATAATTAATTTATTATAAATAATTAAATTAATAATAATTAATAAT
SPM-A28C   (679) --------------------------------------------------------------------------------

Hz_ABCC2(102422) TATATAGAAGATATTTTTTTCTAACTAGCTGATCCCGTGAACTTCGTATCGTTCAAACCTTCCCTGGACCTCTACAAACA
WT        (3411) TATATAGAAGATATTTTTT-CTAACTAGCTGATCCCGTGAACTTCGTATCGTTCAAACCTTCCCTGGACCTCTACAAACA
SPM-A28C   (679) --------------------------------------------------------------------------------

Hz_ABCC2(102502) TTTTAAAACCAAAATTAGCTCAATCGGCCCAGCCGTCCTCGAGTTTTAATCAGACTAACGAACAATAATCCATTTTTATT
WT        (3490) TTTTAAAACCAAAATTAGCTCAATCGGCCCAGCCGTCCTCGAGTTTTAATCAGACTAACGAACAATAATCCATTTTTATT
SPM-A28C   (679) --------------------------------------------------------------------------------

Hz_ABCC2(102582) TATATAGATTATATTTCTTGTGCGAATTATCTTATGTAGGTATGTCTTCCAAAACATTCATACTTACACCTGTATTTCTT
WT        (3570) TATATAGATTATATTTCTTGTGCGAATTATCTTATGTAGGTATGTCTTCCAAAACATTCATACTTACACCTGTATTTCTT
SPM-A28C   (679) --------------------------------------------------------------------------------

Hz_ABCC2(102662) GCGATAGCTTTAAGTTATATTCTTCACCAAAATTAAAAATATGAAAAAGATATGATGTTCAACCACAAACAGAGATTGCA
WT        (3650) GCGATAGCTTTAAGTTATATTCTTCACCAAAATTAAAAATATGAAAAAGATATGATGTTCAACCACAAACAGAGATTGCA
SPM-A28C   (679) --------------------------------------------------------------------------------

Hz_ABCC2(102742) CGAGAAACGTTAAAATTTTATAGTCAATTTTCCCGCTGTTTTATAATTCACCCAATTTTCATCTGCCATGGTCTGTTATG
WT        (3730) CGAGAAACGTTAAAATTTTATAGTCAATTTTCCCGCTGTTTTATAATTCACCCAATTTTCATCTGCCATGGTCTGTTATG
SPM-A28C   (679) --------------------------------------------------------------------------------

Hz_ABCC2(102822) CATGTAGATATATTTTGTTATGGCAGGTTTGCAAGGTGTGTTCGTTGCTGCCTGACTTCAAGCAGTTCCCGTATGGTGAC
WT        (3810) CATGTAGATATATTTTGTTATGGCAGGTTTGCAAGGTGTGTTCGTTGCTGCCTGACTTCAAGCAGTTCCCGTATGGTGAC
SPM-A28C   (679) --------------------------------------------------------------------------------

Hz_ABCC2(102902) CTGTCTTTAGTGGGAGAACGAGGAGTGTCCCTGTCAGGTGGTCAAAGAGCCAGGATCAATTTGGCTAGAGCAATTTATCG
WT        (3890) CTGTCTTTAGTGGGAGAACGAGGAGTGTCCCTGTCAGGTGGTCAAAGAGCCAGGATCAATTTGGCTAGAGCAATTTATCG
SPM-A28C   (679) --------------------------------------------------------------------------------

Hz_ABCC2(102982) TGAGGTAAGCATCTTTCGTTTACGCAGAACTTCTACACATGGTAATAAAACACTTCAGTTGGTTATTCGTGATTCGTAAA
WT        (3970) TGAGGTAAGCATCTTTCGTTTACGCAGAACTTCTACACATGGTAATAAAACACTTCAGTTGGTTATTCGTGATTCGTAAA
SPM-A28C   (679) --------------------------------------------------------------------------------

Hz_ABCC2(103062) TCGAGGTATTGATGAAGGACTATATTATGTTCTTGCGACTATTCCTGTTATATGATATCGCAACCACAAGATTAATTGTC
WT        (4050) TCGAGGTATTGATGAAGGACTATATTATGTTCTTGCGACTATTCCTGTTATATGATATCGCAACCACAAGATTAATTGTC
SPM-A28C   (679) --------------------------------------------------------------------------------

Hz_ABCC2(103142) GGGGTAATACCTGATAACGTGAAAGTAAACAATTGGTAACAACTTAGTTATATCGGCACTGTAATAATGCGACAGTATAT
WT        (4130) GGGGTAATACCTGATAACGTGAAAGTAAACAATTGGTAACAACTTAGTTATATCGGCACTGTAATAATGCGACAGTATAT
SPM-A28C   (679) --------------------------------------------------------------------------------

Hz_ABCC2(103222) GCGATACATAATTTGTTTACATTTAACTTTTCTAACAATTTATCAATGAAATCTTTTACATATTGATGAGAGCAACTTAT
WT        (4210) GCGATACATAATTTGTTTACATTTAACTTTTCTAACAATTTATCAATGAAATCTTTTACATATTGATGAGAGCAACTTAT
SPM-A28C   (679) --------------------------------------------------------------------------------

Hz_ABCC2(103302) ACATTGTTGTCATTACACCACATTTTCTTAAATCGTGAGCGTGAAACTAAAATCGTGGGCGTATTTTCAGGCCGACATTT
WT        (4290) ACATTGTTGTCATTACACCACATTTTCTTAAATCGTGAGCGTGAAACTAAAATCGTGGGCGTATTTTCAGGCCGACATTT
```

```
SPM-A28C    (679)--------------------------------------------------------------------------------

Hz_ABCC2(103382)ACTTGCTGGATGATCCCCTATCTGCAGTGGACGCTAATGTAGGCAGACAACTGTTTGATGGCTGCATCAAAGGCTACCTC
WT         (4370)ACTTGCTGGATGATCCCCTATCTGCAGTGGACGCTAATGTAGGCAGACAACTGTTTGATGGCTGCATCAAAGGCTACCTC
SPM-A28C    (679)--------------------------------------------------------------------------------

Hz_ABCC2(103462)ACTGGACGAACTTGCGTCTTGGTCACCCATCAGATCCATTACCTCAAAGCTGCTGATTTTATTGTAGTCCTTAATGAGGC
WT         (4450)ACTGGACGAACTTGCGTCTTGGTCACCCATCAGATCCATTACCTCAAAGCTGCTGATTTTATTGTAGTCCTTAATGAGGC
SPM-A28C    (679)--------------------------------------------------------------------------------

Hz_ABCC2(103542)AAGTATGACTACCTATCATTTCATATTTCCATTGAAAGATAACAAAAGTATTTACATTAAAAATATATTTATTGAAGTGA
WT         (4530)AAGTATGACTACCTATCATTTCATATTTCCATTGAAAGATAACAAAAGTATTTACATTAAAAATATATTTATTGAAGTGA
SPM-A28C    (679)--------------------------------------------------------------------------------

Hz_ABCC2(103622)TTAATTTTATCTTAACTATTATCTTTGGACGAGTCATTACAACGTAGCAAATTTCCAACACTTCAATATTTACTCTTTGA
WT         (4610)TTAATTTTATCTTAACTATTATCTTTGGACGAGTCATTACAACGTAGCAAATTTCCAACACTTCAATATTTACTCTTTGA
SPM-A28C    (679)--------------------------------------------------------------------------------

Hz_ABCC2(103702)ATACTACTTACTTAGTCCAGCTTCAAAATTAATAAAAGTTTCCTAATGGTTTTTTATCAGGGTTCCGTCGAGAATATGGG
WT         (4690)ATACTACTTACTTAGTCCAGCTTCAAAATTAATAAAAGTTTCCTAATGGTTTTTTATCAGGGTTCCGTCGAGAATATGGG
SPM-A28C    (679)--------------------------------------------------------------------------------

Hz_ABCC2(103782)CACTTATGATGAGCTGGTGAAGACAGGAACTGAATTCTCGATGCTGCTATCCAACCAAGAAAATGACGCAACTGAAAACG
WT         (4770)CACTTATGATGAGCTGGTGAAGACAGGAACTGAATTCTCGATGCTGCTATCCAACCAAGAAAATGACGCAACTGAAAACG
SPM-A28C    (679)--------------------------------------------------------------------------------

Hz_ABCC2(103862)AAAAGAAAGTAAGTATGCACTTAATATCTGGTATTTTCCGTGGCGACTTGAACGTTTGCCAGAAAACAGCTTTCAACATT
WT         (4850)AAAAGAAAGTAAGTATGCACTTAATATCTGGTATTTTCCGTGGCGACTTGAACGTTTGCCAGAAAACAGCTTTCAACATT
SPM-A28C    (679)--------------------------------------------------------------------------------

Hz_ABCC2(103942)ATCTTGTCTACATCGTTGCACATGTTCCTAAAACATATCCGAATAAAAAATATAGGTATGAACGAGTATACATCTACCCT
WT         (4930)ATCTTGTCTACATCGTTGCACATGTTCCTAAAACATATCCGAATAAAAAATATAGGTATGAACGAGTATACATCTACCCT
SPM-A28C    (679)--------------------------------------------------------------------------------

Hz_ABCC2(104022)CGTGTATTGTAATATGTACTGAATAAACATTGTTCATTTTAAATTCTCCTAATAGATTATGCTTAAAAATGCAGCATAGA
WT         (5010)CGTGTATTGTAATATGTACTGAATAAACATTGTTCATTTTAAATTCTCCTAATAGATTATGCTTAAAAATGCAGCATAGA
SPM-A28C    (679)--------------------------------------------------------------------------------

Hz_ABCC2(104102)TTACACTGGACCTTGTTCAAACAACCCAGAAAAAAATACTATTTACGTTCCTTTCGTCTTAGGACCGACCAGCAATGATG
WT         (5090)TTACACTGGACCTTGTTCAAACAACCCAGAAAAAAATACTATTTACGTTCCTTTCGTCTTAGGACCGACCAGCAATGATG
SPM-A28C    (679)--------------------------------------------------------------------------------

Hz_ABCC2(104182)CGAGGAATATCAAAAATCTCAGTTAAGAGCGACACCGAAATGGAACAGAAGGCTCAAATACAGGAGGCAGAGGAGAGAGC
WT         (5170)CGAGGAATATCAAAAATCTCAGTTAAGAGCGACACCGAAATGGAACAGAAGGCTCAAATACAGGAGGCAGAGGAGAGAGC
SPM-A28C    (679)--------------------------------------------------------------------------------

Hz_ABCC2(104262)GACAGGTAGCTTGAAGTTTGAGGTGGTGCTCAAGTACCTGAGCTCCGTCCAGTCCTGGTGTCTGGTGTTTACGGCGTTCC
WT         (5250)GACAGGTAGCTTGAAGTTTGAGGTGGTGCTCAAGTACCTGAGCTCCGTCCAGTCCTGGTGTCTGGTGTTTACGGCGTTCC
SPM-A28C    (679)--------------------------------------------------------------------------------

Hz_ABCC2(104342)TTGTACTGCTGATCACGCAAGGTGCTGCCACCACTGCCGACTATTGGTTGAGTTTCTGGTAAGATAATGAATTATTTTTC
WT         (5330)TTGTACTGCTGATCACGCAAGGTGCTGCCACCACTGCCGACTATTGGTTGAGTTTCTGGTAAGATAATGAATTATTTTTC
SPM-A28C    (679)------------------------------------CCGACTATTGGTTGAGTTTCTGGTAAGATAATGAATTATTTTTC

Hz_ABCC2(104422)AAAAATTTAATTTAACGGAAACAAAGAACGTATTTTAAATTGGCTTCTCTGAATATGTCTTAAATGTCTCCTGATAGTTA
WT         (5410)AAAAATTTAATTTAACGGAAACAAAGAACGTATTTTAAATTGGCTTCTCTGAATATGTCTTAAATGTCTCCTGATAGTTA
SPM-A28C    (723)AAAAATTTAATTTAACGGAAACAAAGAACGTATTTTAAATTGGCTTCTCTGAATATGTCTTAAATGTCTCCTGATAGTTA

Hz_ABCC2(104502)ACATTTTGATTGCAGGACTAACCAAGTGGATTCTTATGAACAATCTTTACCTGAAGGCGTGGATCCAGGTAAGTCTACGA
WT         (5490)ACATTTTGATTGCAGGACTAACCAAGTGGATTCTTATGAACAATCTTTACCTGAAGGCGTGGATCCAGGTAAGTCTACGA
SPM-A28C    (803)ACATTTTGATTGCAGGACTAACCAAGTGGATTCTTATGAACAATCTTTACCTGAAGGCGTGGATCCAGGTAAGTCTACGA

Hz_ABCC2(104582)AATATCTCTGTTTTATTTATATGAACTTCCTGCGTATGTACCATTTGCAGTTCTAAGATCTCAACCTCAACTAAAAAGTT
WT         (5570)AATATCTCTGTTTTATTTATATGAACTTCCTGCGTATGTACCATTTGCAGTTCTAAGATCTCAACCTCAACTAAAAAGTT
SPM-A28C    (883)AATATCTCTGTTTTATTTATATGAACTTCCTGCGTATGTACCATTTGCAGTTCTAAGATCTCAACCTCAACTAAAAAGTT

Hz_ABCC2(104662)ATTTCTTTTCAGATACTGACATGAACGCACAAATTGGCTTACTTACAACTGCCCAGTACCTATACGTTTTCGGTGGAGTT
WT         (5650)ATTTCTTTTCAGATACTGACATGAACGCACAAATTGGCTTACTTACAACTGCCCAGTACCTATACGTTTTCGGTGGAGTT
SPM-A28C    (963)ATTTCTTTTCAGATACTGACATGAACGCACAAATTGGCTTACTTACAACTGCCCAGTACCTATACGTTTTCGGTGGAGTT

Hz_ABCC2(104742)ATATTGGCTTTAATAGTCATGACCCTCGTCAGAATCACAGCTTTCGTAGCGATGACAATGCGAGCTTCTCAAAATCTTCA
```

```
WT         (5730) ATATTGGCTTTAATAGTCATGACCCTCGTCAGAATCACAGCTTTCGTAGCGATGACAATGCGAGCTTCTCAAAATCTTCA
SPM-A28C   (1043) ATATTGGCTTTAATAGTCATGACCCTCGTCAGAATCACAGCTTTCGTAGCGATGACAATGCGAGCTTCTCAAAATCTTCA

Hz_ABCC2(104822) CAACACTATTTACGAAAAATTAATTGTGACAGTAATGAGATTTTTCGATACCAATCCTTCTGGTGAGTAACATTCATCAT
WT         (5810) CAACACTATTTACGAAAAATTAATTGTGACAGTAATGAGATTTTTCGATACCAATCCTTCTGGTGAGTAACATTCATCAT
SPM-A28C   (1123) CAACACTATTTACGAAAAATTAATTGTGACAGTAATGAGATTTTTCGATACCAATCCTTCTGGTGAGTAACATTCATCAT

Hz_ABCC2(104902) TACACATAACATTATTTATTTAAGTGATCACATAAAAAAGTGCCATAGCATTTTGCGAGACAGTCAGTTAGATAAGTAGA
WT         (5890) TACACATAACATTATTTATTTAAGTGATCACATAAAAAAGTGCCATAGCATTTTGCGAGACAGTCAGTTAGATAAGTAGA
SPM-A28C   (1203) TACACATAACATTATTTATTTAAGTGATCACATAAAAAAGTGCCATAGCATTTTGCGAGACAGTCAGTTAGATAAGTAGA

Hz_ABCC2(104982) GATAATGATTTTAAAATCAAAGTAACGGACTCTCTCAATGTTAGAAGTGTAGGTAATTAGCGATAATTGCAGCTGGCCAA
WT         (5970) GATAATGATTTTAAAATCAAAGTAACGGACTCTCTCAATGTTAGAAGTGTAGGTAATTAGCGATAATTGCAGCTGGCCAA
SPM-A28C   (1283) GATAATGATTTTAAAATCAAAGTAACGGACTCTCTCAATGTTAGAAGTGTAGGTAATTAGCGATAATTGCAGCTGGCCAA

Hz_ABCC2(105062) GTGCCCAGGTTGCCGTGAGTAGACCTTAACATTAGTACACGCCAATTGCAACGACGGTAGCCAAGATAGCTTGGATAATA
WT         (6050) GTGCCCAGGTTGCCGTGAGTAGACCTTAACATTAGTACACGCCAATTGCAACGACGGTAGCCAAGATAGCTTGGATAATA
SPM-A28C   (1363) GTGCCCAGGTTGCCGTGAGTAGACCTTAACATTAGTACACGCCAATTGCAACGACGGTAGCCAAGATAGCTTGGATAATA

Hz_ABCC2(105142) ACAACATCTTTAATGAGTTCTGCAACTTTCGACTTGTGATTTTGGTGGCAGTAGATTTAAGAAAACGTTGTACATATGTG
WT         (6130) ACAACATCTTTAATGAGTTCTGCAACTTTCGACTTGTGATTTTGGTGGCAGTAGATTTAAGAAAACGTTGTACATATGTG
SPM-A28C   (1443) ACAACATCTTTAATGAGTTCTGCAACTTTCGACTTGTGATTTTGGTGGCAGTAGATTTAAGAAAACGTTGTACATATGTG

Hz_ABCC2(105222) TAGTATAAGATGATGATACATTCTAGGCTATCCGTATTTTTTTAGATTATGATTTATTAAATGCAAGAAGATGATGCAAT
WT         (6210) TAGTATAAGATGATGATACATTCTAGGCTATCCGTATTTTTTTAGATTATGATTTATTAAATGCAAGAAGATGATGCAAT
SPM-A28C   (1523) TAGTATAAGATGATGATACATTCTAGGCTATCCGTATTTTTTTAGATTATGATTTATTAAATGCAAGAAGATGATGCAAT

Hz_ABCC2(105302) GAAATTAATTTACAACATCATGCTTATTGGAGCTTCTTTTGAATTTCGCAGGTCGTGTGCTGAACAGATTCTCAAAAGAC
WT         (6290) GAAATTAATTTACAACATCATGCTTATTGGAGCTTCTTTTGAATTTCGCAGGTCGTGTGCTGAACAGATTCTCAAAAGAC
SPM-A28C   (1603) GAAATTAATTTACAACATCATGCTTATTGGAGCTTCTTTTGAATTTCGCAGGTCGTGTGCTGAACAGATTCTCAAAAGAC

Hz_ABCC2(105382) ATGGGTGCCATGGATGAGCTTCTTCCTCGCAGTCTTTTAGAAACAGTTCAGATGTATTTGTCGCTGACCAGTGTGTTGGT
WT         (6370) ATGGGTGCCATGGATGAGCTTCTTCCTCGCAGTCTTTTAGAAACAGTTCAGATGTATTTGTCGCTGACCAGTGTGTTGGT
SPM-A28C   (1683) ATGGGTGCCATGGATGAGCTTCTTCCTCGCAGTCTTTTAGAAACAGTTCAGATGTATTTGTCGCTGACCAGTGTGTTGGT

Hz_ABCC2(105462) GCTGAACGCCACTGCATTGCCCTGGACGCTTATACCTACCTCCGTGTTGATTGTCATCTTCGTGCTCATGTTGAGATGGT
WT         (6450) GCTGAACGCCACTGCATTGCCCTGGACGCTTATACCTACCTCCGTGTTGATTGTCATCTTCGTGCTCATGTTGAGATGGT
SPM-A28C   (1763) GCTGAACGCCACTGCATTGCCCTGGACGCTTATACCTACCTCCGTGTTGATTGTCATCTTCGTGCTCATGTTGAGATGGT

Hz_ABCC2(105542) ACCTGAATACAGCTCAGGCTGTCAAACGTTTGGAGGGCACAAGTAAGTGTTACATGAACCATAGGCGTTATTTAAGAACA
WT         (6530) ACCTGAATACAGCTCAGGCTGTCAAACGTTTGGAGGGCACAAGTAAGTGTTACATGAACCATAGGCGTTATTTAAGAACA
SPM-A28C   (1843) ACCTGAATACAGCTCAGGCTGTCAAACGTTTGGAGGGCACAAGTAAGTGTTACATGAACCATAGGCGTTATTTAAGAACA

Hz_ABCC2(105622) AATATTGATTTAGAGTGTAAATTAATTAACGGTTAGAACATCGCTAATAGTTTTGACTGCCTCGTTGGTCTAGAGGTCGC
WT         (6610) AATATTGATTTAGAGTGTAAATTAATTAACGGTTAGAACATCGCTAATAGTTTTGACTGCCTCGTTGGTCTAGAGGTCGC
SPM-A28C   (1923) AATATTGATTTAGAGTGTAAATTAATTAACGGTTAGAACATCGCTAATAGTTTTGACTGCCTCGTTGGTCTAGAGGTCGC

Hz_ABCC2(105702) AAGCGCGGCTGCTGTGCTCGAGGTCTCGGGTTCGATTCCCGGGTCAGGCCGAAATCGCTTTGTGGGTTTTCATAAACTTT
WT         (6690) AAGCGCGGCTGCTGTGCTCGAGGTCTCGGGTTCGATTCCCGGGTCAGGCCGAAATCGCTTTGTGGGTTTTCATAAACTTT
SPM-A28C   (2003) AAGCGCGGCTGCTGTGCTCGAGGTCTCGGGTTCGATTCCCGGGTCAGGCCGAAATCGCTTTGTGGGTTTTCATAAACTTT

Hz_ABCC2(105782) CACAAAGCAGCCCGTAGCCTGGAAGTTGGTGATTGATTCACCCGTGCATCGGAGAGCACGTAAATGTCGGTCCTGCGCCT
WT         (6770) CACAAAGCAGCCCGTAGCCTGGAAGTTGGTGATTGATTCACCCGTGCATCGGAGAGCACGTAAATGTCGGTCCTGCGCCT
SPM-A28C   (2083) CACAAAGCAGCCCGTAGCCTGGAAGTTGGTGATTGATTCACCCGTGCATCGGAGAGCACGTAAATGTCGGTCCTGCGCCT

Hz_ABCC2(105862) GATCTCTTTCCGGTCGTGTCGGATTGCCGTCCCATCGGGTTATGAGAGTGAAGGAATAGGGAGTGCACCTGTGTCTGCGC
WT         (6850) GATCTCTTTCCGGTCGTGTCGGATTGCCGTCCCATCGGGTTATGAGAGTGAAGGAATAGGGAGTGCACCTGTGTCTGCGC
SPM-A28C   (2163) GATCTCTTTCCGGTCGTGTCGGATTGCCGTCCCATCGGGTTATGAGAGTGAAGGAATAGGGAGTGCACCTGTGTCTGCGC

Hz_ABCC2(105942) AAATGCTCGTGCACTATAATATGTCCTGCGCAGCTGGCTGATCTCCTTTAATGAGAACAGCCGCCGTGGCCGAAATCGGC
WT         (6930) AAATGCTCGTGCACTATAATATGTCCTGCGCAGCTGGCTGATCTCCTTTAATGAGAACAGCCGCCGTGGCCGAAATCGGC
SPM-A28C   (2243) AAATGCTCGTGCACTATAATATGTCCTGCGCAGCTGGCTGATCTCCTTTAATGAGAACAGCCGCCGTGGCCGAAATCGGC

Hz_ABCC2(106022) CGTGGACGCCATTATTAATAGTTTTGAAATAAAATTATTTTAATTTTGTTTTATTTCTACTTACAGCCAAGAGTCCTGTA
WT         (7010) CGTGGACGCCATTATTAATAGTTTTGAAATAAAATTATTTTAATTTTGTTTTATTTCTACTTACAGCCAAGAGTCCTGTA
SPM-A28C   (2323) CGTGGACGCCATTATTAATAGTTTTGAAATAAAATTATTTTAATTTTGTTTTATTTCTACTTACAGCCAAGAGTCCTGTA

Hz_ABCC2(106102) TTTGGAATGATTAACTCCACTATTTCTGGACTCTCCACTATTAGAAGTTCGGGTTCCCAGGATAGACAGATGAAATTGTT
WT         (7090) TTTGGAATGATTAACTCCACTATTTCTGGACTCTCCACTATTAGAAGTTCGGGTTCCCAGGATAGACAGATGAAATTGTT
SPM-A28C   (2403) TTTGGAATGATTAACTCCACTATTTCTGGACTCTCCACTATTAGAAGTTCGGGTTCCCAGGATAGACAGATGAAATTGTT
```

```
Hz_ABCC2(106182) TGACGAAGCGCAGGTAAACATGAAGCTTATTATTTTTTATATTAATTAAAATTACATCTAAAAAGATTTATCCAATAGT
WT        (7170) TGACGAAGCGCAGGTAAACATGAAGCTTATTATTTTTTATATTAATTAAAATTACATCTAAAAAGATTTATCCAATAGT
SPM-A28C  (2483) TGACGAAGCGCAGGTAAACATGAAGCTTATTATTTTTTATATTAATTAAAATTACATCTAAAAAGATTTATCCAATAGT

Hz_ABCC2(106262) AATGGTTATATATTTCTTATTGCTGTTTACAGAATCTCCACACAAGTGCTTTCCACACATTCTTCGGCGGTTCTACGGCA
WT        (7250) AATGGTTATATATTTCTTATTGCTGTTTACAGAATCTCCACACAAGTGCTTTCCACACATTCTTCGGCGGTTCTACGGCA
SPM-A28C  (2563) AATGGTTATATATTTCTTATTGCTGTTTACAGAATCTCCACACAAGTGCTTTCCACACATTCTTCGGCGGTTCTACGGCA

Hz_ABCC2(106342) TTTGCATTGTATCTCGATACTTTGTGTTTGACCTACCTCGGTGTCGTCATGTCAATTTTCATTTTGGGCGACTTTGGTGA
WT        (7330) TTTGCATTGTATCTCGATACTTTGTGTTTGACCTACCTCGGTGTCGTCATGTCAATTTTCATTTTGGGCGACTTTGGTGA
SPM-A28C  (2643) TTTGCATTGTATCTCGATACTTTGTGTTTGACCTACCTCGGTGTCGTCATGTCAATTTTCATTTTGGGCGACTTTGGTGA

Hz_ABCC2(106422) GCACTTTTTCTTAGGCACATTAAAAGGCAATATTTACACATTTCCAGTTCATAGTGTGATATCTTTGGCATTACCATTCC
WT        (7410) GCACTTTTTCTTAGGCACATTAAAAGGCAATATTTACACATTTCCAGTTCATAGTGTGATATCTTTGGCATTACCATTCC
SPM-A28C  (2723) GCACTTTTTCTTAGGCACATTAAAAGGCAATATTTACACATTTCCAGTTCATAGTGTGATATCTTTGGCATTACCATTCC

Hz_ABCC2(106502) ATGTTGATAGAACAGAATTTGCTTATTTTTGTTTTTTTTTTTCTTCCCAGGTGATTTGATCCCGGTGGGAAGTGTCGGTC
WT        (7490) ATGTTGATAGAACAGAATTTGCTTATTTTTGTTTTTTT----CTTCCCAGGTGATTTGATCCCGGTGGGAAGTGTCGGTC
SPM-A28C  (2803) ATGTTGATAGAACAGAATTTGCTTATTTTTGTTTTTTTTT--CTTCCCAGGTGATTTGATCCCGGTGGGAAGTGTCGGTC

Hz_ABCC2(106582) TGGCTGTCAGTCAGTCCATGGTGCTGACCATGATGTTGCAGATGGCAGCTAGGTTCACAGCTGACTTTTTGGGGCAAATG
WT        (7566) TGGCTGTCAGTCAGTCCATGGTGCTGACCATGATGTTGCAGATGGCAGCTAGGTTCACAGCTGACTTTTTGGGGCAAATG
SPM-A28C  (2881) TGGCTGTCAGTCAGTCCATGGTGCTGACCATGATGTTGCAGATGGCAGCTAGGTTCACAGCTGACTTTTTGGGGCAAATG

Hz_ABCC2(106662) ACGGCTGTGGAGAGAGTTCTGGAATACACCAAGCTACCCACGGAGACCAATATGGAGCAAGGACGTAAGTACAATCATTC
WT        (7646) ACGGCTGTGGAGAGAGTTCTGGAATACACCAAGCTACCCACGGAGACCAATATGGAGCAAGGACGTAAGTACAATCATTC
SPM-A28C  (2961) ACGGCTGTGGAGAGAGTTCTGGAATACACCAAGCTACCCACGGAGACCAATATGGAGCAAGGACGTAAGTACAATCATTC

Hz_ABCC2(106742) GTTTTGAGATAGTTAGGTAATCTGACTTTGATTATTAAGTCCTTATCTGTCTAGCCTTTTTCCAACTATGTTCGGTACTT
WT        (7726) GTTTTGAGATAGTTAGGTAATCTGACTTTGATTATTAAGTCCTTATCTGTCTAGCCTTTTTCCAACTATGTTCGGTACTT
SPM-A28C  (3041) GTTTTGAGATAGTTAGGTAATCTGACTTTGATTATTAAGTCCTTATCTGTCTAGCCTTTTTCCAACTATGTTCGGTACTT

Hz_ABCC2(106822) CTTCCAGTCTATGTGATTGGATGCAGTAATGAAAACCAGTTTTTACATAGGCGACTGCCTATCTGAACTTCTAAATCTAG
WT        (7806) CTTCCAGTCTATGTGATTGGATGCAGTAATGAAAACCAGTTTTTACATAGGCGACTGCCTATCTGAACTTCTAAATCTAG
SPM-A28C  (3121) CTTCCAGTCTATGTGATTGGATGCAGTAATGAAAACCAGTTTTTACATAGGCGACTGCCTATCTGAACTTCTAAATCTAG

Hz_ABCC2(106902) TTACCCGGGTAACCCGATATCCCTTTGGTTGTCAAATTTTGGCTCTGGCTTCTGACTACCGGCCAACCAAAACAAGGACG
WT        (7886) TTACC-GGGTAACCCGATATCCCTTTGGTTGTCAAATTTTGGCTCTGGCTTCTGACTACCGGCCAACCAAAACAAGGACG
SPM-A28C  (3201) TTACCCGGGTAACCCGATATCCCTTTGGTTGTCAAATTTTGGCTCTGGCTTCTGACTACCGGCCAACCAAAACAAGGACG

Hz_ABCC2(106982) ATAAGTTATAACATAGATATATTGATTTTCAGCAACTAACCCACCAAAGGAATGGCCCAGTGCTGGTAGAGTGACGTTCT
WT        (7965) ATAAGTTATAACATAGATATATTGATTTTCAGCAACTAACCCACCAAAGGAATGGCCCAGTGCTGGTAGAGTGACGTTCT
SPM-A28C  (3281) ATAAGTTATAACATAGATATATTGATTTTCAGCAACTAACCCACCAAAGGAATGGCCCAGTGCTGGTAGAGTGACGTTCT

Hz_ABCC2(107062) CAAATGTGTACCTGAATTATTCCATGGAAGACCCGCCGGTGCTGAAGGACTTAAACTTTGAAATTCAAAGCGGCTGGAAG
WT        (8045) CAAATGTGTACCTGAATTATTCCATGGAAGACCCGCCGGTGCTGAAGGACTTAAACTTTGAAATTCAAAGCGGCTGGAAG
SPM-A28C  (3361) CAAATGTGTACCTGAATTATTCCATGGAAGACCCGCCGGTGCTGAAGGACTTAAACTTTGAAATTCAAAGCGGCTGGAAG

Hz_ABCC2(107142) GTAAGAGACCTAATAATGTATTTGATTAAAATAAAAGCAATAAAGATATACTTGCCGTGCTACCCTAAACTACCCGTGTT
WT        (8125) GTAAGAGACCTAATAATGTATTTGATTAAAATAAAAGCAATAAAGATATACTTGCCGTGCTACCCTAAACTACCCGTGTT
SPM-A28C  (3441) GTAAGAGACCTAATAATGTATTTGATTAAAATAAAAGCAATAAAGATATACTTGCCGTGCTACCCTAAACTACCCGTGTT

Hz_ABCC2(107222) CCATGTATTATTCAGATAAATTCCATCCAAAGGTTGTCTAGAAGAAATCGCTGTTTGGCGATAAGACGGCCAATTATACT
WT        (8205) CCATGTATTATTCAGATAAATTCCATCCAAAGGTTGTCTAGAAGAAATCGCTGTTTGGCGATAAGACGGCCAATTATACT
SPM-A28C  (3521) CCATGTATTATTCAGATAAATTCCATCCAAAGGTTGTCTAGAAGAAATCGCTGTTTGGCGATAAGACGGCCAATTATACT

Hz_ABCC2(107302) GTTCTTTGTGTCATATGATTATCAAGATGATATGATGAGAAGATTTATAAAGAATAATCTATCTATGTATTTGCTTACTT
WT        (8285) GTTCTTTGTGTCATATGATTATCAAGATGATATGATGAGAAGATTTATAAAGAATAATCTATCTATGTATTTGCTTACTT
SPM-A28C  (3601) GTTCTTTGTGTCATATGATTATCAAGATGATATGATGAGAAGATTTATAAAGAATAATCTATCTATGTATTTGCTTACTT

Hz_ABCC2(107382) AATGTAATGAATGTTGTAGGTTGGAGTTGTAGGCAGAACAGGAGCCGGCAAGTCATCGCTCA------------------
WT        (8365) AATGTAATGAATGTTGTAGGTTGGAGTTGTAGGCAGAACAGGAGCCGGCAAGTCATCGCTCA------------------
SPM-A28C  (3681) AATGTAATGAATGTTGTAGGTTGGAGTTGTAGGCAGAACAGGAGCCGGCAAGTCATCGCTCACGGCTTTGTTCTCTTTGT

Hz_ABCC2(107444) --TCGCGGCTTTGTTCCGGCTTAGTGACATAAGCGGCAGCATCAAAATTGACGGTGTGGACACCGAAGGATTAGCCAAAA
WT        (8427) --TCGCGGCTTTGTTCCGGCTTAGTGACATAAGCGGCAGCATCAAAATTGACGGTGTGGACACCGAAGGATTAGCCAAAA
SPM-A28C  (3761) TCTCGCGGCTTTGTTCCGGCTTAGTGACATAAGCGGCAGCATCAAAATTGACGGTGTGGACACCGAAGGATTAGCCAAAA

Hz_ABCC2(107522) AGGTATCGATGTTAAAAAATACATTCTTTCTAAAAGACTTAGTGAAGTATTATTTATTGTTTAGTTAAATTACTAATAGA
WT        (8505) AGGTATCGATGTTAAAAAATACATTCTTTCTAAAAGACTTAGTGAAGTATTATTTATTGTTTAGTTAAATTACTAATAGA
SPM-A28C  (3841) AGGTATCGATGTTAAAAAATACATTCTTTCTAAAAGACTTAGTGAAGTATTATTTATTGTTTAGTTAAATTACTAATAGA
```

```
Hz_ABCC2(107602)ATTTGTGGATTTGTTGAAAATATATTAACGGCACGAGCTCGTGGTTTCCAGCACCCGGATAAAAATATCCTTTTTATTGC
WT         (8585)ATTTGTGGATTTGTTGAAAATATATTAACGGCACGAGCTCGTGGTTTCCAGCACCCGGATAAAAATATCCTTTTTATTGC
SPM-A28C   (3921)ATTTGTGGATTTGTTGAAAATATATTAACGGCACGAGCTCGTGGTTTCCAGCACCCGGATAAAAATATCCTTTTTATTGC

Hz_ABCC2(107682)AATTTTATTTGCACTCTCTTTTATGAACTCTCTTATGTACTCACCTTAAGTACTCTCTTATGTAAGATCTCATGTACTTC
WT         (8665)AATTTTATTTGCACTCTCTTTTATGAACTCTCTTATGTACTCACCTTAAGTACTCTCTTATGTAAGATCTCATGTACTTC
SPM-A28C   (4001)AATTTTATTTGCACTCTCTTTTATGAACTCTCTTATGTACTCACCTTAAGTACTCTCTTATGTAAGATCTCATGTACTTC

Hz_ABCC2(107762)CCTTATGTACTCTCTTATGTACCCTCTTATGTGCTCTCTTATGTACTCCCCTTATGTACTCCTCTTCTGTACTCCTCTTA
WT         (8745)C--TATGTACTCTCTTATGTACCCTCTTATGTGCTCTCTTATGTACTCCC-TTATGTACTCCTCTTCTGTACTCCTCTTA
SPM-A28C   (4081)CCTTATGTACTCTCTTATGTACCCTCTTATGTGCTCTCTTATGTACTCCCCTTATGTACTCCTCTTCTGTACTCCTCTTA

Hz_ABCC2(107842)TACAAAGTGTGTCGTACCTAATCACATGAAATTAAATACGTTGATATACTGGTTAATACATTTGTAGGAAAAGTAAGAGA
WT         (8822)TACAAAGTGTGTCGTACCTAATCACATGAAATTAAATACGTTGATATACTGGTTAATACATTTGTAGGAAAAGTAAGAGA
SPM-A28C   (4161)TACAAAGTGTGTCGTACCTAATCACATGAAATTAAATACGTTGATATACTGGTTAATACATTTGTAGGAAAAGTAAGAGA

Hz_ABCC2(107922)ATCTTATGTCGTCAATTTTTTTTTATTTAGAAAATTGATTCAGCGATAACTTTTTACATTCGCAGACTTTGAGATCGAAA
WT         (8902)ATCTTATGTCGTCAATTTTTTTTTA-TTTGAAAATTGATTCAGCGATAACTTTTTACATTCGCAGACTTTGAGATCGAAA
SPM-A28C   (4241)ATCTTATGTCGTCAATTTTTTTAT--TTAGAAAATTGATTCAGCGATAACTTTTTACATTCGCAGACTTTGAGATCGAAA

Hz_ABCC2(108002)ATATCAATTATTCCACAAGAGCCGGTGCTGTTCTCGGCTACTCTGCGATACAATTTGGACCCGTTCGACGATTACAGCGA
WT         (8981)ATATCAATTATTCCACAAGAGCCGGTGCTGTTCTCGGCTACTCTGCGATACAATTTGGACCCGTTCGACGATTACAGCGA
SPM-A28C   (4319)ATATCAATTATTCCACAAGAGCCGGTGCTGTTCTCGGCTACTCTGCGATACAATTTGGACCCGTTCGACGATTACAGCGA

Hz_ABCC2(108082)CGACGATATTTGGAGGGCGTTGGAACAGGTACGCTTAATTAAATTACTTCTTAACAGACCCTCGGCACTCGAACTAATCT
WT         (9061)CGACGATATTTGGAGGGCGTTGGAACAGGTACGCTTAATTAAATTACTTCTTAACAGACCCTCGGCACTCGAACTAATCT
SPM-A28C   (4399)CGACGATATTTGGAGGGCGTTGGAACAGGTACGCTTAATTAAATTACTTCTTAACAGACCCTCGGCACTCGAACTAATCT

Hz_ABCC2(108162)AACTAATGAATGAATCTATGATTTTAGGTGGAATTAAAAGAAGGAATACCGGCTTTAGACTTTAAGGTCGCTGAAGGTGG
WT         (9141)AACTAATGAATGAATCTATGATTTTAGGTGGAATTAAAAGAAGGAATACCGGCTTTAGACTTTAAGGTCGCTGAAGGTGG
SPM-A28C   (4479)AACTAATGAATGAATCTATGATTTTAGGTGGAATTAAAAGAAGGAATACCGGCTTTAGACTTTAAGGTCGCTGAAGGTGG

Hz_ABCC2(108242)TACTAACTTCTCTATGGGACAACGTCAGTTGGTTTGCTTGGCTCGTGCCATTCTACGCTCTAACAAAATACTCATCATGG
WT         (9221)TACTAACTTCTCTATGGGACAACGTCAGTTGGTTTGCTTGGCTCGTGCCATTCTACGCTCTAACAAAATACTCATCATGG
SPM-A28C   (4559)TACTAACTTCTCTATGGGACAACGTCAGTTGGTTTGCTTGGCTCGTGCCATTCTACGCTCTAACAAAATACTCATCATGG

Hz_ABCC2(108322)ACGAAGCTACCGCTAACGTCGATCCTCAGTAAGTCAATCATTCTATAGTTGTGGTCAATGGCGTTAATTTATTGTTCTAG
WT         (9301)ACGAAGCTACCGCTAACGTCGATCCTCAGTAAGTCAATCATTCTATAGTTGTGGTCAATGGCGTTAATTTATTGTTCTAG
SPM-A28C   (4639)ACGAAGCTACCGCTAACGTCGATCCTCAGTAAGTCAATCATTCTATAGTTGTGGTCAATGGCGTTAATTTATTGTTCTAG

Hz_ABCC2(108402)GTTTTCCAATGTGTTAAAAGTTTAATTAAAAGCAAAATATAGATACGTCATTAAATAAATTATTTATTCGGTAATAGGAC
WT         (9381)GTTTTCCAATGTGTTAAAAGTTTAATTAAAAGCAAAATATAGATACGTCATTAAATAAATTATTTATTCGGTAATAGGAC
SPM-A28C   (4719)GTTTTCCAATGTGTTAAAAGTTTAATTAAAAGCAAAATATAGATACGTCATTAAATAAATTATTTATTCGGTAATAGGAC

Hz_ABCC2(108482)TTCCTTATTGGCGAGTTTGTTTCATTTTGTTTCTGAATTTAAATACATAATAGTCAGCTAATATAATAATTACTGCAGAG
WT         (9461)TTCCTTATTGGCGAGTTTGTTTCATTTTGTTTCTGAATTTAAATACATAATAGTCAGCTAATATAATAATTACTGCAGAG
SPM-A28C   (4799)TTCCTTATTGGCGAGTTTGTTTCATTTTGTTTCTGAATTTAAATACATAATAGTCAGCTAATATAATAATTACTGCAGAG

Hz_ABCC2(108562)AACTTACACGTATTTTTCTTACTTATGCATTCTTCTTTTACAGGACGGACGCTTTGATCCAAAAGACGATCCGTCGTCAG
WT         (9541)AACTTACACGTATTTTTCTTACTTATGCATTCTTCTTTTACAGGACGGACGCTTTGATCCAAAAGACGATCCGTCGTCAG
SPM-A28C   (4879)AACTTACACGTATTTTTCTTACTTATGCATTCTTCTTTTACAGGACGGACGCTTTGATCCAAAAGACGATCCGTCGTCAG

Hz_ABCC2(108642)TTTGCGTCGTGCACGGTGCTCACCATCGCGCATCGACTGAACACCATCATGGACTCCGACCGAGTGCTGGTCATGGACCA
WT         (9621)TTTGCGTCGTGCACGGTGCTCACCATCGCGCATCGACTGAACACCATCATGGACTCCGACCGAGTGCTGGTCATGGACCA
SPM-A28C   (4959)TTTGCGTCGTGCACGGTGCTCACCATCGCGCATCGACTGAACACCATCATGGACTCCGACCGAGTGCTGGTCATGGACCA

Hz_ABCC2(108722)GGGCGAAGTGGCCGAGTTCGACCATCCCCACATCTTGCTCAGCAACCCCAATAGCAAGTTCTTCTCTATGGTCCGAGAGA
WT         (9701)GGGCGAAGTGGCCGAGTTCGACCATCCCCACATCTTGCTCAGCAACCCCAATAGCAAGTTCTTCTCTATGGTCCGAGAGA
SPM-A28C   (5039)GGGCGAAGTGGCCGAGTTCGACCATCCCCACATCTTGCTCAGCAACCCCAATAGCAAGTTCTTCTCTATGGTCCGAGAGA

Hz_ABCC2(108802)CAGGAGAAAGCATGACAAGGACCTTAATGGAGGTCGCTAAGGC
WT         (9781)CAGGAGAAAGCATGACAAGGACCTTAATGGAGGTCGCTAAGGC
SPM-A28C   (5119)CAGGAGAAAGCATGACAAGGACCTTAATGGAGGTCGCTAAGGC
```

Figure S3. Alignment of *Helicoverpa zea* ATP binding cassette transporter subfamily C2 (*ABCC2*) reference genome sequence from 99,062 to 108,844 bp and amplicon sequences from wild type SIMRU and knockout line SPM-A28C. The nucleotide sequence of SPM-A28C has a 4,645 bp deletion from 99,733 to 104,378 bp (exon 3 to 13) and an insertion at the sgRNA target site in exon 21. Identical nucleotides are shown in white text with black background and alignment gaps are indicated by hyphens. Deletions and insertions caused

by CRISPR/Cas9 editing at sgRNA target sites are marked with red text and nucleotide polymorphisms between the reference genomic scaffold (Accession: KY701524) sequence and amplicon sequences, including those may have been introduced by repair enzymes are shown in dark gray text with white background.

```
Hz_ABCC2  (104174) CAATGATGCGAGGAATATCAAAAATCTCAGTTAAGAGCGACACCGAAATGGAACAGAAGGCTCAAATACAGGAGGCAGAG
 SPM-B1        (1) CAATGATGCGAGGAATATCAAAAATCTCAGTTAAGAGCGACACCGAAATGGAACAGAAGGCTCAAATACAGGAGGCAGAG

Hz_ABCC2  (104254) GAGAGAGCGACAGGTAGCTTGAAGTTTGAGGTGGTGCTCAAGTACCTGAGCTCCGTCCAGTCCTGGTGTCTGGTGTTTAC
 SPM-B1       (81) GAGAGAGCGACAGGTAGCTTGAAGTTTGAGGTGGTGCTCAAGTACCTGAGCTCCGTCCAGTCCTGGTGTCTGGTGTTTAC

Hz_ABCC2  (104334) GGCGTTCCTTGTACTGCTGATCACGCAAGGTGCTGCCACCACTGCCGACTATTGGTTGAGTTTCTGGTAAGATAATGAAT
SPM-B1       (161) GGCGTTCCTTGTACTGCTGATCACGCAAGGTGCTGCCACCACTG------------------------------------

Hz_ABCC2  (104414) TATTTTTCAAAAATTTAATTTAACGGAAACAAAGAACGTATTTTAAATTGGCTTCTCTGAATATGTCTTAAATGTCTCCT
SPM-B1       (205) --------------------------------------------------------------------------------

Hz_ABCC2  (104494) GATAGTTAACATTTTGATTGCAGGACTAACCAAGTGGATTCTTATGAACAATCTTTACCTGAAGGCGTGGATCCAGGTAA
SPM-B1       (205) --------------------------------------------------------------------------------

Hz_ABCC2  (104574) GTCTACGAAATATCTCTGTTTTATTTATATGAACTTCCTGCGTATGTACCATTTGCAGTTCTAAGATCTCAACCTCAACT
SPM-B1       (205) --------------------------------------------------------------------------------

Hz_ABCC2  (104654) AAAAAGTTATTTCTTTTCAGATACTGACATGAACGCACAAATTGGCTTACTTACAACTGCCCAGTACCTATACGTTTTCG
SPM-B1       (205) --------------------------------------------------------------------------------

Hz_ABCC2  (104734) GTGGAGTTATATTGGCTTTAATAGTCATGACCCTCGTCAGAATCACAGCTTTCGTAGCGATGACAATGCGAGCTTCTCAA
SPM-B1       (205) --------------------------------------------------------------------------------

Hz_ABCC2  (104814) AATCTTCACAACACTATTTACGAAAAATTAATTGTGACAGTAATGAGATTTTTCGATACCAATCCTTCTGGTGAGTAACA
SPM-B1       (205) --------------------------------------------------------------------------------

Hz_ABCC2  (104894) TTCATCATTACACATAACATTATTTATTTAAGTGATCACATAAAAAAGTGCCATAGCATTTTGCGAGACAGTCAGTTAGA
SPM-B1       (205) --------------------------------------------------------------------------------

Hz_ABCC2  (104974) TAAGTAGAGATAATGATTTTAAAATCAAAGTAACGGACTCTCTCAATGTTAGAAGTGTAGGTAATTAGCGATAATTGCAG
SPM-B1       (205) --------------------------------------------------------------------------------

Hz_ABCC2  (105054) CTGGCCAAGTGCCCAGGTTGCCGTGAGTAGACCTTAACATTAGTACACGCCAATTGCAACGACGGTAGCCAAGATAGCTT
SPM-B1       (205) --------------------------------------------------------------------------------

Hz_ABCC2  (105134) GGATAATAACAACATCTTTAATGAGTTCTGCAACTTTCGACTTGTGATTTTGGTGGCAGTAGATTTAAGAAAACGTTGTA
SPM-B1       (205) --------------------------------------------------------------------------------

Hz_ABCC2  (105214) CATATGTGTAGTATAAGATGATGATACATTCTAGGCTATCCGTATTTTTTTAGATTATGATTTATTAAATGCAAGAAGAT
SPM-B1       (205) --------------------------------------------------------------------------------

Hz_ABCC2  (105294) GATGCAATGAAATTAATTTACAACATCATGCTTATTGGAGCTTCTTTTGAATTTCGCAGGTCGTGTGCTGAACAGATTCT
SPM-B1       (205) --------------------------------------------------------------------------------

Hz_ABCC2  (105374) CAAAAGACATGGGTGCCATGGATGAGCTTCTTCCTCGCAGTCTTTTAGAAACAGTTCAGATGTATTTGTCGCTGACCAGT
SPM-B1       (205) --------------------------------------------------------------------------------

Hz_ABCC2  (105454) GTGTTGGTGCTGAACGCCACTGCATTGCCCTGGACGCTTATACCTACCTCCGTGTTGATTGTCATCTTCGTGCTCATGTT
SPM-B1       (205) --------------------------------------------------------------------------------

Hz_ABCC2  (105534) GAGATGGTACCTGAATACAGCTCAGGCTGTCAAACGTTTGGAGGGCACAAGTAAGTGTTACATGAACCATAGGCGTTATT
SPM-B1       (205) --------------------------------------------------------------------------------

Hz_ABCC2  (105614) TAAGAACAAATATTGATTTAGAGTGTAAATTAATTAACGGTTAGAACATCGCTAATAGTTTTGACTGCCTCGTTGGTCTA
SPM-B1       (205) --------------------------------------------------------------------------------

Hz_ABCC2  (105694) GAGGTCGCAAGCGCGGCTGCTGTGCTCGAGGTCTCGGGTTCGATTCCCGGGTCAGGCCGAAATCGCTTTGTGGGTTTTCA
SPM-B1       (205) --------------------------------------------------------------------------------

Hz_ABCC2  (105774) TAAACTTTCACAAAGCAGCCCGTAGCCTGGAAGTTGGTGATTGATTCACCCGTGCATCGGAGAGCACGTAAATGTCGGTC
SPM-B1       (205) --------------------------------------------------------------------------------

Hz_ABCC2  (105854) CTGCGCCTGATCTCTTTCCGGTCGTGTCGGATTGCCGTCCCATCGGGTTATGAGAGTGAAGGAATAGGGAGTGCACCTGT
SPM-B1       (205) --------------------------------------------------------------------------------

Hz_ABCC2  (105934) GTCTGCGCAAATGCTCGTGCACTATAATATGTCCTGCGCAGCTGGCTGATCTCCTTTAATGAGAACAGCCGCCGTGGCCG
SPM-B1       (205) --------------------------------------------------------------------------------
```

```
Hz_ABCC2  (106014)AAATCGGCCGTGGACGCCATTATTAATAGTTTTGAAATAAAATTATTTTAATTTTGTTTTATTTCTACTTACAGCCAAGA
SPM-B1       (205)--------------------------------------------------------------------------------

Hz_ABCC2  (106094)GTCCTGTATTTGGAATGATTAACTCCACTATTTCTGGACTCTCCACTATTAGAAGTTCGGGTTCCCAGGATAGACAGATG
SPM-B1       (205)--------------------------------------------------------------------------------

Hz_ABCC2  (106174)AAATTGTTTGACGAAGCGCAGGTAAACATGAAGCTTATTATTTTTTTATATTAATTAAAATTACATCTAAAAAGATTTAT
SPM-B1       (205)--------------------------------------------------------------------------------

Hz_ABCC2  (106254)CCAATAGTAATGGTTATATATTTCTTATTGCTGTTTACAGAATCTCCACACAAGTGCTTTCCACACATTCTTCGGCGGTT
SPM-B1       (205)CCAATTGTAATGGTTATATATTTCTTATTGCTGTTTACAGAATCTCCACACAAGTGCTTTCCACACATTCTTGGGCGGTT

Hz_ABCC2  (106334)CTACGGCATTTGCATTGTATCTCGATACTTTGTGTTTGACCTACCTCGGTGTCGTCATGTCAATTTTCATTTTGGGCGAC
SPM-B1       (285)TTACGGCATTTGCATTGTATCTCGATACTTTGTGTTTGACCTACCTCGGTGTCGTCATGTCAATTTTCATTTTGGGCGAC

Hz_ABCC2  (106414)TTTGGTGAGCACTTTTTCTTAGGCACATTAAAAGGCAATATTTACACATTTCCAGTTCATAGTGTGATATCTTTGGCATT
SPM-B1       (365)TTTGGTGAGCACTTTTTCTTTGGCACATTAAAAGACAATATTTACACATTTCTAGTTCATAGTGTAATATCTTTGGCATT

Hz_ABCC2  (106494)ACCATTCCATGTTGATAGAACAGAATTTGCTTATTTTTGTTTTTTTTTTTCTTCCCAGGTGATTTGATCCCGGTGGGAAG
SPM-B1       (445)ACCATTCCATGTTGATAGAACAGAATTTGCTTATTTTTGTTTTTTTTTTT-CTTCCCAGGTGTTTTGATCCCGGTGGGAAG

Hz_ABCC2  (106574)TGTCGGTCTGGCTGTCAGTCAGTCCATGGTGCTGACCATGATGTTGCAGATGGCAGCTAGGTTCACAGCTGACTTTTTGG
SPM-B1       (524)TGTCGGTCTGGCTGTCAGTCAGTCCATGGTGCTGACCATGATGTTGCAGATGGCATTTAGGTTCACAGCTGACTTTTTGG

Hz_ABCC2  (106654)GGCAAATGACGGCTGTGGAGAGAGTTCTGGAATACACCAAGCTACCCACGGAGACCAATATGGAGCAAGGACGTAAGTAC
SPM-B1       (604)GGCAAATGACGGCTGTGGAGAGAGTTCTGGAAAACACCCAGCTACCCACGG----------GGAGCAAGGACGTAAGTAC

Hz_ABCC2  (106734)AATCATTCGTTTTGAGATAGTTAGGTAATCTGACTTTGATTATTAAGTCCTTATCTGTCTAGCCTTTTTCCAACTATGTT
SPM-B1       (674)AATCATTCGTTTTGAGATAGTTAGGTAATCTGACTTTGATTATTAAGTCCTTATCTGTCTAGCCTTTTTCCAACTATGTT

Hz_ABCC2  (106814)CGGTACTTCTTCCAGTCTATGTGATTGGATGCAGTAATGAAAACCAGTTTTTACATAGGCGACTGCCTATCTGAACTTCT
SPM-B1       (754)CGGTACTTCTTCCAGTCTATGTGATTGGATGCAGTAATGAAAACCAGTTTTTACATAGGCGACTGCCTATCTGAACTTCT

Hz_ABCC2  (106894)AAATCTAGTTACCCGGGTAACCCGATATCCCTTTGGTTGTCAAATTTTGGCTCTGGCTTCTGACTACCGGCCAACCAAAA
SPM-B1       (834)AAATCTAGTTACCCGGGTAACCCGATATCCCTTTGGTTGTCAAATTTTGGCTCTGGCTTCTGACTACCGGCCAACCAAAA

Hz_ABCC2  (106974)CAAGGACGATAAGTTATAACATAGATATATTGATTTTCAGCAACTAACCCACCAAAGGAATGGCCCAGTGCTGGTAGAGT
SPM-B1       (914)CAAGGACGATAAGTTATAACATAGATATATTGATTTTCAGCAACTAACCCACCAAAGGAATGGCCCAGTGCTGGTAGAGT

Hz_ABCC2  (107054)GACGTTCTCAAATGTGTACCTGAATTATTCCATGGAAGACCCGCCGGTGCTGAAGGACTTAAACTTTGAAATTCAAAGCG
SPM-B1       (994)GACGTTCTCAAATGTGTACCTGAATTATTCCATGGAAGACCCGCCGGTGCTGAAGGACTTAAACTTTGAAATTCAAAGCG

Hz_ABCC2  (107134)GCTGGAAGGTAAGAGACCTAATAATGTATTTGATTAAAATAAAAGCAATAAAGATATACTTGCCGTGCTACCCTAAACTA
SPM-B1      (1074)GCTGGAAGGTAAGAGACCTAATAATGTATTTGATTAAAATAAAAGCAATAAAGATATACTTGCCGTGCTACCCTAAACTA

Hz_ABCC2  (107214)CCCGTGTTCCATGTATTATTCAGATAAATTCCATCCAAAGGTTGTCTAGAAGAAATCGCTGTTTGGCGATAAGACGGCCA
SPM-B1      (1154)CCCGTGTTCCATGTATTATTCAGATAAATTCCATCCAAAGGTTGTCTAGAAGAAATCGCTGTTTGGCGATAAGACGGCCA

Hz_ABCC2  (107294)ATTATACTGTTCTTTGTGTCATATGATTATCAAGATGATATGATGAGAAGATTTATAAAGAATAATCTATCTATGTATTT
SPM-B1      (1234)ATTATACTGTTCTTTGTGTCATATGATTATCAAGATGATATGATGAGAAGATTTATAAAGAATAATCTATCTATGTATTT

Hz_ABCC2  (107374)GCTTACTTAATGTAATGAATGTTGTAGGTTGGAGTTGTAGGCAGAACAGGAGCCGGCAAGTCATCGCTCATCGCGGCTTT
SPM-B1      (1314)GCTTACTTAATGTAATGAATGTTGTAGGTTGGAGTTGTAGGCAGAACAGGAGCCGGCAAGTCATCGCTCATCGCGGCTTT

Hz_ABCC2  (107454)GTTCCGGCTTAGTGACATAAGCGGCAGCATCAAAATTGACGGTGTGGACACCGAAGGATTAGCCAAAAAGGTATCGATGT
SPM-B1      (1394)GTTCCGGCTTAGTGACATAAGCGGCAGCATCAAAATTGACGGTGTGGACACCGAAGGATTAGCCAAAAAGGTATCGATGT

Hz_ABCC2  (107534)TAAAAAATACATTCTTTCTAAAAGACTTAGTGAAGTATTATTTATTGTTTAGTTAAATTACTAATAGAATTTGTGGATTT
SPM-B1      (1474)TAAAAAATACATTCTTTCTAAAAGACTTAGTGAAGTATTATTTATTGTTTAGTTAAATTACTAATAGAATTTGTGGATTT

Hz_ABCC2  (107614)GTTGAAAATATATTAACGGCACGAGCTCGTGGTTTCCAGCACCCGGATAAAAATATCCTTTTTATTGCAATTTTATTTGC
SPM-B1      (1554)GTTGAAAATATATTAACGGCACGAGCTCGTGGTTTCCAGCACCCGGATAAAAATATCCTTTTTATTGCAATTTTATTTGC

Hz_ABCC2  (107694)ACTCTCTTTTATGAACTCTCTTATGTACTCACCTTAAGTACTCTCTTATGTAAGATCTCATGTACTTCCCTTATGTACTC
SPM-B1      (1634)ACTCTCTTTTATGAACTCTCTTATGTACTCACCTTAAGTACTCTCTTATGTAAGATCTCATGTACTTCCCTTATGTACTC

Hz_ABCC2  (107774)TCTTATGTACCCTCTTATGTGCTCTCTTATGTACTCCCCTTATGTACTCCTCTTCTGTACTCCTCTTATACAAAGTGTGT
SPM-B1      (1714)TCTTACGTACCCTCTTATGTGCTCTCTTATGTACTCCCCTTATGTACTCCTCTTCTGTACTCCTCTTATACAAAGTGTGT

Hz_ABCC2  (107854)CGTACCTAATCACATGAAATTAAATACGTTGATATACTGGTTAATACATTTGTAGGAAAAGTAAGAGAATCTTATGTCGT
SPM-B1      (1794)CGTACCTAATCACATGAAATTAAATACGTTGATATACTGGTTAATACATTTGTAGGAAAAGTAAGAGAATCTTATGTCGT
```

```
Hz_ABCC2 (107934)CAATTTTTTTTTATTTAGAAAATTGATTCAGCGATAACTTTTTACATTCGCAGACTTTGAGATCGAAAATATCAATTATT
SPM-B1     (1874)CAATTTTTTTTTATTTAGAACATTGATTCAGCGATAACTTTTTACATTCGCAGACTTTGAGATCGAAAATATCAATTATT

Hz_ABCC2 (108014)CCACAAGAGCCGGTGCTGTTCTCGGCTACTCTGCGATACAATTTGGACCCGTTCGACGATTACAGCGACGACGATATTTG
SPM-B1     (1954)CCACAAGAGCCGGTGCTGTTCTCGGCTACTCTGCGATACAATTTGGACCCGTTCGACGATTACAGCGACGACGATATTTG

Hz_ABCC2 (108094)GAGGGCGTTGGAACAGGTACGCTTAATTAAATTACTTCTTAACAGACCCTCGGCACTCGAACTAATCTAACTAATGAATG
SPM-B1     (2034)GAGGGCGTTGGAACAGGTACGCTTAATTAAATTACTTCTCAACAGACCCTCGGCACTCGAACTAATCTAACTAATGAATG

Hz_ABCC2 (108174)AATCTATGATTTTAGGTGGAATTAAAAGAAGGAATACCGGCTTTAGACTTTAAGGTCGCTGAAGGTGGTACTAACTTCTC
SPM-B1     (2114)AATCTATGATTTTAGGTGGAATTAAAAGAAGGAATACCGGCTTTAGACTTTAAGGTCGCTGAAGGTGGTACTAACTTCTC

Hz_ABCC2 (108254)TATGGGACAACGTCAGTTGGTTTGCTTGGCTCGTGCCATTCTACGCTCTAACAAAATACTCATCATGGACGAAGCTACCG
SPM-B1     (2194)TATGGGACAACGTCAGTTGGTTTGCTTGGCTCGTGCCATTCTACGCTCTAACAAAATACTCATCATGGACGAAGCTACCG

Hz_ABCC2 (108334)CTAACGTCGATCCTCAGTAAGTCAATCATTCTATAGTTGTGGTCAATGGCGTTAATTTATTGTTCTAGGTTTTCCAATGT
SPM-B1     (2274)CTAACGTCGATCCTCAGTAAGTCAATCATTCTATAGTTGTGGTCAATGGCGTTAATTTATTGTTCTAGGTTTTCCAATGT

Hz_ABCC2 (108414)GTTAAAAGTTTAATTAAAAGCAAAATATAGATACGTCATTAAATAAATTATTTATTCGGTAATAGGACTTCCTTATTGGC
SPM-B1     (2354)GTTAAAAGTTTAATTAAAAGCAAAATATAGATACGTCATTAAATAAATTATTTATTCGGTAATAGGACTTCCTTATTGGC

Hz_ABCC2 (108494)GAGTTTGTTTCATTTTGTTTCTGAATTTAAATACATAATAGTCAGCTAATATAATAATTACTGCAGAGAACTTACACGTA
SPM-B1     (2434)GAGTTTGTTTCATTTTGTTTCTGAATTTAAATACATAATAGTCAGCTAATATAATAATTACTGCAGAGAACTTACACGTA

Hz_ABCC2 (108574)TTTTTCTTACTTATGCATTCTTCTTTTACAGGACGGACGCTTTGATCCAAAAGACGATCCGTCGTCAGTTTGCGTCGTGC
SPM-B1     (2514)TTTTTCTTACTTATGCATTCTTCTTTTACAGGACGGACGCTTTGATCCAAAAGACGATCCGTCGTCAGTTTGCGTCGTGC

Hz_ABCC2 (108654)ACGGTGCTCACCATCGCGCATCGACTGAACACCATCATGGACTCCGACCGAGTGCTGGTCATGGACCAGGGCGAAGTGGC
SPM-B1     (2594)ACGGTGCTCACCATCGCGCATCGACTGAACACCATCATGGACTCCGACCGAGTGCTGGTCATGGACCAGGGCGAAGTGGC

Hz_ABCC2 (108734)CGAGTTCGACCATCCCCACATCTTGCTCAGCAACCCCAATAGCAAGTTCTTCTCTATGGTCCGAGAGACAGGAGAAAGCA
SPM-B1     (2674)CGAGTTCGACCATCCCCACATCTTGCTCAGCAACCCCAATAGCAAGTTCTTCTCTATGGTCCGAGAGACAGGAGAAAGCA

Hz_ABCC2 (108814)TGACAAGGA
SPM-B1     (2754)TGACAAGGA
```

Figure S4. Alignment of *Helicoverpa zea* ATP binding cassette transporter subfamily C2 (*ABCC2*) reference genome sequence from 104,174 to 108,822 bp and amplicon sequence from the knockout line SPM-B1 with 1,876 bp and 10 bp deletions at the sgRNA target sites of exon 13 and exon 19, respectively. Identical nucleotides are shown in white text with black background and alignment gaps are indicated by hyphens. Deletions and insertions caused by CRISPR/Cas9 editing at sgRNA target sites are marked with red text and nucleotide polymorphisms between the reference genomic scaffold (Accession: KY701524) sequence and amplicon sequences, including those may have been introduced by repair enzymes are shown in dark gray text with white background.

```
KM360184    (1) ATGGGCGTAGAAAATAAGAATAATGTACAAAATGCGGAAGGTCCGGCCCGCAAGACTTACAAAAAACCGAACATTTTATC
SPM-8       (1) --------------------------------------------------------------------------------
SPM-16      (1) --------------------------------------------------------------------------------
SPM-B1      (1) --------------------------------------------------------------------------------
SPM-A28C    (1) --------------------------------------------------------------------------------

KM360184   (81) CCGTATATTTCTCTGGTGGATGTGTCCTGTGCTTATAACACACTTTAACAAAAGAAATGTAGAAGAATCAGATCTTATAC
SPM-8       (1) --------------------------------------------------------------------------------
SPM-16      (1) --------------------------------------------------------------------------------
SPM-B1      (1) --------------------------------------------------------------------------------
SPM-A28C    (1) --------------------------------------------------------------------------------

KM360184  (161) CGCCCAGTAATTTATATAATTCAGAAAGACAAGGAGAGTATCTTGAAAGATACTGGTTGGCAGAGATAGAAAATGCAACA
SPM-8       (1) --------------------------------------------------------------------------------
SPM-16      (1) --------------------------------------------------------------------------------
SPM-B1      (1) --------------------------------------------------------------------------------
SPM-A28C    (1) --------------------------------------------------------------------------------

KM360184  (241) ATTGAAAATCGGGAGCCGTCACTATGGAAGGCATTACGAAAGGCCTACTGGGTCTCCTACATGCCAGGAGCTATTTTTAT
SPM-8       (1) ------------------------TGGAAGGCATTACGAAAGGCCTACTGGGTCTCCTACATGCCAGGAGCTATTTTTAT
SPM-16      (1) ------------------------TGGAAGGCATTACGAAAGGCCTACTGGGTCTCCTACATGCCAGGAGCTATTTTTAT
SPM-B1      (1) ------------------------TGGAAGGCATTACGAAAGGCCTACTGGGTCTCCTACATGCCAGGAGCTATTTTTAT
SPM-A28C    (1) ------------------------TGGAAGGCATTACGAAAGGCCTACTGGGTCTCCTACATGCCAGGAGCTATTTTTAT

KM360184  (321) CATCATTCAATCTGCAGCCAGGACGTATCAGCCGCTGTTGTTTTCTCAGCTACTGTCGTACTGGTCGGTGGACAGTGAAA
SPM-8      (57) CATCATTCAATCTGCAGCCAGGACGTATCAGCCGCTGTTGTTTTCTCAGTTACTGTCGTACTGGTCGGTGGACAGTGAAA
SPM-16     (57) CATCATTCAATCTGCAGCCAGGACGTATCAGCCGCTGTTGTTTTCTCAGTTACTGTCGTACTGGTCGGTGGACAGTGAAA
SPM-B1     (57) CATCATTCAATCTGCAGCCAGGACGTATCAGCCGCTGTTGTTTTCTCAGTTACTGTCGTACTGGTCGGTGGACAGTGAAA
SPM-A28C   (57) CATCATTCAATCTGCAGCCAGGACGTATCAGCCGCTGTTGTTTTCTCAGTTACTGTCGTACTGATACG------------

KM360184  (401) TGACTCAGCAAGACGCTGGCCTGTATGCTCTCGCCATGCTGGGACTGAACTTCGTCTCCATGATGTGTCAGCACCACAAC
SPM-8_mRNA(137) TGACTCAGCAAGACGCTGGCCTCTATGCTCTCGCCATGCTGGGACTGAACTTCGTCTCCATGATGTGTCAGCACCACAAC
SPM-16    (137) TGACTCAGCAAGACGCTGGCCTCTATGCTCTCGCCATGCTGGGACTGAACTTCGTCTCCATGATGTGTCAGCACCACAAC
SPM-B1    (137) TGACTCAGCAAGACGCTGGCCTCTATGCTCTCGCCATGCTGGGACTGAACTTCGTCTCCATGATGTGTCAGCACCACAAC
SPM-A28C  (125) --------------------------------------------------------------------------------

KM360184  (481) ACACTGTTTGTGATGCGGTTCAGTTTAAAAGTCAAGGTTGCCTGTTCTTCGCTTTTGTATAGGAAGTTGCTCCGCATGAC
SPM-8_mRNA(217) ACACTGTTTGTGATGCGGTTCAGTTTAAAAGTCAAGGTTGCCTGTTCTTCGCTTTTGTATAGGAAGTTGCTCCGCATGAC
SPM-16    (217) ACACTGTTTGTGATGCGGTTCAGTTTAAAAGTCAAGGTTGCCTGTTCTTCGCTTTTGTATAGGAAGTTGCTCCGCATGAC
SPM-B1    (217) ACACTGTTTGTGATGCGGTTCAGTTTAAAAGTCAAGGTTGCCTGTTCTTCGCTTTTGTATAGGAAGTTGCTCCGCATGAC
SPM-A28C  (125) --------------------------------------------------------------------------------

KM360184  (561) CCAAGTGTCGGTGGGTGAGGTGGCAGGAGGAAAGCTGGTGAACTTGCTGTCCAACGATATCACGAGGTTCGACTACGCGT
SPM-8_mRNA(297) CCAAGTGTCGGTGGGTGAGGTGGCAGGAGGAAAGCTGGTGAACTTGCTGTCCAACGATATCACGAGGTTCGACTACGCGT
SPM-16    (297) CCAAGTGTCGGTGGGTGAGGTGGCAGGAGGAAAGCTGGTGAACTTGCTGTCCAACGATATCACGAGGTTCGACTACGCGT
SPM-B1    (297) CCAAGTGTCGGTGGGTGAGGTGGCAGGAGGAAAGCTGGTGAACTTGCTGTCCAACGATATCACGAGGTTCGACTACGCGT
SPM-A28C  (125) --------------------------------------------------------------------------------

KM360184  (641) TCATGTTCCTACATTACTTGTGGATCGTGCCTATCCAAGTGGCTGTTGTTTTGTACTTCTTGTGGGAGGCTGCTGGCTTC
SPM-8_mRNA(377) TCATGTTCCTACATTACTTGTGGATCGTGCCTATCCAAGTGGCTGTTGTTTTGTATTTCTTGTGGGAGGCTGCTGGCTTC
SPM-16    (377) TCATGTTCCTACATTACTTGTGGATCGTGCCTATCCAAGTGGCTGTTGTTTTGTATTTCTTGTGGGAGGCTGCTGGCTTC
SPM-B1    (377) TCATGTTCCTACATTACTTGTGGATCGTGCCTATCCAAGTGGCTGTTGTTTTGTATTTCTTGTGGGAGGCTGCTGGCTTC
SPM-A28C  (125) --------------------------------------------------------------------------------

KM360184  (721) GCACCCTTCGTCGGTCTGTTTGGAGTCGTTATACTGATTTTACCACTGCAAGCTGGCTTGACGAAACTCACAACTGTTGT
SPM-8_mRNA(457) GCACCCTTCGTCGGTCTGTTTGGAGTCGTTATACTGATTTTACCACTGCAAGCTGGCTTGACGAAACTCACAACTGTTGT
SPM-16    (457) GCACCCTTCGTCGGTCTGTTTGGAGTCGTTATACTGATTTTACCACTGCAAGCTGGCTTGACGAAACTCACAACTGTTGT
SPM-B1    (457) GCACCCTTCGTCGGTCTGTTTGGAGTCGTTATACTGATTTTACCACTGCAAGCTGGCTTGACGAAACTCACAACTGTTGT
SPM-A28C  (125) --------------------------------------------------------------------------------

KM360184  (801) AAGACGTGAGACGGCTAAGAGAACGGACAGGCGAATTAAACTAATGAGTGAAATTATTGGTGGTATTCAGGTCATTAAAA
SPM-8_mRNA(537) AAGACGTGAGACGGCTAAGAGAACGGACAGGCGAATTAAACTAATGAGTGAAATTATTGGTGGTATTCAGGTCATTAAAA
SPM-16    (537) AAGACGTGAGACGGCTAAGAGAACGGACAGGCGAATTAAACTAATGAGTGAAATTATTGGTGGTATTCAGGTCATTAAAA
SPM-B1    (537) AAGACGTGAGACGGCTAAGAGAACGGACAGGCGAATTAAACTAATGAGTGAAATTATTGGTGGTATTCAGGTCATTAAAA
SPM-A28C  (125) --------------------------------------------------------------------------------

KM360184  (881) TGTACGCTTGGGAGAAACCCTTCCAGCTAGTTGTGAAGGCAGCTCGTGCCTTTGAAATGAGTGCCCTCAGGAAGTCCATC
SPM-8_mRNA(617) TGTACGCTTGGGAGAAACCCTTCCAGCTAGTTGTGAAGGCAGCTCGTGCCTTTGAAATGAGTGCCCTCAGGAAGTCCATC
SPM-16    (617) TGTACGCTTGGGAGAAACCCTTCCAGCTAGTTGTGAAGGCAGCTCGTGCCTTTGAAATGAGTGCCCTCAGGAAGTCCATC
SPM-B1    (617) TGTACGCTTGGGAGAAACCCTTCCAGCTAGTTGTGAAGGCAGCTCGTGCCTTTGAAATGAGTGCCCTCAGGAAGTCCATC
SPM-A28C  (125) --------------------------------------------------------------------------------
```

```
KM360184  (961) TTCATCAGGAGTACTTTCCTAGGGTTCATGTTGTTCACTGAGCGAAGCATCATGTTTGTCACAGTGTTGACACTCGCTCT
SPM-8_mRNA(697) TTCATCAGGAGTACTTTCCTAGGGTTCATGTTGTTCACTGAGCGAAGCATCATGTTTGTCACAGTGTTGACACTCGCTCT
SPM-16    (697) TTCATCAGGAGTACTTTCCTAGGGTTCATGTTGTTCACTGAGCGAAGCATCATGTTTGTCACAGTGTTGACACTCGCTCT
SPM-B1    (697) TTCATCAGGAGTACTTTCCTAGGGTTCATGTTGTTCACTGAGCGAAGCATCATGTTTGTCACAGTGTTGACACTCGCTCT
SPM-A28C  (125) --------------------------------------------------------------------------------

KM360184 (1041) CACAGGCACTATGATTACTGCCACTACGATATACCCTATTCAACAGTACTTCAGTATTATTCAGTTCAATGTAACACTGA
SPM-8_mRNA(777) CACAGGCACTATGATTACTGCCACTACGATATACCCTATTCAACAGTACTTCAGTATTATTCAGTTTAACGTAACACTGA
SPM-16    (777) CACAGGCACTATGATTACTGCCACTACGATATACCCTATTCAACAGTACTTCAGTATTATTCAGTTTAACGTAACACTGA
SPM-B1    (777) CACAGGCACTATGATTACTGCCACTACGATATACCCTATTCAACAGTACTTCAGTATTATTCAGTTTAACGTAACACTGA
SPM-A28C  (125) --------------------------------------------------------------------------------

KM360184 (1121) TCATTCCTATGGCAATCGCAAGTTATTCCGAGATGATGGTGTCCATAGAACGTATCCAGGGATTCCTTAGTTTGGACGAG
SPM-8_mRNA(857) TCATTCCTATGGCAATCGCAAGTTATTCCGAGATGATGGTGTCCATAGAACGTATCCAGGGATTCCTTAGTTTGGACGAG
SPM-16    (857) TCATTCCTATGGCAATCGCAAGTTATTCCGAGATGATGGTGTCCATAGAACGTATCCAGGGATTCCTTAGTTTGGACGAG
SPM-B1    (857) TCATTCCTATGGCAATCGCAAGTTATTCCGAGATGATGGTGTCCATAGAACGTATCCAGGGATTCCTTAGTTTGGACGAG
SPM-A28C  (125) --------------------------------------------------------------------------------

KM360184 (1201) CGGTCCGACATGCAAGTGACTCCAAAAATGAATGGATCTAACAATAACACTTTGTTTAAATCCAAGAAGTCACCACTTGA
SPM-8     (937) CGGTCCGACATGCAAGTGACTCCAAAAATTAATGGATCTAACAATAACACTTTGTTTAAATCCAAGAAGTCACCACTTGA
SPM-16    (937) CGGTCCGACATGCAAGTGACTCCAAAAATTAATGGATCTAACAATAACACTTTGTTTAAATCCAAGAAGTCACCACTTGA
SPM-B1    (937) CGGTCCGACATGCAAGTGACTCCAAAAATTAATGGATCTAACAATAACACTTTGTTTAAATCCAAGAAGTCACCACTTGA
SPM-A28C  (125) --------------------------------------------------------------------------------

KM360184 (1281) AGTAGGCATCGTGCCTAAGAAATACTCACCTAGTGAAGTTATGGCTGCAAAGGAGATGCAGGATGATCCTACCCAGATGG
SPM-8    (1017) AGTAGGCATCGTGCCTAAGAAATACTCACCTAGTGAAGTTATGGCTGCAAAGGAGATGCAGGATGATCCTACCCAGATGG
SPM-16   (1017) AGTAGGCATCGTGCCTAAGAAATACTCACCTAGTGAAGTTATGGCTGCAAAGGAGATGCAGGATGATCCTACCCAGATGG
SPM-B1   (1017) AGTAGGCATCGTGCCTAAGAAATACTCACCTAGTGAAGTTATGGCTGCAAAGGAGATGCAGGATGATCCTACCCAGATGG
SPM-A28C  (125) --------------------------------------------------------------------------------

KM360184 (1361) ACTATCCTATCAGGCTTAACAAAGTAAGCGCATCGTGGACCGGCAGCAATAGTTCTTCAGAAATGACACTTAAGAATATA
SPM-8    (1097) ACTATCCTATCAGGCTTAACAAAGTAAGCGCATCGTGGACCGGCAGCAATAGTTCTTCAGAAATGACACTTAAGAATATA
SPM-16   (1097) ACTATCCTATCAGGCTTAACAAAGTAAGCGCATCGTGGACCGGCAGCAATAGTTCTTCAGAAATGACACTTAAGAATATA
SPM-B1   (1097) ACTATCCTATCAGGCTTAACAAAGTAAGCGCATCGTGGACCGGCAGCAATAGTTCTTCAGAAATGACACTTAAGAATATA
SPM-A28C  (125) --------------------------------------------------------------------------------

KM360184 (1441) TCGTTACGTATACGTAAAGGAAAATTGTGTGCTATCATTGGTCCTGTGGGGTCCGGAAAGACATCTCTACTGCAACTGCT
SPM-8    (1177) TCGTTACGTATTCGTAAAGGAAAATTGTGTGCTATCATTGGTCCTGTGGGGTCCGGAAAGACATCTCTACTGCAACTGCT
SPM-16   (1177) TCGTTACGTATTCGTAAAGGAAAATTGTGTGCTATCATTGGTCCTGTGGGGTCCGGAAAGACATCTCTACTGCAACTGCT
SPM-B1   (1177) TCGTTACGTATTCGTAAAGGAAAATTGTGTGCTATCATTGGTCCTGTGGGGTCCGGAAAGACATCTCTACTGCAACTGCT
SPM-A28C  (125) --------------------------------------------------------------------------------

KM360184 (1521) CTTAAAAGAATTACCATTGAATAGTGGTACACTTGACGTGAGCGGGAAAATGTCGTACGCTTGTCAAGAGTCTTGGCTGT
SPM-8    (1257) CTTAAAAGAATTACCATTGAATAGTGGTACACTTGACGTGAGCGGGAAAATGTCGTACGCTTGTCAAGAGTCCTGGCTGT
SPM-16   (1257) CTTAAAAGAATTACCATTGAATAGTGGTACACTTGACGTGAGCGGGAAAATGTCGTACGCTTGTCAAGAGTCCTGGCTGT
SPM-B1   (1257) CTTAAAAGAATTACCATTGAATAGTGGTACACTTGACGTGAGCGGGAAAATGTCGTACGCTTGTCAAGAGTCCTGGCTGT
SPM-A28C  (125) --------------------------------------------------------------------------------

KM360184 (1601) TCCCAGGCACAGTACGAGAAAACATTTTGTTCGGCCTAACTTACGAACCCACAAAATACAAAGAGGTTTGCAAGGTGTGT
SPM-8    (1337) TCCCAGGCACAGTACGAGAAAACATTTTGTTCGGCCTAACTTACGAACCCACAAAATACAAAGAGGTTTGCAAGGTGTGT
SPM-16   (1337) TCCCAGGCACAGTACGAGAAAACATTTTGTTCGGCCTAACTTACGAACCCACAAAATACAAAGAGGTTTGCAAGGTGTGT
SPM-B1   (1337) TCCCAGGCACAGTACGAGAAAACATTTTGTTCGGCCTAACTTACGAACCCACAAAATACAAAGAGGTTTGCAAGGTGTGT
SPM-A28C  (125) --------------------------------------------------------------------------------
                      1681
1760
KM360184 (1681) TCGTTGCTGCCTGACTTCAAGCAGTTCCCGTATGGTGACCTGTCTTTAGTGGGAGAACGAGGAGTGTCCCTGTCAGGTGG
SPM-8    (1417) TCGTTGCTGCCTGACTTCAAGCAGTTCCCGTATGGTGACCTGTCTTTAGTGGGAGAACGAGGAGTGTCCCTGTCAGGTGG
SPM-16   (1417) TCGTTGCTGCCTGACTTCAAGCAGTTCCCGTATGGTGACCTGTCTTTAGTGGGAGAACGAGGAGTGTCCCTGTCAGGTGG
SPM-B1   (1417) TCGTTGCTGCCTGACTTCAAGCAGTTCCCGTATGGTGACCTGTCTTTAGTGGGAGAACGAGGAGTGTCCCTGTCAGGTGG
SPM-A28C  (125) --------------------------------------------------------------------------------

KM360184 (1761) TCAAAGAGCCAGGATCAATTTGGCTAGAGCAATTTATCGTGAGGCCGACATTTACTTGCTGGATGATCCCCTATCTGCAG
SPM-8    (1497) TCAAAGAGCCAGGATCAATTTGGCTAGAGCAATTTATCGTGAGGCCGACATTTACTTGCTGGATGATCCCCTATCTGCAG
SPM-16   (1497) TCAAAGAGCCAGGATCAATTTGGCTAGAGCAATTTATCGTGAGGCCGACATTTACTTGCTGGATGATCCCCTATCTGCAG
SPM-B1   (1497) TCAAAGAGCCAGGATCAATTTGGCTAGAGCAATTTATCGTGAGGCCGACATTTACTTGCTGGATGATCCCCTATCTGCAG
SPM-A28C  (125) --------------------------------------------------------------------------------

KM360184 (1841) TGGACGCTAATGTAGGCAGACAACTGTTTGATGGCTGCATCAAAGGCTACCTCACTGGACGAACTTGCGTCTTGGTCACC
SPM-8    (1577) TGGACGCTAATGTAGGCAGACAACTGTTTGATGGCTGCATCAAAGGCTACCTCACTGGACGAACTTGCGTCTTGGTCACC
SPM-16   (1577) TGGACGCTAATGTAGGCAGACAACTGTTTGATGGCTGCATCAAAGGCTACCTCACTGGACGAACTTGCGTCTTGGTCACC
```

```
SPM-B1     (1577) TGGACGCTAATGTAGGCAGACAACTGTTTGATGGCTGCATCAAAGGCTACCTCACTGGACGAACTTGCGTCTTGGTCACC
SPM-A28C    (125) --------------------------------------------------------------------------------

KM360184   (1921) CATCAGATCCATTACCTCAAAGCTGCTGATTTTATTGTAGTCCTTAATGAGGGTTCCGTCGAGAATATGGGCACTTATGA
SPM-8      (1657) CATCAGATCCATTACCTCAAAGCTGCTGATTTTATTGTAGTCCTTAATGAGGGTTCCGTCGAGAATATGGGCACTTATGA
SPM-16     (1657) CATCAGATCCATTACCTCAAAGCTGCTGATTTTATTGTAGTCCTTAATGAGGGTTCCGTCGAGAATATGGGCACTTATGA
SPM-B1     (1657) CATCAGATCCATTACCTCAAAGCTGCTGATTTTATTGTAGTCCTTAATGAGGGTTCCGTCGAGAATATGGGCACTTATGA
SPM-A28C    (125) --------------------------------------------------------------------------------

KM360184   (2001) TGAGCTGGTGAAGACAGGAACTGAATTCTCGATGCTGCTATCCAACCAAGAAAATGACGCAACTGAAAACGAAAAGAAAG
SPM-8      (1737) TGAGCTGGTGAAGACAGGAACTGAATTCTCGATGCTGCTATCCAACCAAGAAAATGACGCAACTGAAAACGAAAAGAAAG
SPM-16     (1737) TGAGCTGGTGAAGACAGGAACTGAATTCTCGATGCTGCTATCCAACCAAGAAAATGACGCAACTGAAAACGAAAAGAAAG
SPM-B1     (1737) TGAGCTGGTGAAGACAGGAACTGAATTCTCGATGCTGCTATCCAACCAAGAAAATGACGCAACTGAAAACGAAAAGAAAG
SPM-A28C    (125) --------------------------------------------------------------------------------

KM360184   (2081) ACCGACCAGCAATGATGCGAGGAATATCGAAAATCTCAGTTAAGAGCGACACCGAAATGGAACAGAAGGCTCAAATACAG
SPM-8      (1817) ACCGACCAGCAATGATGCGAGGAATATCAAAAATCTCAGTTAAGAGCGACACCGAAATGGAACAGAAGGCTCAAATACAG
SPM-16     (1817) ACCGACCAGCAATGATGCGAGGAATATCAAAAATCTCAGTTAAGAGCGACACCGAAATGGAACAGAAGGCTCAAATACAG
SPM-B1     (1817) ACCGACCAGCAATGATGCGAGGAATATCAAAAATCTCAGTTAAGAGCGACACCGAAATGGAACAGAAGGCTCAAATACAG
SPM-A28C    (125) --------------------------------------------------------------------------------

KM360184   (2161) GAGGCAGAGGAGAGAGCGACAGGTAGCTTGAAGTTTGAGGTGGTGCTCAAGTACCTGAGCTCCGTCCAGTCCTGGTGTCT
SPM-8      (1897) GAGGCAGAGGAGAGAGCGACAGGTAGCTTGAAGTTTGAGGTGGTGCTCAAGTACCTGAGCTCCGTCCAGTCCTGGTGTCT
SPM-16     (1897) GAGGCAGAGGAGAGAGCGACAGGTAGCTTGAAGTTTGAGGTGGTGCTCAAGTACCTGAGCTCCGTCCAGTCCTGGTGTCT
SPM-B1     (1897) GAGGCAGAGGAGAGAGCGACAGGTAGCTTGAAGTTTGAGGTGGTGCTCAAGTACCTGAGCTCCGTCCAGTCCTGGTGTCT
SPM-A28C    (125) --------------------------------------------------------------------------------

KM360184   (2241) GGTGTTTACGGCGTTCCTTGTACTGCTGATCACGCAAGGTGCTGCCACCACTGCCGACTATTGGTTGAGTTTCTGGACTA
SPM-8      (1977) GGTGTTTACGGCGTTCCTTGTACTGCTGATCACGCAAGGTGCTGCCACCACTGCCGACTATTGGTTGAGTTTCTGGACTA
SPM-16     (1977) GGTGTTTACGGCGTTCCTTGTACTGCTGATCACGCAAGGTGCTGCCACCACTGCCGACTATTGGTTGAGTTTCTGGACTA
SPM-B1     (1977) GGTGTTTACGGCGTTCCTTGTACTGCTGATCACGCAAG------------------------------------------
SPM-A28C    (125) --------------------------------------------------ACACCGACTATTGGTTGAGTTTCTGGACTA

KM360184   (2321) ACCAAGTAGATTCTTATGAACAATCTTTACCTGAAGGCGTGGATCCAGATACTGACATGAACGCACAAATTGGCTTACTT
SPM-8      (2057) ACCAAGTGGATTCTTATGAACAATCTTTACCTGAAGGCGTGGATCCAGATACTGACATGAACGCACAAATTGGCTTACTT
SPM-16     (2057) ACCAAGTGGATTCTTATGAACAATCTTTACCTGAAGGCGTGGATCCAGATACTGACATGAACGCACAAATTGGCTTACTT
SPM-B1     (2018) --------------------------------------------------------------------------------
SPM-A28C    (155) ACCAAGTGGATTCTTATGAACAATCTTTACCTGAAGGCGTGGATCCAGATACTGACATGAACGCACAAATTGGCTTACTT

KM360184   (2401) ACAACTGCTCAGTACCTATACGTTTTCGGTGGAGTTATATTGGCTTTAATAGTCATGACCCTCGTCAGAATCACAGCTTT
SPM-8      (2137) ACAACTGCCCAGTACCTATACGTTTTCGGTGGAGTTATATTGGCTTTAATAGTCATGACCCTCGTCAGAATCACAGCTTT
SPM-16     (2137) ACAACTGCCCAGTACCTATACGTTTTCGGTGGAGTTATATTGGCTTTAATAGTCATGACCCTCGTCAGAATCACAGCTTT
SPM-B1     (2018) --------------------------------------------------------------------------------
SPM-A28C    (235) ACAACTGCCCAGTACCTATACGTTTTCGGTGGAGTTATATTGGCTTTAATAGTCATGACCCTCGTCAGAATCACAGCTTT

KM360184   (2481) CGTAGCGATGACAATGCGAGCTTCTCAAAATCTTCACAACACTATTTACGAAAAATTAATTGTGACAGTAATGAGATTTT
SPM-8      (2217) CGTAGCGATGACAATGCGAGCTTCTCAAAATCTTCACAACACTATTTACGAAAAATTAATTGTGACAGTAATGAGATTTT
SPM-16     (2217) CGTAGCGATGACAATGCGAGCTTCTCAAAATCTTCACAACACTATTTACGAAAAATTAATTGTGACAGTAATGAGATTTT
SPM-B1     (2018) --------------------------------------------------------------------------------
SPM-A28C    (315) CGTAGCGATGACAATGCGAGCTTCTCAAAATCTTCACAACACTATTTACGAAAAATTAATTGTGACAGTAATGAGATTTT

KM360184   (2561) TCGATACCAATCCTTCTGGTCGTGTGCTGAACAGATTCTCAAAAGACATGGGTGCCATGGATGAGCTTCTTCCTCGCAGT
SPM-8      (2297) TCGATACCAATCCTTCTGGTCGTGTGCTGAACAGATTCTCAAAAGACATGGGTGCCATGGATGAGCTTCTTCCTCGCAGT
SPM-16     (2297) TCGATACCAATCCTTCTGGTCGTGTGCTGAACAGATTCTCAAAAGACATGGGTGCCATGGATGAGCTTCTTCCTCGCAGT
SPM-B1     (2018) --------------------------------------------------------------------------------
SPM-A28C    (395) TCGATACCAATCCTTCTGGTCGTGTGCTGAACAGATTCTCAAAAGACATGGGTGCCATGGATGAGCTTCTTCCTCGCAGT

KM360184   (2641) CTTTTAGAAACAGTTCAGATGTATTTGTCGCTGACCAGTGTGTTGGTGCTGAACGCCACTGCATTGCCCTGGACGCTTAT
SPM-8      (2377) CTTTTAGAAACAGTTCAGATGTATTTGTCGCTGACCAGTGTGTTGGTGCTGAACGCCACTGCATTGCCCTGGACGCTTAT
SPM-16     (2377) CTTTTAGAAACAGTTCAGATGTATTTGTCGCTGACCAGTGTGTTGGTGCTGAACGCCACTGCATTGCCCTGGACGCTTAT
SPM-B1     (2018) --------------------------------------------------------------------------------
SPM-A28C    (475) CTTTTAGAAACAGTTCAGATGTATTTGTCGCTGACCAGTGTGTTGGTGCTGAACGCCACTGCATTGCCCTGGACGCTTAT

KM360184   (2721) ACCTACCTCCGTGTTGATTGTCATCTTCGTGCTCATGTTGAGATGGTACCTGAATACAGCTCAGGCTGTCAAACGTTTGG
SPM-8      (2457) ACCTACCTCCGTGTTGATTGTCATCTTCGTGCTCATGTTGAGATGGTACCTGAATACAGCTCAGGCTGTCAAACGTTTGG
SPM-16     (2457) ACCTACCTCCGTGTTGATTGTCATCTTCGTGCTCATGTTGAGATGGTACCTGAATACAGCTCAGGCTGTCAAACGTTTGG
SPM-B1     (2018) --------------------------------------------------------------------------------
SPM-A28C    (555) ACCTACCTCCGTGTTGATTGTCATCTTCGTGCTCATGTTGAGATGGTACCTGAATACAGCTCAGGCTGTCAAACGTTTGG

KM360184   (2801) AGGGCACAACCAAGAGTCCTGTATTTGGAATGATTAACTCCACTATTTCTGGACTCTCCACTATTAGAAGTTCGGGTTCC
SPM-8      (2537) AGGGCACAACCAAGAGTCCTGTATTTGGAATGATTAACTCCACTATTTCTGGACTCTCCACTATTAGAAGTTCGGGTTCC
```

```
SPM-16   (2537) AGGGCACAACCAAGAGTCCTGTATTTGGAATGATTAACTCCACTATTTCTGGACTCTCCACTATTAGAAGTTCGGGTTCC
SPM-B1   (2018) --------------------------------------------------------------------------------
SPM-A28C  (635) AGGGCACAACCAAGAGTCCTGTATTTGGAATGATTAACTCCACTATTTCTGGACTCTCCACTATTAGAAGTTCGGGTTCC

KM360184 (2881) CAGGATAGACAGATGAAATTGTTTGACGAAGCGCAGAATCTCCACACAAGTGCTTTCCACACATTCTTCGGCGGTTCTAC
SPM-8    (2617) CAGGATAGACAGATGAAATTGTTTGACGAAGCGCAGAATCTCCACACAAGTGCTTTCCACACATTCTTCGGCGGTTCTAC
SPM-16   (2617) CAGGATAGACAGATGAAATTGTTTGACGAAGCGCAGAATCTCCACACAAGTGCTTTCCACACATTCTTCGGCGGTTCTAC
SPM-B1   (2018) --------------------------------------------------------------------------------
SPM-A28C  (715) CAGGATAGACAGATGAAATTGTTTGACGAAGCGCAGAATCTCCACACAAGTGCTTTCCACACATTCTTCGGCGGTTCTAC

KM360184 (2961) GGCATTTGCATTGTATCTCGATACTTTGTGTTTGACCTACCTCGGTGTCGTCATGTCAATTTTCATTTTGGGCGACTTTG
SPM-8    (2697) GGCATTTGCATTGTATCTCGATACTTTGTGTTTGACCTACCTCGGTGTCGTCATGTCAATTTTCATTTTGGGCGACTTTG
SPM-16   (2697) GGCATTTGCATTGTATCTCGATACTTTGTGTTTGACCTACCTCGGTGTCGTCATGTCAATTTTCATTTTGGGCGACTTTG
SPM-B1   (2018) --------------------------------------------------------------------------------
SPM-A28C  (795) GGCATTTGCATTGTATCTCGATACTTTGTGTTTGACCTACCTCGGTGTCGTCATGTCAATTTTCATTTTGGGCGACTTTG

KM360184 (3041) GTGATTTGATCCCGGTGGGAAGTGTCGGTCTGGCTGTCGGTCAGTCCATGGTGCTGACCATGATGTTGCAGATGGCAGCT
SPM-8    (2777) GTGATTTGATCCCGGTGGGAAGTGTCGGTCTGGCTGTCAGTCAGTCCATGGTGCTGACCATGATGTTGCAGATGGCAGCT
SPM-16   (2777) GTGATTTGATCCCGGTGGGAAGTGTCGGTCTGGCTGTCAGTCAGTCCATGGTGCTGACCATGATGTTGCAGATGGCAGCT
SPM-B1   (2018) GTGTTTTGATCCCGGTGGGAAGTGTCGGTCTGGCTGTCAGTCAGTCCATGGTGCTGACCATGATGTTGCAGATGGCATTT
SPM-A28C  (875) GTGATTTGATCCCGGTGGGAAGTGTCGGTCTGGCTGTCAGTCAGTCCATGGTGCTGACCATGATGTTGCAGATGGCAGCT

KM360184 (3121) AGGTCCACAGCTGACTTTTTGGGGCAAATGACGGCTGTGGAGAGAGTTCTGGAATACACCAAGCTACCCACGGAGACCAA
SPM-8    (2857) AGGTTCACAGCTGACTTTTTGGGGCAAATGACGGCTGTGGAGAGAGTTCTGGAATACACCAAGCTACCCACGGAGACCAA
SPM-16   (2857) AGGTTCACAGCTGACTTTTTGGGGCAAATGACGGCTGTGGAGAGAGTTCTGGAATACACCAAGCTACCCACGGAGACCAA
SPM-B1   (2095) AGGTTCACAGCTGACTTTTTGGGGCAAATGACGGCTGTGGAGAGAGTTCTGGAAAACACCCAGCTACCCACGG-------
SPM-A28C  (955) AGGTTCACAGCTGACTTTTTGGGGCAAATGACGGCTGTGGAGAGAGTTCTGGAATACACCAAGCTACCCACGGAGACCAA

KM360184 (3201) TATGGAGCAAGGACCAACTAACCCACCAAAGGAATGGCCCAGTGCTGGTAGAGTGACGTTCTCAAATGTGTACCTGAATT
SPM-8    (2937) TATGGAGCAAGGACCAACTAACCCACCAAAGGAATGGCCCAGTGCTGGTAGAGTGACGTTCTCAAATGTGTACCTGAATT
SPM-16   (2937) TATGGAGCAAGGACCAACTAACCCACCAAAGGAATGGCCCAGTGCTGGTAGAGTGACGTTCTCAAATGTGTACCTGAATT
SPM-B1   (2168) ---GGAGCAAGGAC------------------GAATGGCCCAGTGCTGGTAGAGTGACGTTCTCAAATGTGTACCTGAATT
SPM-A28C (1035) TATGGAGCAAGGACCAACTAACCCACCAAAGGAATGGCCCAGTGCTGGTAGAGTGACGTTCTCAAATGTGTACCTGAATT

KM360184 (3281) ATTCCATGGAAGACCCGCCGGTGCTGAAGGACTTAAACTTTGAAATTCAAAGCGGCTGGAAGGTTGGAGTTGTAGGCAGA
SPM-8    (3017) ATTCCATGGAAGACCCGCCGGTGCTGAAGGACTTAAACTTTGAAATTCAAAGCGGCTGGAAGGTTGGAGTTGTAGGCAGA
SPM-16   (3017) ATTCCATGGAAGACCCGCCGGTGCTGAAGGACTTAAACTTTGAAATTCAAAGCGGCTGGAAGGTTGGAGTTGTAGGCAGA
SPM-B1   (2228) ATTCCATGGAAGACCCGCCGGTGCTGAAGGACTTAAACTTTGAAATTCAAAGCGGCTGGAAGGTTGGAGTTGTAGGCAGA
SPM-A28C (1115) ATTCCATGGAAGACCCGCCGGTGCTGAAGGACTTAAACTTTGAAATTCAAAGCGGCTGGAAGGTTGGAGTTGTAGGCAGA

KM360184 (3361) ACAGGAGCCGGCAAGTCATCGCTCA--------------------TCGCGGCTTTGTTCCGGCTTAGTGACATAAGCGGC
SPM-8    (3097) ACAGGAGCCGGCAAGTCATCGCTC--------------------------------------------------------
SPM-16   (3097) ACAGGAGCCGGCAAGTCATCAC--A------------------------------------------TGAAATTAAATAC
SPM-B1   (2308) ACAGGAGCCGGCAAGTCATCGCTCA--------------------TCGCGGCTTTGTTCCGGCTTAGTGACATAAGCGGC
SPM-A28C (1195) ACAGGAGCCGGCAAGTCATCGCTCACGGCTTTGTTCTCTTTGTTCTCGCGGCTTTGTTCCGGCTTAGTGACATAAGCGGC

KM360184 (3421) AGCATCAAAATTGACGGTGTGGACACCGAAGGATTAGCCAAAAAGACTTTGAGATCGAAAATATCAATTATTCCACAAGA
SPM-8    (3121) --------------------------------------------------------------------------------
SPM-16   (3133) GTTG-----ATATACTG-GTTAATAC------ATTTGTAGGAAAAACTTTGAGATCGAAAATATCAATTATTCCACAAGA
SPM-B1   (2368) AGCATCAAAATTGACGGTGTGGACACCGAAGGATTAGCCAAAAAGACTTTGAGATCGAAAATATCAATTATTCCACAAGA
SPM-A28C (1275) AGCATCAAAATTGACGGTGTGGACACCGAAGGATTAGCCAAAAAGACTTTGAGATCGAAAATATCAATTATTCCACAAGA

KM360184 (3501) GCCGGTGCTGTTCTCGGCTACTCTGCGATACAATTTGGACCCGTTCGACGATTACAGCGACGACGATATTT----GGAGG
SPM-8    (3121) ----------------------------------------------------------------------------GAGG
SPM-16   (3201) GCCGGTGCTGTTCTCGGCTACTCTGCGATACAATTTGGACCCGTTCGACGATTACAGCGACGACGATATTTATATGGAGG
SPM-B1   (2448) GCCGGTGCTGTTCTCGGCTACTCTGCGATACAATTTGGACCCGTTCGACGATTACAGCGACGACGATATTT----GGAGG
SPM-A28C (1355) GCCGGTGCTGTTCTCGGCTACTCTGCGATACAATTTGGACCCGTTCGACGATTACAGCGACGACGATATTT----GGAGG

KM360184 (3577) GCGTTGGAACAGGTGGAATTAAAAGAAGGAATACCGGCTTTAGACTTTAAGGTCGCTGAAGGTGGTACTAACTTCTCTAT
SPM-8    (3125) GCGTTGGAACAGGTGGAATTAAAAGAAGGAATACCGGCTTTAGACTTTAAGGTCGCTGAAGGTGGTACTAACTTCTCTAT
SPM-16   (3281) GCGTTGGAACAGGTGGAATTAAAAGAAGGAATACCGGCTTTAGACTTTAAGGTCGCTGAAGGTGGTACTAACTTCTCTAT
SPM-B1   (2524) GCGTTGGAACAGGTGGAATTAAAAGAAGGAATACCGGCTTTAGACTTTAAGGTCGCTGAAGGTGGTACTAACTTCTCTAT
SPM-A28C (1431) GCGTTGGAACAGGTGGAATTAAAAGAAGGAATACCGGCTTTAGACTTTAAGGTCGCTGAAGGTGGTACTAACTTCTCTAT

KM360184 (3657) GGGACAACGTCAGTTGGTTTGCTTGGCTCGTGCCATTCTACGCTCTAACAAAATACTCATCATGGACGAAGCTACCGCTA
SPM-8    (3205) GGGACAACGTCAGTTGGTTTGCTTGGCTCGTGCCATTCTACGCTCTAACAAAATACTCATCATGGACGAAGCTACCGCTA
SPM-16   (3361) GGGACAACGTCAGTTGGTTTGCTTGGCTCGTGCCATTCTACGCTCTAACAAAATACTCATCATGGACGAAGCTACCGCTA
SPM-B1   (2604) GGGACAACGTCAGTTGGTTTGCTTGGCTCGTGCCATTCTACGCTCTAACAAAATACTCATCATGGACGAAGCTACCGCTA
SPM-A28C (1511) GGGACAACGTCAGTTGGTTTGCTTGGCTCGTGCCATTCTACGCTCTAACAAAATACTCATCATGGACGAAGCTACCGCTA

KM360184 (3737) ACGTCGATCCTCAGACGGACGCTTTGATCCAAAAGACGATCCGTCGTCAGTTTGCGTCGTGCACGGTGCTCACCATCGCG
```

```
SPM-8    (3285) ACGTCGATCCTCAGACGGACGCTTTGATCCAAAAGACGATCCGTCGTCAGTTTGCGTCGTGCACGGTGCTCACCATCGCG
SPM-16   (3441) ACGTCGATCCTCAGACGGACGCTTTGATCCAAAAGACGATCCGTCGTCAGTTTGCGTCGTGCACGGTGCTCACCATCGCG
SPM-B1   (2684) ACGTCGATCCTCAGACGGACGCTTTGATCCAAAAGACGATCCGTCGTCAGTTTGCGTCGTGCACGGTGCTCACCATCGCG
SPM-A28C (1521) ACGTCGATCCTCAGACGGACGCTTTGATCCAAAAGACGATCCGTCGTCAGTTTGCGTCGTGCACGGTGCTCACCATCGCG

KM360184 (3817) CATCGACTGAACACCATCATGGACTCCGACCGAGTGCTGGTCATGGACCAGGGCGAAGTGGCCGAGTTCGACCATCCCCA
SPM-8    (3365) CATCGACTGAACACCATCATGGACTCCGACCGAGTGCTGGTCATGGACCAGGGCGAAGTGGCCGAGTTCGACCATCCCCA
SPM-16   (3521) CATCGACTGAACACCATCATGGACTCCGACCGAGTGCTGGTCATGGACCAGGGCGAAGTGGCCGAGTTCGACCATCCCCA
SPM-B1   (2764) CATCGACTGAACACCATCATGGACTCCGACCGAGTGCTGGTCATGGACCAGGGCGAAGTGGCCGAGTTCGACCATCCCCA
SPM-A28C (1521) CATCGACTGAACACCATCATGGACTCCGACCGAGTGCTGGTCATGGACCAGGGCGAAGTGGCCGAGTTCGACCATCCCCA

KM360184 (3897) CATCTTGCTCAGCAACCCCAATAGCAAGTTCTTCTCTATGGTCCGAGAGACAGGAGAAAGCATGACAAGGACCTTAATGG
SPM-8    (3445) CATCTTGCTCAGCAACCCCAATAGCAAGTTCTTCTCTATGGTCCGAGAGACAGGAGAAAGCATGACAAGGACCTTAATGG
SPM-16   (3601) CATCTTGCTCAGCAACCCCAATAGCAAGTTCTTCTCTATGGTCCGAGAGACAGGAGAAAGCATGACAAGGACCTTAATGG
SPM-B1   (2844) CATCTTGCTCAGCAACCCCAATAGCAAGTTCTTCTCTATGGTCCGAGAGACAGGAGAAAGCATGACAAGGACCTTAATGG
SPM-A28C (1521) CATCTTGCTCAGCAACCCCAATAGCAAGTTCTTCTCTATGGTCCGAGAGACAGGAGAAAGCATGACAAGGACCTTAATGG

KM360184  (3977) AGGTCGCTAAGGCCAAATATGATAGTGATAATAAGGAAGCTTAA
SPM-8     (3525) AGGTCGCTAAGGC-------------------------------
SPM-16    (3681) AGGTCGCTAAGGC-------------------------------
SPM-B1    (2924) AGGTCGCTAAGGC-------------------------------
SPM-A28C  (1521) AGGTCGCTAAGGC-------------------------------
```

Figure S5. Alignment of partial mRNA sequences of *Helicoverpa zea* ATP binding cassette transporter subfamily C2 (*ABCC2*) from knockout lines SPM-8, SPM-16, SPM-B1, and SPM-A28C with H. zea ABCC2 mRNA sequence KM360184 from GenBank.Identical nucleotides are shown in white text with black background and alignment gaps are indicated by hyphens.

```
KM360184    (1) MGVENKNNVQNAEGPARKTYKKPNILSRIFLWWMCPVLITHFNKRNVEESDLIPPSNLYNSERQGEYLERYWLAEIENAT
SPM-8       (1) --------------------------------------------------------------------------------
SPM-16      (1) --------------------------------------------------------------------------------
SPM-B1      (1) --------------------------------------------------------------------------------
SPM-A28C    (1) --------------------------------------------------------------------------------

KM360184   (81) IENREPSLWKALRKAYWVSYMPGAIFIIIQSAARTYQPLLFSQLLSYWSVDSEMTQQDAGLYALAMLGLNFVSMMCQHHN
SPM-8       (1) --------WKALRKAYWVSYMPGAIFIIIQSAARTYQPLLFSQLLSYWSVDSEMTQQDAGLYALAMLGLNFVSMMCQHHN
SPM-16      (1) --------WKALRKAYWVSYMPGAIFIIIQSAARTYQPLLFSQLLSYWSVDSEMTQQDAGLYALAMLGLNFVSMMCQHHN
SPM-B1      (1) --------WKALRKAYWVSYMPGAIFIIIQSAARTYQPLLFSQLLSYWSVDSEMTQQDAGLYALAMLGLNFVSMMCQHHN
SPM-A28C    (1) --------WKALRKAYWVSYMPGAIFIIIQSAARTYQPLLFSQLLSY---------------------------------

KM360184  (161) TLFVMRFSLKVKVACSSLLYRKLLRMTQVSVGEVAGGKLVNLLSNDITRFDYAFMFLHYLWIVPIQVAVVLYFLWEAAGF
SPM-8      (73) TLFVMRFSLKVKVACSSLLYRKLLRMTQVSVGEVAGGKLVNLLSNDITRFDYAFMFLHYLWIVPIQVAVVLYFLWEAAGF
SPM-16     (73) TLFVMRFSLKVKVACSSLLYRKLLRMTQVSVGEVAGGKLVNLLSNDITRFDYAFMFLHYLWIVPIQVAVVLYFLWEAAGF
SPM-B1     (73) TLFVMRFSLKVKVACSSLLYRKLLRMTQVSVGEVAGGKLVNLLSNDITRFDYAFMFLHYLWIVPIQVAVVLYFLWEAAGF
SPM-A28C   (40) --------------------------------------------------------------------------------

KM360184  (241) APFVGLFGVVILILPLQAGLTKLTTVVRRETAKRTDRRIKLMSEIIGGIQVIKMYAWEKPFQLVVKAARAFEMSALRKSI
SPM-8     (153) APFVGLFGVVILILPLQAGLTKLTTVVRRETAKRTDRRIKLMSEIIGGIQVIKMYAWEKPFQLVVKAARAFEMSALRKSI
SPM-16    (153) APFVGLFGVVILILPLQAGLTKLTTVVRRETAKRTDRRIKLMSEIIGGIQVIKMYAWEKPFQLVVKAARAFEMSALRKSI
SPM-B1    (153) APFVGLFGVVILILPLQAGLTKLTTVVRRETAKRTDRRIKLMSEIIGGIQVIKMYAWEKPFQLVVKAARAFEMSALRKSI
SPM-A28C   (40) --------------------------------------------------------------------------------

KM360184  (321) FIRSTFLGFMLFTERSIMFVTVLTLALTGTMITATTIYPIQQYFSIIQFNVTLIIPMAIASYSEMMVSIERIQGFLSLDE
SPM-8     (233) FIRSTFLGFMLFTERSIMFVTVLTLALTGTMITATTIYPIQQYFSIIQFNVTLIIPMAIASYSEMMVSIERIQGFLSLDE
SPM-16    (233) FIRSTFLGFMLFTERSIMFVTVLTLALTGTMITATTIYPIQQYFSIIQFNVTLIIPMAIASYSEMMVSIERIQGFLSLDE
SPM-B1    (233) FIRSTFLGFMLFTERSIMFVTVLTLALTGTMITATTIYPIQQYFSIIQFNVTLIIPMAIASYSEMMVSIERIQGFLSLDE
SPM-A28C   (40) --------------------------------------------------------------------------------

KM360184  (401) RSDMQVTPKMNGSNNNTLFKSKKSPLEVGIVPKKYSPSEVMAAKEMQDDPTQMDYPIRLNKVSASWTGSNSSSEMTLKNI
SPM-8     (313) RSDMQVTPKINGSNNNTLFKSKKSPLEVGIVPKKYSPSEVMAAKEMQDDPTQMDYPIRLNKVSASWTGSNSSSEMTLKNI
SPM-16    (313) RSDMQVTPKINGSNNNTLFKSKKSPLEVGIVPKKYSPSEVMAAKEMQDDPTQMDYPIRLNKVSASWTGSNSSSEMTLKNI
SPM-B1    (313) RSDMQVTPKINGSNNNTLFKSKKSPLEVGIVPKKYSPSEVMAAKEMQDDPTQMDYPIRLNKVSASWTGSNSSSEMTLKNI
SPM-A28C   (40) --------------------------------------------------------------------------------

KM360184  (481) SLRIRKGKLCAIIGPVGSGKTSLLQLLLKELPLNSGTLDVSGKMSYACQESWLFPGTVRENILFGLTYEPTKYKEVCKVC
SPM-8     (393) SLRIRKGKLCAIIGPVGSGKTSLLQLLLKELPLNSGTLDVSGKMSYACQESWLFPGTVRENILFGLTYEPTKYKEVCKVC
SPM-16    (393) SLRIRKGKLCAIIGPVGSGKTSLLQLLLKELPLNSGTLDVSGKMSYACQESWLFPGTVRENILFGLTYEPTKYKEVCKVC
SPM-B1    (393) SLRIRKGKLCAIIGPVGSGKTSLLQLLLKELPLNSGTLDVSGKMSYACQESWLFPGTVRENILFGLTYEPTKYKEVCKVC
SPM-A28C   (40) --------------------------------------------------------------------------------

KM360184  (561) SLLPDFKQFPYGDLSLVGERGVSLSGGQRARINLARAIYREADIYLLDDPLSAVDANVGRQLFDGCIKGYLTGRTCVLVT
SPM-8     (473) SLLPDFKQFPYGDLSLVGERGVSLSGGQRARINLARAIYREADIYLLDDPLSAVDANVGRQLFDGCIKGYLTGRTCVLVT
SPM-16    (473) SLLPDFKQFPYGDLSLVGERGVSLSGGQRARINLARAIYREADIYLLDDPLSAVDANVGRQLFDGCIKGYLTGRTCVLVT
SPM-B1    (473) SLLPDFKQFPYGDLSLVGERGVSLSGGQRARINLARAIYREADIYLLDDPLSAVDANVGRQLFDGCIKGYLTGRTCVLVT
SPM-A28C   (40) --------------------------------------------------------------------------------

KM360184  (641) HQIHYLKAADFIVVLNEGSVENMGTYDELVKTGTEFSMLLSNQENDATENEKKDRPAMMRGISKISVKSDTEMEQKAQIQ
SPM-8     (553) HQIHYLKAADFIVVLNEGSVENMGTYDELVKTGTEFSMLLSNQENDATENEKKDRPAMMRGISKISVKSDTEMEQKAQIQ
SPM-16    (553) HQIHYLKAADFIVVLNEGSVENMGTYDELVKTGTEFSMLLSNQENDATENEKKDRPAMMRGISKISVKSDTEMEQKAQIQ
SPM-B1    (553) HQIHYLKAADFIVVLNEGSVENMGTYDELVKTGTEFSMLLSNQENDATENEKKDRPAMMRGISKISVKSDTEMEQKAQIQ
SPM-A28C   (40) --------------------------------------------------------------------------------

KM360184  (721) EAEERATGSLKFEVVLKYLSSVQSWCLVFTAFLVLLITQGAATTADYWLSFWTNQVDSYEQSLPEGVDPDTDMNAQIGLL
SPM-8     (633) EAEERATGSLKFEVVLKYLSSVQSWCLVFTAFLVLLITQGAATTADYWLSFWTNQVDSYEQSLPEGVDPDTDMNAQIGLL
SPM-16    (633) EAEERATGSLKFEVVLKYLSSVQSWCLVFTAFLVLLITQGAATTADYWLSFWTNQVDSYEQSLPEGVDPDTDMNAQIGLL
SPM-B1    (633) EAEERATGSLKFEVVLKYLSSVQSWCLVFTAFLVLLITQ-----------------------------------------
SPM-A28C   (40) --------------------------------------------------------------------------------

KM360184  (801) TTAQYLYVFGGVILALIVMTLVRITAFVAMTMRASQNLHNTIYEKLIVTVMRFFDTNPSGRVLNRFSKDMGAMDELLPRS
SPM-8     (713) TTAQYLYVFGGVILALIVMTLVRITAFVAMTMRASQNLHNTIYEKLIVTVMRFFDTNPSGRVLNRFSKDMGAMDELLPRS
SPM-16    (713) TTAQYLYVFGGVILALIVMTLVRITAFVAMTMRASQNLHNTIYEKLIVTVMRFFDTNPSGRVLNRFSKDMGAMDELLPRS
SPM-B1    (672) --------------------------------------------------------------------------------
SPM-A28C   (40) --------------------------------------------------------------------------------

KM360184  (881) LLETVQMYLSLTSVLVLNATALPWTLIPTSVLIVIFVLMLRWYLNTAQAVKRLEGTTKSPVFGMINSTISGLSTIRSSGS
SPM-8     (793) LLETVQMYLSLTSVLVLNATALPWTLIPTSVLIVIFVLMLRWYLNTAQAVKRLEGTTKSPVFGMINSTISGLSTIRSSGS
SPM-16    (793) LLETVQMYLSLTSVLVLNATALPWTLIPTSVLIVIFVLMLRWYLNTAQAVKRLEGTTKSPVFGMINSTISGLSTIRSSGS
SPM-B1    (672) --------------------------------------------------------------------------------
SPM-A28C   (40) --------------------------------------------------------------------------------
```

```
KM360184  (961) QDRQMKLFDEAQNLHTSAFHTFFGGSTAFALYLDTLCLTYLGVVMSIFILGDFGDLIPVGSVGLAVGQSMVLTMMLQMAA
SPM-8     (873) QDRQMKLFDEAQNLHTSAFHTFFGGSTAFALYLDTLCLTYLGVVMSIFILGDFGDLIPVGSVGLAVSQSMVLTMMLQMAA
SPM-16    (873) QDRQMKLFDEAQNLHTSAFHTFFGGSTAFALYLDTLCLTYLGVVMSIFILGDFGDLIPVGSVGLAVSQSMVLTMMLQMAA
SPM-B1    (672) -----------------------------------------------------GVLIPVGSVGLAVSQSMVLTMMLQMAF
SPM-A28C   (40) --------------------------------------------------------------------------------

KM360184 (1041) RSTADFLGQMTAVERVLEYTKLPTETNMEQGPTNPPKEWPSAGRVTFSNVYLNYSMEDPPVLKDLNFEIQSGWKVGVVGR
SPM-8     (953) RFTADFLGQMTAVERVLEYTKLPTETNMEQGPTNPPKEWPSAGRVTFSNVYLNYSMEDPPVLKDLNFEIQSGWKVGVVGR
SPM-16    (953) RFTADFLGQMTAVERVLEYTKLPTETNMEQGPTNPPKEWPSAGRVTFSNVYLNYSMEDPPVLKDLNFEIQSGWKVGVVGR
SPM-B1    (699) RFTADFLGQMTAVERVLENTQLPTGSKD---------EWPSAGRVTFSNVYLNYSMEDPPVLKDLNFEIQSGWKVGVVGR
SPM-A28C   (40) --------------------------------------------------------------------------------

KM360184 (1121) TGAGKSSLIAALFRLSDISGSIKIDGVDTEGLAKKTLRSKISIIPQEPVLFSATLRYNLDPFDDYSDDDIWRALEQVELK
SPM-8    (1033) TGAGKSSLEGVGTGGIKRRNTGFRL-------------------------------------------------------
SPM-16   (1033) TGAGKSSHEIKYVDILVNTFVG------------KTLRSKISIIPQEPVLFSATLRYNLDPFDDYSDDDIYMEGVGTGGI
SPM-B1    (770) TGAGKSSLIAALFRLSDISGSIKIDGVDTEGLAKKTLRSKISIIPQEPVLFSATLRYNLDPFDDYSDDDIWRALEQVELK
SPM-A28C   (40) --------------------------------------------------------------------------------

KM360184 (1201) EGIPALDFKVAEGGTNFSMGQRQLVCLARAILRSNKILIMDEATANVDPQTDALIQKTIRRQFASCTVLTIAHRLNTIMD
SPM-8    (1058) --------------------------------------------------------------------------------
SPM-16   (1101) KRRNTGFRL-----------------------------------------------------------------------
SPM-B1    (850) EGIPALDFKVAEGGTNFSMGQRQLVCLARAILRSNKILIMDEATANVDPQTDALIQKTIRRQFASCTVLTIAHRLNTIMD
SPM-A28C   (40) --------------------------------------------------------------------------------

KM360184 (1281) SDRVLVMDQGEVAEFDHPHILLSNPNSKFFSMVRETGESMTRTLMEVAKAKYDSDNKEA
SPM-8    (1058) -----------------------------------------------------------
SPM-16   (1110) -----------------------------------------------------------
SPM-B1    (930) SDRVLVMDQGEVAEFDHPHILLSNPNSKFFSMVRETGESMTRTLMEVAK----------
SPM-A28C   (40) -----------------------------------------------------------
```

Figure S6. Alignment of putative amino acid sequences predicted from partial mRNA sequences of *Helicoverpa zea* ATP binding cassette transporter subfamily C2 (*ABCC2*) gene knockout lines SPM-8, SPM-16, SPM-B1, and SPM-A28C with the ABCC2 amino acid sequence AKH49600 from GenBank. Identical amino acids are shown by white text in black background and alignment gaps are shown with a hyphen.

A.

```
KZ117297_   AGTTTAGATAAGCTCAATAACTGTGTTACCACGGAGACCAATGAATCACACAGAAGAGACTAATGATAGATAGATAATA
WT          AGTTTAGATAAGCTTAATAACTGTGTTACCACGGAGACCAATGAATCACACAGAAGAGACTAATGATAGATAGATAATA
SPM-28C     AGTTTAGATAAGCTTAATAACTGTGTTACCACGGAGACCAATGAATCACACAGAAGAGACTAATGATAGATAGATAATA
SPM-B1      AGTTTAGATAAGCTTAATAACTGTGTTACCACGGAGACCAATGAATCACACAGAAGAGACTAATGATAGATAGATAATA
```

B.

```
KZ117297    CGAATGCGCACATAATTTTATATCTAAACCACGGAGAACAAAATTACTAGCGGAGGTGCCATAGTGGAAAATCGCCTAA
WT          CGAATGCGCACATAATTTTATATCTAAACCACGGAGAACTAAATTACTAGCGGAGGTGCCATAGTGGAAAATCGCCTAA
SPM-28C     CGAATGCGCACATAATTTTATATCTAAACCACGGAGAACAAAATTACTAGCGGAGGTGCCATAGTGGAAAATCGCCTAA
SPM-B1      CGAATGCGCACATAATTTTATATCTAAACCACGGAGAACAAAATTACTAGCGGAGGTGCCATAGTGGAAAATCGCCTAA
```

C.

```
KZ117617    GAATTTGATATTGACTTTTAAGCCCTCTCCACGGAGACCAATAAGAGTAAGACACAGAGTAAGGAAAGATGAGTGGAGA
WT          GAATTTGATATTGACTTTTAAGCCCTCTCCACGGAGACCAATAAGAGTAAGACACAGAGTAAGGAAAGATGAGTGGAGA
SPM-28C     GAATTTGATATTGACTTTTAAGCCCTCTCCACGGAGACCAATAAGAGTAAGACACAGAGTAAGGAAAGATGAGTGGAGA
SPM-B1      GAATTTGATATTGACTTTTAAGCCCTCTCCACGGAGACCAATAAGAGTAAGACACAGAGTAAGGAAAGATGAGTGGAGA
```

D.

```
KZ118710    CGCAATAGTATTTTTAAATAAATAAACGCAATAACTAATAGTCGGCAGTGGTTCGCTTGCTCCGTGTCTGGCACTTGGG
WT          CGCAATAGTATTTTTAAATAAATAAACGCAATAACTAATAGTCGGCAGTGGTTCGCTTGCTCCGTGTCTGGCACTTGGG
SPM-28C     CGCAATAGTATTTTTAAATAAATAAACGCAATAACTAATAGTCGGCAGTGGTTCGCTTGCTCCGTGTCTGGCACTTGGG
SPM-B1      CGCAATAGTATTTTTAAATAAATAAACGCAATAACTAATAGTCGGCAGTGGTTCGCTTGCTCCGTGTCTGGCACTTGGG
```

Figure S7. Alignment of nucleotide sequences from potential off-target sites for exon 13 and exon 19 sgRNA in *Helicoverpa zea* ATP binding cassette transporter subfamily C2 (*ABCC2*) knockout lines SPM-A28C and SPM-B1. Analysis of potential off-targets for sgRNA for exon 13 (5′-CTGCCGACTATTGGTTGAGT-3′) and exon 19 (5′- TTGCTCCATATTGGTCTCCG-3′) in *Helicoverpa zea* ABCC2 gene knockout lines SPM-A28C and SPM-B1. Off-targets for exon 19 sgRNA located in genomic scaffold KZ117297 were examined by amplifying genomic DNA from wild type insects and knockout lines using primers 4032 (5′-CTGAACACAAATGCGCAAATCG-3′) and 4033 (5′-CGTGTTTACGACATGCCGAT-3′) (**A**), and 4034 (5′-ACGCGTTTAATGAGTACAACCG-3′) and 4035 (5′-ATGCAGTAGGTTAGGCGCCT-3′) (**B**). Primer pair 4036 (5′-TTATGACTTGGTTGCCACAACC-3′) and 4037 (5′-CGCCCTTATAGGTACGCCTT-3′) was used to amplify the potential off-target site for exon 19 sgRNA located in *H. zea* genomic scaffold KZ1176187 (**C**). Primer pair 4038 (5′-CAGGTCCTAAATATTCATGCACC-3′) and 4039 (5′-CATGGCCTTTGTTTGTGGAT-3′) was used to amplify the potential off target site for exon 13 sgRNA located in the in the *H. zea* genomic scaffold KZ118710 (**D**). Identical nucleotides are shown in white text in black background and variations from the reference sequence are shown by black text. Potential off-target sequences and protospacer adjacent sequence (PAM) are marked by black text and red text in green background, respectively.

Table S1. Primer names and sequences used for PCR amplification and nucleotide sequencing of *Helicoverpa zea* ATP binding cassette transporter subfamily C2 (*ABCC2*) gene and off-target sites. The last letter of the name indicates the forward (F) or reverse (R) direction or the primer.

Primer name	Primer sequence	purpose
3099HzABC2_Ex15R	GAAAGCTGTGATTCTGACGA	PCR/Sequencing ABCC2
3104HzABC2_Ex19F	GTGGGAAGTGTCGGTCTGGC	PCR/Sequencing ABCC2
3105HzABC2_Ex20F	GGAAGACCCGCCGGTGCTGA	PCR/Sequencing ABCC2
3771Hz_ABCC2_Ex21F	TAGGTTGGAGTTGTAGGCAGAAC	Sequencing ABCC2
3772Hz_ABCC2_Int21R	AGGGATGCTAACGGATCATT	Sequencing ABCC2
3776Hz_ABCC2_Ex23R	CGGTAGCTTCGTCCATGATG	Sequencing ABCC2
3790Hz_ABCC2_94063F	GCTGTTTGGCGATAAGACGGC	PCR/Sequencing ABCC2
3791Hz_ABCC2_96886R	CAGCGACCTTAAAGTCTAAAGCCG	PCR/Sequencing ABCC2
3800Hz_ABCC2Ex2F	TGGAAGGCATTACGAAAGGC	PCR/Sequencing ABCC2
3801Hz_ABCC2Ex3R	CGAAGAACAGGCAACCTTGACT	PCR/Sequencing ABCC2
3802Hz_ABCC2Ex8F	GAGCGGTCCGACATGCAAGT	PCR/Sequencing ABCC2
3803Hz_ABCC2Ex8R	GACCCCACAGGACCAATGAT	PCR/Sequencing ABCC2
3804Hz_ABCC2Ex13F	GACCGACCAGCAATGATGCG	PCR/Sequencing ABCC2
3805Hz_ABCC2Ex14R	GGATCCACGCCTTCAGGTAA	PCR/Sequencing ABCC2
3806Hz_ABCC2Ex19F	GTGATTTGATCCCGGTGGGA	PCR/Sequencing ABCC2
3807Hz_ABCC2Ex20R	GCCATTCCTTTGGTGGGTTA	PCR/Sequencing ABCC2
3808Hz_ABCC2Ex23F	CATCATGGACGAAGCTACCG	PCR/Sequencing ABCC2
3809Hz_ABCC2Ex24R	GCCTTAGCGACCTCCATTAAG	PCR/Sequencing ABCC2
4032Hz_Scf_18_190197F	CTGAACACAAATGCGCAAATCG	PCR/Sequencing off targets
4033Hz_Scf_18_190612R	CGTGTTTACGACATGCCGAT	PCR/Sequencing off target
4034Hz_Scf_18_204300F	ACGCGTTTAATGAGTACAACCG	PCR/Sequencing off target
4035Hz_Scf_18_204763R	ATGCAGTAGGTTAGGCGCCT	PCR/Sequencing off target
4036Hz_Scf219_70159F	TTATGACTTGGTTGCCACAACC	PCR/Sequencing off target
4037Hz_Scf219_70569R	CGCCCTTATAGGTACGCCTT	PCR/Sequencing off target
4038Hz_Scf86_289141F	CAGGTCCTAAATATTCATGCACC	PCR/Sequencing off target
4039Hz_Scf86_289549R	CATGGCCTTTGTTTGTGGAT	PCR/Sequencing off target

Table S2: : Functional domains predicted in the ATP binding cassette transporter subfamily C2 (ABCC2) protein of *Helicoverpa zea* predicted using online Simple Modular Architecture Research Tool (SMART: http://smart.embl-heidelberg.de, accessed on 16 August 2021)

**Name of the region**	**Start**	**End**	**Domain**	**E-Value**
Transmembrane region	96	113	TMD1	N/A
Transmembrane region	139	156	TMD1	N/A
Transmembrane region	212	234	TMD1	N/A
Transmembrane region	238	260	TMD1	N/A
Transmembrane region	336	358	TMD1	N/A
Transmembrane region	363	382	TMD1	N/A
AAA-Nucleotide Binding	486	659	NBD1	7.96e-11
Transmembrane region	741	763	TMD2	N/A
Transmembrane region	805	827	TMD2	N/A
Transmembrane region	894	916	TMD2	N/A
Transmembrane region	988	1010	TMD2	N/A
Transmembrane region	1017	1039	TMD2	N/A
AAA-Nucleotide Binding	1111	1291	NBD2	6.12e-12

Table S3. The number of eggs injected, percent larvae hatched, the number of males and females, and the total adults recovered from three rounds of embryo injections with sgRNA/Cas9 nucleoprotein complexes targeting ATP binding cassette transporter subfamily C2 (*ABCC2*) gene in *Helicoverpa zea.*

Injection session	Total Eggs Injected	Total hatched	% Hatch	Males	Females	Dead larvae and pupae	% Adults (from injected eggs)
1	174	74	42.5	30	31	13	35.1
2A	167	37	22.2	15	14	8	17.4
2B	65	12	18.5	4	5	3	13.8
Total	406	123	30.3	47	47	29	23.2
